# Influence of host genotype in establishing root associated microbiome of *indica* rice cultivars for plant growth promotion

**DOI:** 10.3389/fmicb.2022.1033158

**Published:** 2022-11-14

**Authors:** Arjun Singh, Murugan Kumar, Hillol Chakdar, Kuppusamy Pandiyan, Shiv Charan Kumar, Mohammad Tarique Zeyad, Bansh Narayan Singh, K. T. Ravikiran, Arunima Mahto, Alok Kumar Srivastava, Anil Kumar Saxena

**Affiliations:** ^1^ICAR-National Bureau of Agriculturally Important Microorganisms, Mau, India; ^2^ICAR-Central Soil Salinity Research Institute, RRS, Lucknow, India; ^3^Ginning Training Center, ICAR-Central Institute for Research on Cotton Technology, Nagpur, India; ^4^National Institute of Plant Genome Research, New Delhi, India

**Keywords:** *indica* rice, community metagenomics, machine learning, SynComs, PiCRUST

## Abstract

Rice plants display a unique root ecosystem comprising oxic-anoxic zones, harboring a plethora of metabolic interactions mediated by its root microbiome. Since agricultural land is limited, an increase in rice production will rely on novel methods of yield enhancement. The nascent concept of tailoring plant phenotype through the intervention of synthetic microbial communities (SynComs) is inspired by the genetics and ecology of core rhizobiome. In this direction, we have studied structural and functional variations in the root microbiome of 10 *indica* rice varieties. The studies on α and β-diversity indices of rhizospheric root microbiome with the host genotypes revealed variations in the structuring of root microbiome as well as a strong association with the host genotypes. Biomarker discovery, using machine learning, highlighted members of class *Anaerolineae*, *α-Proteobacteria*, and bacterial genera like *Desulfobacteria*, *Ca*. *Entotheonella*, *Algoriphagus*, etc. as the most important features of *indica* rice microbiota having a role in improving the plant’s fitness. Metabolically, rice rhizobiomes showed an abundance of genes related to sulfur oxidation and reduction, biofilm production, nitrogen fixation, denitrification, and phosphorus metabolism. This comparative study of rhizobiomes has outlined the taxonomic composition and functional diversification of rice rhizobiome, laying the foundation for the development of next-generation microbiome-based technologies for yield enhancement in rice and other crops.

## Introduction

An increase in anthropogenic activities and dependency on inorganic agriculture for obtaining maximum crop yields over time, has led to the deterioration of soil quality. According to a report by Food and Agriculture Organization (FAO), at the current rate of soil erosions and climate change, 20–80% of the crop yield will be affected in the future.^1^ Microorganisms are an essential component of the soil ecosystem, and through various biogeochemical cycles provide sustenance to all the food crops consumed worldwide (Kopittke et al., 2019). Crop plants depend on the syntropic interaction with their below-ground microflora or rhizobiome, which provide resistance against an array of stressors such as drought, salinity, heavy metal pollution, and biotic stresses. Next-generation omics approaches have provided new insights into the biodiversity of soil-dwelling microbes and revealed that about 1,000 Gbp of microbial genomic content is available per gram of soil (Vogel et al., 2009; Friedman and Alm, 2012; Azaroual et al., 2022).

Soil microbial communities act in synchrony to maintain soil fertility, biogeochemical cycling of nutrients, detoxification, and carbon sequestration (Banerjee and van der Heijden, 2022). Plant root exudates consist of various signaling molecules, dissolved organic matter, volatile organic compounds (VOCs), etc., which tend to attract diverse groups of microbial communities. These in turn support the growth and performance of the assembling host by acting as hotspot regions of many biogeochemical cycles (Hu et al., 2018; Singh et al., 2020). Advancements in the area of plant-microbe interactions have shown that host varieties influence their associative soil microbial communities, which in turn regulate many of the plant phenetic traits. For instance, rhizobiome can help their host to survive abiotic stresses by changing root morphology, delaying senescence, and reducing heavy metal bioavailability to the host plant (Dodd et al., 2010; Deepthi et al., 2014). Rhizobiome also provides protection against biotic stresses by competitively interacting with the invading pathogenic species and hindering their colonization (Wei et al., 2015; del Carmen Orozco-Mosqueda et al., 2022). Recent studies, using systematic targeted metagenomic and high throughput data analyses, have provided deeper insights into plant-microbiome interactions. The first study conducted on *Arabidopsis thaliana* revealed that microbiota colonizing its rhizosphere has a significant impact on plant growth, productivity, and carbon sequestration (Lundberg et al., 2012). It was also found that the assemblage and composition of root microbiota were conditioned by the *Arabidopsis* variety. Similar outcomes and observations were reported for root microbiome studies conducted on other terrestrial plants (Fitzpatrick et al., 2018). More recently, the impact of agriculturally intensive cereal crops in shaping soil microbiome diversity is also being explored, concerning their influence on physiological growth and development. A study conducted on wheat to understand the effect of nitrogen fertilization on the rhizosphere microbial community showed that an increase in N fertilization level decreases microbial community variations. It was also observed that organic acid secretions present in root exudates promote the assemblage of beneficial bacteria around the root zones (Chen et al., 2019). Another study on root microbiome of maize revealed that genetic makeup of maize strongly influences shaping of the microbiome. Studies have also shown that these microbial communities can impart psychrotolerance to host (Beirinckx et al., 2020). Barley root microbiome study revealed that the assembled microbiome was involved in functions such as detoxification, nutrient mobilization, siderophores production, etc (Bulgarelli et al., 2015). Among fruit crops, studies on grapevine rootstock have shown that underground microflora had activities related to ACC deaminase, P-solubilization, and production of siderophore, and indole acetic acid, which benefit plant growth and development (Marasco et al., 2018). Concerning leguminous crops, the rhizosphere microbiome of *Phaseolus vulgaris* (common bean), *Vigna unguiculata* (cowpea), and *P*. *lunatus* (lima bean) have also been deciphered. The findings revealed that the cultivation of common beans on native soils increased the rhizosphere bacterial diversity as compared to agricultural soils. The nodule microbiomes of cowpea and lima bean were not affected by the application of compost or prevailing soil physico-chemical characteristics (Rocha et al., 2020).

Rice is a major food crop grown globally that feeds about 50% of the world’s population. In India, rice is grown in more than 45.1 Mha with a total production of 127.93 million hectares (Mha) (www.indiabudget.gov.in). Most of its physiological growth period requires submerged conditions, which result in the development of oxic-anoxic zones around rice roots; oxygenated zones around its rhizosphere, and anoxic zone farther to roots (Yuan et al., 2016; Zhao et al., 2019). This oxic-anoxic interface of the rice root rhizosphere supports the colonization of diverse aerobic, anaerobic or facultative anaerobic microbes (Revsbech et al., 1999; Li and Wang, 2013). Since it is an important crop with a vast global presence and peculiar growth requirements, several attempts have been made to comprehend the structural and functional diversity of its rhizosphere-associated microflora. Among the underlying factors which shape the rhizosphere microflora, the age of rice plants and root exudates composition are highly important (Aulakh et al., 2001). Previously, a culture-dependent study revealed that rice root and seed microbiota were comprised of *Bacillus firmus*, *B*. *fusiformis*, *B*. *pumilus*, *B*. *velezensis*, *Caulobacter crescentus*, *Kocuria palustris*, *Nitrobacter* spp. and *Nitrosospira* spp. (Banik et al., 2016; Hwangbo et al., 2016). These bacterial strains were capable of performing biogeochemical cycling of phosphorus and nitrogen, and also had antagonistic properties against various plant pathogens. High throughput sequencing technologies have accelerated these studies presenting new insights into the diversity of non-cultivable bacterial flora associated with rice. Metagenomic studies indicate that the composition of the rice rhizosphere microflora is the interactive effect of rice varieties with their growth environments (Edwards et al., 2015; Oliveira et al., 2022). Functional annotations of rhizospheric bacterial flora indicate their involvement in the biogeochemical cycling of nutrients, biocontrol, and plant growth promotions (Raaijmakers and Mazzola, 2012). Furthermore, culture-independent studies indicate that rice varieties also influence the flux of methane, and can be categorized into low and high methane emitters based on the abundance of methanotrophs in their rhizobiome (Liechty et al., 2020). The concept of rhizospheric influence was furthered when microbial communities of *indica* rice varieties were tailored into SynCom-based formulations for improving nitrogen use efficiency (Zhang et al., 2019).

India is the world’s second-largest producer of rice with current production of 127.93 million tonnes. It is grown in almost all the agro-climatic zones of the country covering a total acreage of 45.1 Mha. In this study, we have characterized the rhizobiome of ten popularly grown *indica* rice varieties (non-Basmati of *indica* ecotype) *viz*., MTU1001, BPT5204, CO52, HUR105, HUR917, MTU7029, SHIATS1, TKM13, Warangal 3,207 and Rajender Sweta. The study is intended to understand the influence of these varieties on the abundance of key bacterial species for regulation of biogeochemical/transformations of plant nutrients, especially carbon and nitrogen, methanogenesis and other plant growth promotional attributes. The results from this study will provide new prospects for the development of microbiome-based technologies for improving rice productivity and sustainability.

## Materials and methods

### Soil sampling and DNA isolation

For this study, 10 high-yielding, popular *indica* rice varieties, *viz*., MTU1001, BPT5204, CO52, HUR105, HUR917, MTU7029, SHIATS1, TKM13, Warangal 3,207, and Rajender Sweta, were grown during Kharif season, in separate experimental plots at Indian Institute of Seed Sciences, Mau, Uttar Pradesh, India (25.89 °N and 83.48 °E). All the rice varieties were grown following a uniform set of agronomic cultivation practices. Similar doses of fertilizer were applied to fulfil the nutrient requirement of the crop; nitrogen was applied at @ 130 kg/ha, whereas phosphorous @ 70 kg/ha was applied, and potassium @ 60 kg/ha was applied (Kaga et al., 2009). Before the transplantation of the rice seedlings, organic carbon and available nitrogen content of the soil was also estimated following the protocol of Walkley and Black (1934) and Subbaiah (1956). Field soil was having an organic carbon content of 0.51 ± 0.09% and available nitrogen was estimated to be 0.01 ± 0.001%. The rhizosphere soil was sampled at the tillering stage. Plants were uprooted and the soil attached to the roots was collected. The obtained 30 soil samples were air dried and sieved. DNA was isolated from these soil samples using the FastDNA™ SPIN Soil kit (MP Biomedicals, Solon, United States) according to the manufacturer’s protocol. For improving DNA yield, the initial bead beating step was extended to 15 min. The quality of DNA was determined on agarose gel (1% w/v) and its concentration was estimated using Qubit Fluorimeter (v.3.0).

### Targeted PCR amplification of 16S rRNA and next generation sequencing of soil DNA

Soil DNA was used for 16S rRNA gene amplification using V3 and V4 primer sets (V3F-5’ CCTACGGGNBGCASCAG3’; V4R-5’ GACTACNVGGGTATCTAATCC3’) (Takahashi et al., 2014). The amplified products were checked by running on 2% (w/v) agarose gel. The PCR products were purified for paired-end library preparation. Approximately 5 ng/μL of the PCR amplicon was used for library preparation using the NEB Next Ultra DNA library preparation kit (New England Biolabs, United Kingdom), as per the manufacturer’s protocol. Library size and quality were determined using Agilent 2200, TapeStation (Agilent Technologies, United States). High-throughput sequencing was performed using Illumina HiSeq 2500 platform with 2*250 cycle chemistry to obtain the metagenomic data.

### Analysis of metagenome sequence data

The raw data obtained from sequencing were processed and analyzed using the QIIME2 pipeline. Briefly, the *de novo* sequence quality filtering, chimera removal, and error corrections were done by deblur. The amplicon sequence variants (ASVs) generated were used to calculate phylogenetic distance based α and β-diversity indices, i.e., Faith phylogenetic diversity indices, and unweighted and weighted Unifrac distances. The relatedness of the groups was estimated by PERMANOVA using non-metric multi-dimensional scaling ordination (NMDS). For taxonomic annotations, the green genes database (version 13–8-99) was used at 99% sequence similarity.

### Metagenome reconstruction to ascertain functional relevance of rhizobiome

The PICRUSt2 pipeline was used for functional annotation of the rhizobiome of rice varieties (Douglas et al., 2020). To run this analysis, the PICRUSt2 plugin for QIIME 2 was obtained. As an input, feature tables as well as feature sequences obtained during the analysis were used. A Default NSTI cut-off of 2 was used throughout the analysis. After analysis, the datasets get predicted for KEGG orthologues, EC metagenome predictors, and MetaCyc pathway abundance predictors. The KEGG orthologues were used for further analysis and predictions. The taxonomic to phenotypic mapping of the datasets was done using the Metagenassist pipeline (Arndt et al., 2012).

### Identification of taxonomic and functional bioindicators

For the identification of key bacterial features associated with rice rhizobiome, a random forest machine learning approach was used. This involved Classification and Regression Tree to make decision trees for sub-setting the samples and variables of importance as per the rhizosphere conditions of rice varieties. R package was used to run the random forest models such as “randomForest,” “plyr,” “rfutilities” and “caret.” Univariate analysis was used to identify differentially abundant significant archaebacterial genera, whereas Lefse analysis was done to predict the important rhizobiome KEGG orthologues associated with rice varieties. For conducting univariate analysis and Lefse analysis MicrobiomeAnalyst pipeline was used (Dhariwal et al., 2017).

### Identification of microbiome network in rice rhizosphere

To elucidate microbe-microbe associations in the rhizosphere of rice and various drivers of ecological functions, co-occurrence networks based on the prevalence of the OTUs were identified. MetagenoNets pipeline was used as a guideline for various steps in this analysis (Nagpal et al., 2020). Firstly, the top 100 biologically relevant bacterial OTUs were identified through a random forest machine learning approach. Then these 100 bacterial OTUs and 24 archaebacterial OTUs were used for the prediction of microbial co-occurrence networks. The settings used for network predictions were:

- Algorithm: Pearson Correlation.
- Value of *p*: ≤ 0.05.
- Iterations: 500.
- Correlation cut-off: Based on Critical-R (For creating edges in the network).

Network topology including nodes, edges, diameter, density, and average degrees were calculated. For the identification of keystone species, nodes with the highest betweenness centrality values were extracted. Upon completion of this analysis 10 cultivars exhibited variations in microbial co-occurrence networks and functional guilds, which were discussed as network scenarios.

### Statistical analysis and data visualization

The calculation of PCA and multivariate analysis of the microbiome data were done using STAMP software, applying the Tukey–Kramer *post hoc* test and Benjamin-Hochberg FDR corrections (Parks et al., 2014). Box plot was drawn using the ggplot2 R package and heatmaps were drawn using the Clustvis tool (Metsalu and Vilo, 2015). The CIRCOS plot was drawn using the CIRCOS table viewer tool (Krzywinski et al., 2009).

### Data submission

The sequence data of this study has been submitted to NCBI, under BioProject accession number PRJNA790010.

## Results and discussion

### Plant variety influences on its root microbiome assemblage

**Figure 1:**
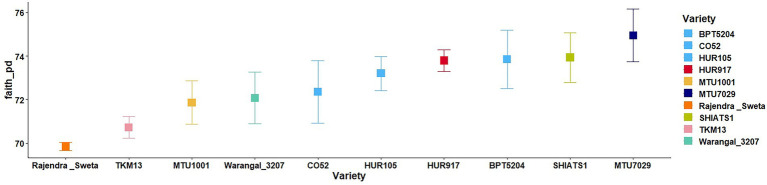
Variations in α-diversity indices of the rhizo-microbiome of rice varieties based on faith phylogenetic diversity. The significance of the diversity indices as per the rice varieties’ rhizosphere condition was determined based on the Krusal Walis test.

**Figure 2:**
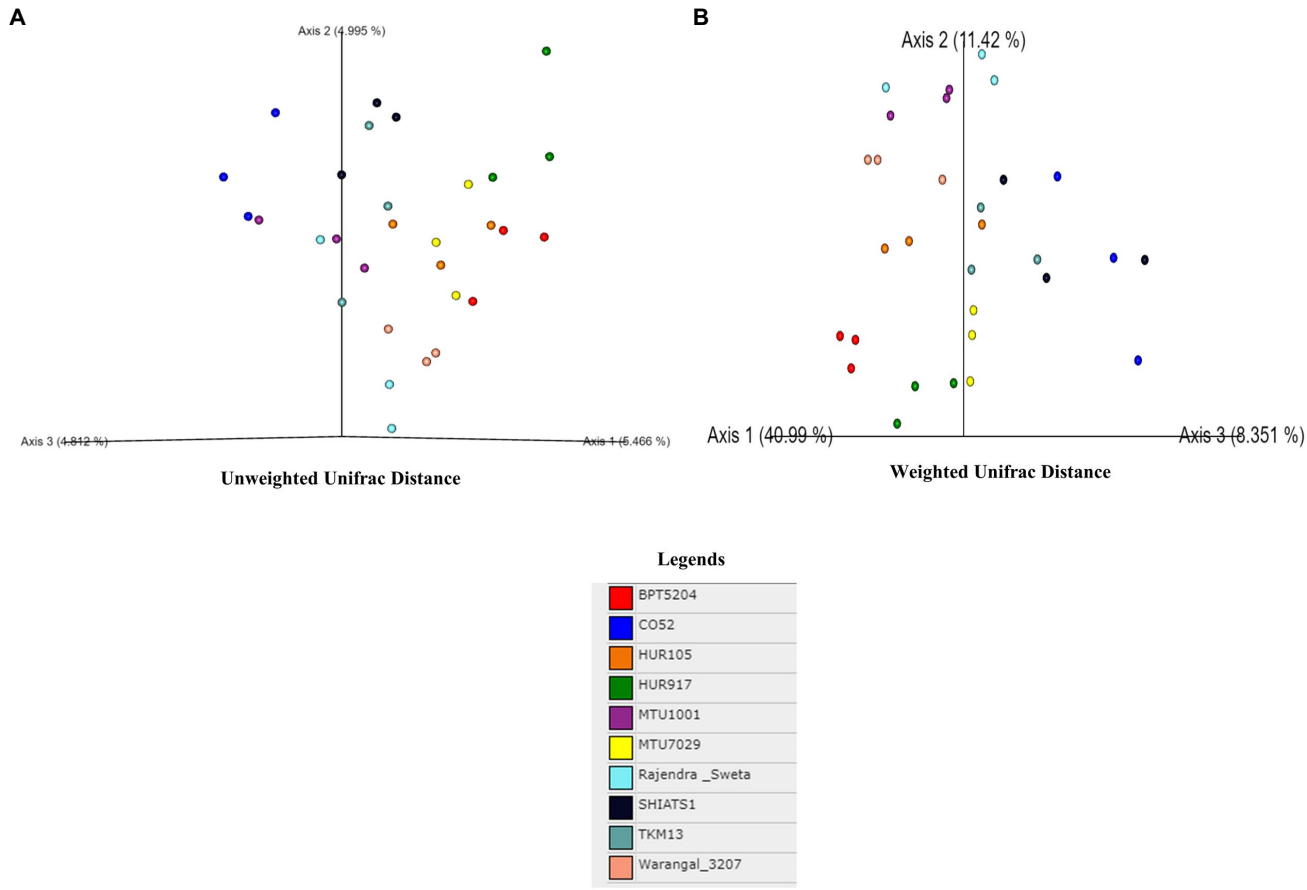
Effect of rice varieties on β-diversity of associated rhizo-microbiome as revealed by un-weighted **(A)** and weighted unifrac distances **(B)**.

**Figure 3:**
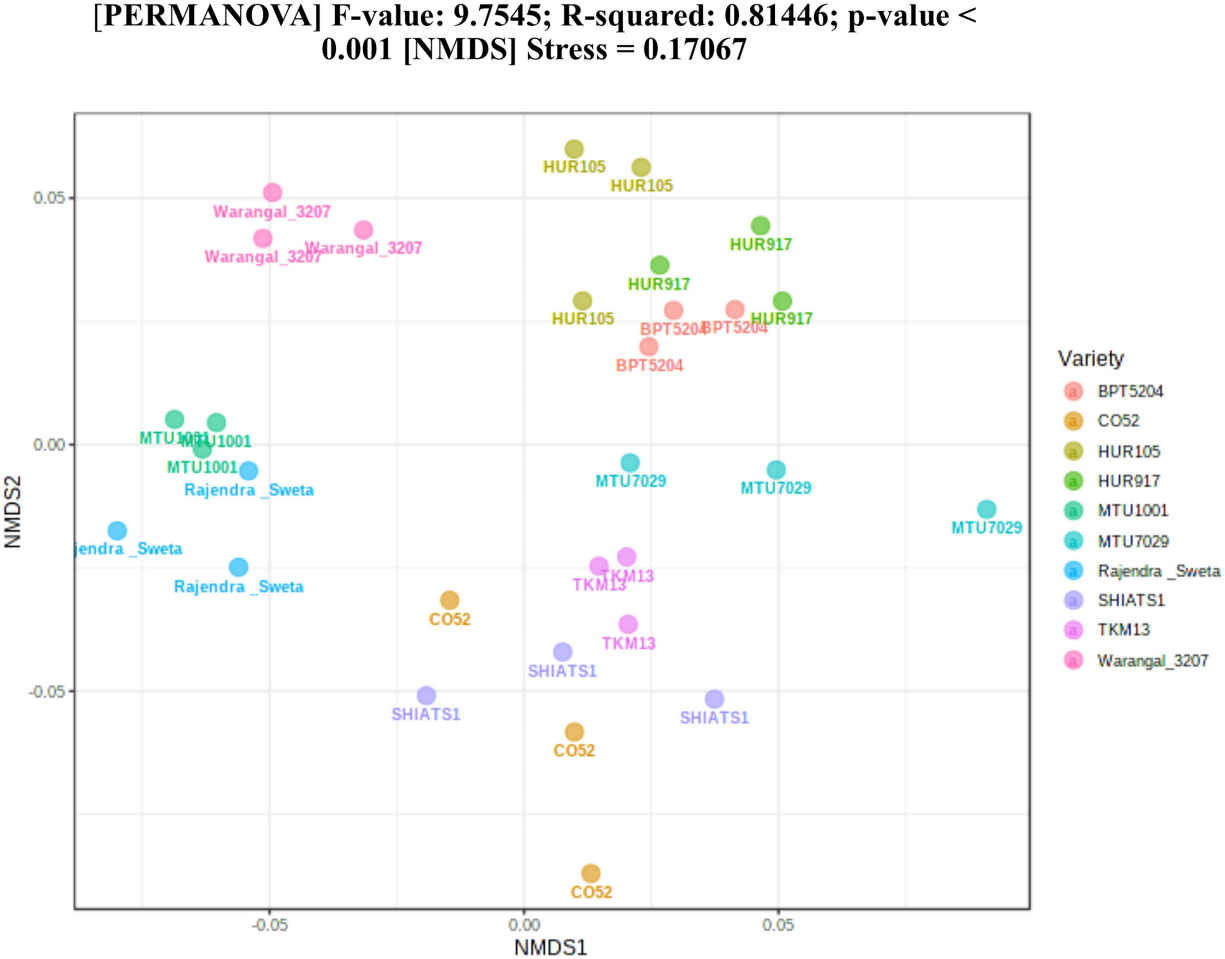
Estimation of rank based β-diversity based on NMDS-PERMANOVA to show statistical significance of the effect of rice varieties on the assemblage of rhizo-microbiome (*p* < 0.001).

A total of 30 rhizosphere soil samples, from 10 *indica* rice varieties, were collected. Their eDNA was isolated and sequenced, generating more than 18 million reads, with an average of 5 million reads per sample. The *de novo* sequence quality filtering and error corrections by deblur resulted in about 55,000 to 60,000 ASVs or microbial features (Supplementary Table S1). The faith_pd of rhizobiomes, an indicator of α-diversity, ranged from 69.85 to 74.95. Among the 10 *indica* varieties, phylogenetic diversity was highest for MTU7029 (faith_pd = 74.95 ± 2.08) and least for Rajender Sweta (faith_pd = 69.85 ± 0.343) (Supplementary Table S2; Figure 1). Statistical difference in the root-associated bacterial communities of the rice varieties was estimated by PERMANOVA, based on non-metric multi-dimensional scaling ordination (NMDS) as well as unweighted and weighted Unifrac distances. Based on phylogenetic relatedness of root-associated bacterial communities, both Unifrac distances revealed that rice varieties had a strong influence in recruiting their microbiome (Figure 2) and all the cluster groups had developed a unique microbiome related to their host. The rank-based β-diversity, based on NMDS ordination, also revealed that root microbiome assemblages of rice varieties are strongly linked to their hosts. Distinct clusters of rice varieties were observed along both axes, indicating the uniqueness of the microbial niches (Figure 3). The rhizosphere is a region of the root that acts as a highly transiently active zone of microbial successions and plant microbiome assemblage (Hartmann et al., 2008). It also contains root secretions having essential chemi-signals and nutrition, which attract diverse soil microorganisms to establish a root symbiotic microbial community (Ofaim et al., 2017). Previous studies have indicated that root exudates composition and the microbial community getting influenced, are dependent on the host’s variety and prevailing environmental cues (Li et al., 2018; Chen et al., 2019; Imchen et al., 2019). Results of the present study also reflected those significant variations obtained in α and β-diversities of *indica* rice microbiome assemblages are host induced and thus, strongly vary according to the host variety.

### Rhizospheric microbiome changes across rice varieties at phylum level

Taxonomic annotation of 16S rRNA reads revealed that 44 bacterial phyla were distributed across all the 10 rice rhizospheric groups. Statistically, the mean relative abundances of 33 bacterial phyla were found to be significant (Supplementary Data File S1). Among these, the most abundant bacterial phylum included *Proteobacteria*, *Chloroflexi*, *Bacterioidetes*, *Candidatus Phylum OD1 or Parcubacteria*, *Acidobacteria*, *Verrucomicrobia*, *Firmicutes*, and *Gemmatimonadetes* (Tables 1, 2). The mean relative abundance of *Proteobacteria* was found to be highest in rhizobiome of rice variety MTU1001 (22.42 ± 0.94%) and lowest in BPT5204 (17.37 ± 0.81%). Looking at the mean relative abundance of *Chloroflexi*, the rhizobiome of CO52 harbored the lowest population of the phylum (9.21 ± 0.41%), whereas the highest population was recorded for BPT5204 (14.21 ± 0.24%). The distribution of *Candidatus Phylum OD1* ranged from 4.8 to 7.4% in the rhizobiomes, highest was recorded for MTU7029 (7.42 ± 0.71%). Members of phylum *Acidobacteria*, *Verrucomicrobia*, and *Firmicutes* were evenly distributed in all the rhizobiome samples.

**Table 1:** Mean relative abundance (%) of top 20 bacterial phylum associated with rhizo-microbiome of the rice varieties.

Phylum	Mean relative abundance (%)									
	BPT5204	CO52	HUR105	HUR917	MTU1001	MTU7029	Rajender Sweta	SHIATS1	TKM13	Warangal3207
Proteobacteria^**^	17.37 ± 0.81	19.23 ± 0.92	18.32 ± 0.94	19.4 ± 0.42	22.42 ± 0.94	17.63 ± 0.26	22.4 ± 0.75	17.69 ± 1.09	22.02 ± 0.42	21.47 ± 0.81
Chloroflexi^**^	14.21 ± 0.24	9.21 ± 0.41	13.63 ± 0.46	13.52 ± 0.05	11.23 ± 0.53	11.58 ± 0.21	10.77 ± 0.96	12.92 ± 0.95	10.67 ± 0.33	10.71 ± 0.35
Bacteroidetes^**^	6.55 ± 0.17	7.52 ± 0.44	6.24 ± 0.18	7.14 ± 0.09	5.16 ± 0.25	6.59 ± 0.32	4.1 ± 0.14	7.22 ± 0.76	7.16 ± 0.24	4.3 ± 0.10
OD1^**^	6.43 ± 0.33	6.73 ± 0.21	6.7 ± 0.25	6.72 ± 0.37	4.88 ± 0.29	7.42 ± 0.71	5.73 ± 0.26	7.05 ± 0.44	5.79 ± 0.39	5.16 ± 0.21
Acidobacteria^**^	5.01 ± 0.16	3.41 ± 0.06	4.27 ± 0.16	3.95 ± 0.02	3.41 ± 0.16	4.05 ± 0.19	4.02 ± 0.50	3.19 ± 0.18	4.11 ± 0.38	4.54 ± 0.3
Verrucomicrobia^**^	3.43 ± 0.05	2.84 ± 0.11	3.17 ± 0.11	3.19 ± 0.1	2.12 ± 0.02	3.51 ± 0.08	2.42 ± 0.16	2.51 ± 0.25	3.48 ± 0.14	2.68 ± 0.25
Actinobacteria^**^	2.83 ± 0.19	2.44 ± 0.18	2.25 ± 0.11	2.13 ± 0.15	3.89 ± 0.18	2.39 ± 0.23	3.87 ± 0.08	2.55 ± 0.17	2.68 ± 0.11	3.68 ± 0.24
Firmicutes^**^	1.95 ± 0.02	1.78 ± 0.51	2.76 ± 0.26	2.71 ± 0.53	4.69 ± 0.11	1.48 ± 0.23	3.29 ± 0.09	2.43 ± 0.33	1.47 ± 0.09	2.54 ± 0.23
Cyanobacteria^**^	0.78 ± 0.04	1.53 ± 0.16	0.73 ± 0.001	0.74 ± 0.06	0.92 ± 0.02	2.07 ± 0.07	1.05 ± 0.13	2.42 ± 0.07	1.1 ± 0.04	0.82 ± 0.04
Gemmatimonadetes^**^	1.15 ± 0.09	0.82 ± 0.01	0.94 ± 0.09	0.96 ± 0.05	1.06 ± 0.09	0.97 ± 0.07	1.18 ± 0.05	0.65 ± 0.04	1.06 ± 0.08	1.28 ± 0.03

Statistical significance of the relative abundance of the bacterial phylum was estimated by multiple group ANOVA with Benjamini-Hochberg FDR multiple test correction. The *p* values <0.05 were considered significant. Values indicates mean ± standard deviation; ** indicates *p* values<0.05.

**Table 2:** Global network property of rhizo-microbiome of rice varieties.

Topology	HUR917	MTU1001	BPT5204	Warangal3207	TKM13	HUR105	CO52	Rajender Sweta	MTU7029	Co522	TKM133	MTU10014
Nodes	68	68	68	68	68	68	68	68	68	68	68	68
Edges	1,065	979	941	928	912	898	764	734	724	764	912	979
Diameter	3	3	3	3	3	3	3	3	3	3	3	3
Density	0.454	0.417	0.401	0.395	0.388	0.382	0.325	0.313	0.308	0.325	0.388	0.417
Average degree	30.86	28.37	27.28	26.89	26.43	26.02	22.14	21.27	20.98	22.14	26.43	28.37

Previous work for elucidating the composition of the rice rhizosphere microbial community has highlighted the abundance of *Gemmatimonadetes*, *Proteobacteria*, and *Verrucomicrobia* in the rhizosphere of the Koral rice genotype (Breidenbach et al., 2016). In many of the studies, the abundance of *Proteobacteria*, *Firmicutes*, *Actinobacteria*, and *Acidobacteria* was reported (Edwards et al., 2018; Ding et al., 2019; Imchen et al., 2019). The present findings were consistent with the past reports and similar results were observed at the phylum level.

### Important bacterial communities associated with *indica* rice rhizosphere

**Figure 4:**
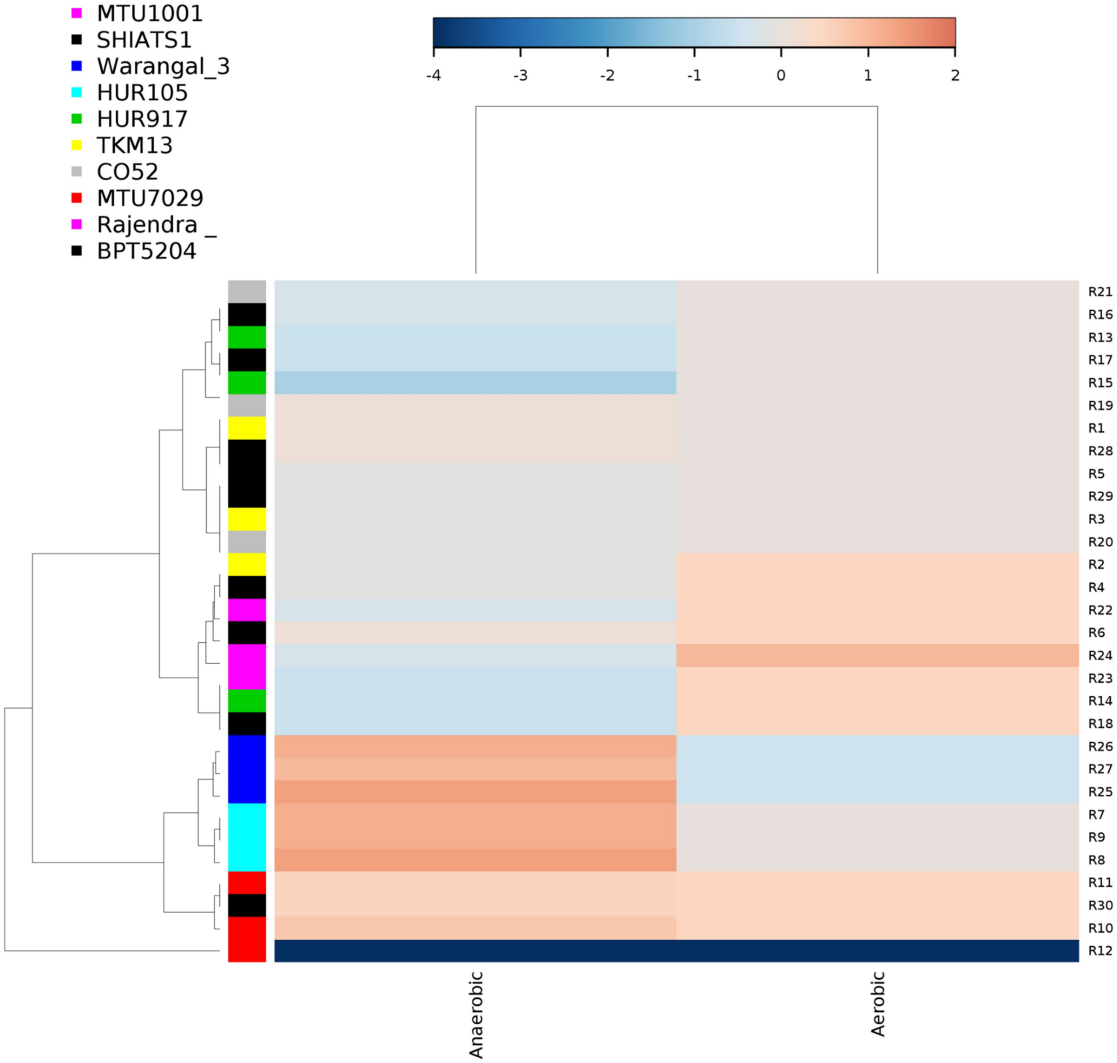
Distribution of aerobic and anaerobic bacterial flora in the rhizo-microbiome of rice varieties. For clustering Euclidean distances were estimated, the color gradient toward brown indicates higher abundance whereas lighter shades represent low abundance.

**Figure 5:**
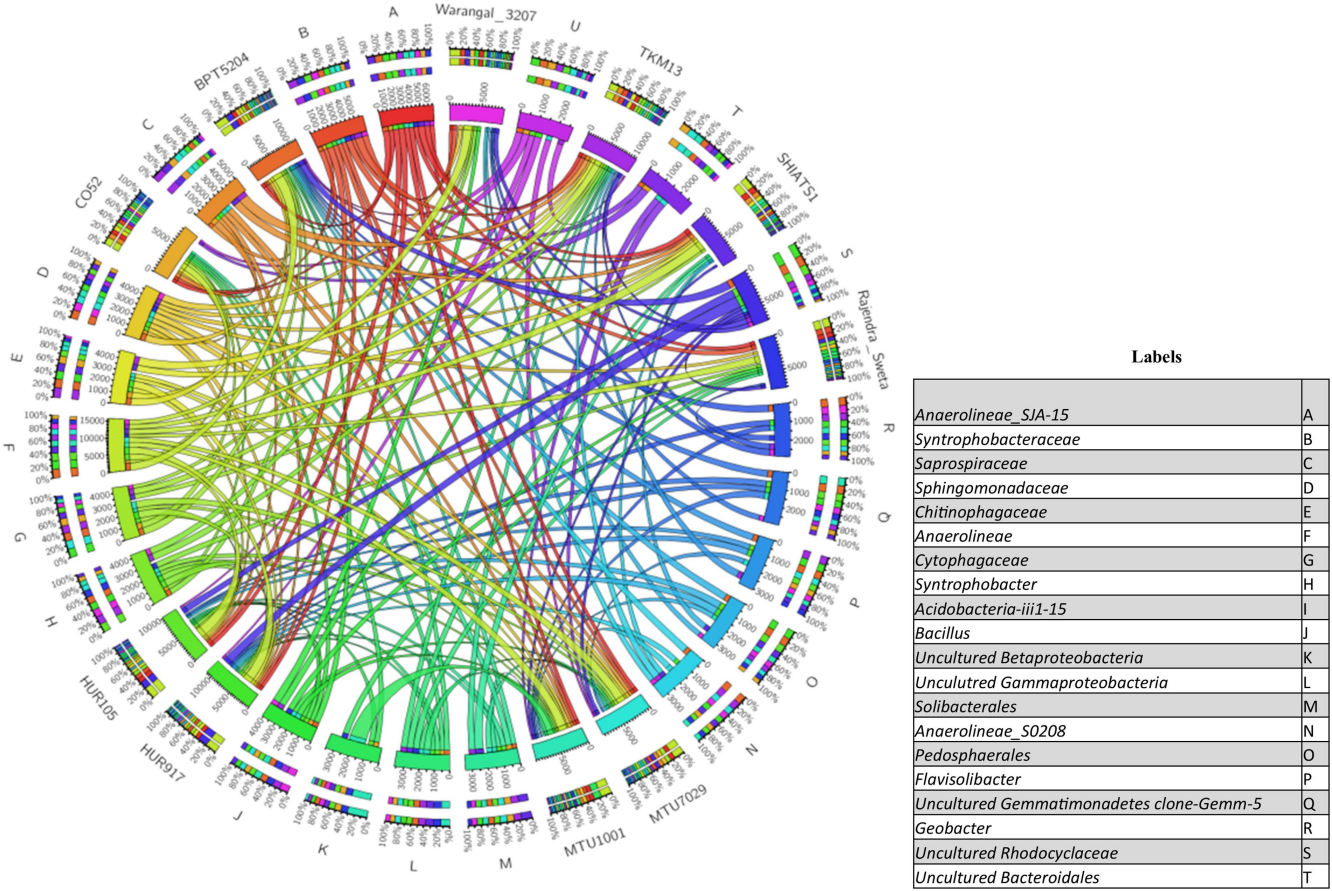
Circos plot showing the distribution of top 20 bacterial taxa (relative abundance >0.2%) associated with rhizo-microbiome of rice varieties.

There were inter-varietal differences at the genera level in rhizobiomes of rice varieties unlike the distributions of phyla. It was seen that due to the anoxic nature of submerged rice soil, there was a prevalence of facultative to obligate anaerobes in the presently studied rice rhizosphere (Figure 4). The submerged condition exhibited in the rice rhizosphere causes hypoxia or anoxia which leads to the enrichment of anaerobic bacterial communities (Liesack et al., 2000). The distribution of the top 20 bacterial taxa (above 0.2% mean relative abundance) among the rice rhizospheres, revealed that bacterial genera belonging to phyla-class *Anerolinea*, *Proteobacteria*, *Alphaproteobacteria*, *Betaproteobacteria*, *Bacteroidetes*, *Acidobacteria*, *Verrucomicorbia* and *Firmicutes* (Supplementary Data File S2; Figure 5) were the most dominant genera. Compositionally, unclassified *Anaerolineae* was most predominant phyla in the rhizosphere of all the 10 rice cultivars (2.36 to 4.52%), whereas even distribution of bacterial genus *Anerolinea clone SJA-15* (0.99 to 1.24%), *Anaerolineae_S0208* (0.41–0.75%), *Syntrophobacteraceae* (0.78 to 1.54%), *Sphingomonadaceae* (0.54–1.04%), *Chitinophagaceae* (0.73 to 1.15%), *Flavisolibacter* (0.35–0.62%) and *Pedosphaerales* (0.92 to 1.15%) was observed. The δ-proteobacterial taxa, iron-reducing bacterial genera *Geobacter* was found enriched in the rhizosphere of HUR917 (2.74 ± 0.17%), whereas propionate oxidizing bacterium *Syntrophobacter* was found to be highest in HUR105 (1.02 ± 0.07%). β-proteobacterial taxa, uncultured *Rhodocyclaceae* known for its bioremediation potential, was having highest representation in the rhizosphere of CO52 (0.73 ± 0.02%), whereas uncultured *β-proteobacteria* was most prominent in Rajendra Sweta (0.99 ± 0.1%). Uncultured *γ-proteobacteria* was found to be enriched in SHIATS-1 rhizosphere (0.90 ± 0.08%). *Acidobacterial* taxa *Solibacterales* (0.42–0.73%) and *Acidobateria*-iii1-15 (0.46–0.97%) were well distributed among the rice rhizobiomes. Uncultured *Gemmatomonadetes clone*, *Gemm-5*, known for its abiotic stress ameliorating capabilities was found to be predominant in Warangal 3,207 rhizosphere. *Bacteriodetes* taxa, *Sapropspiracea*, *Cytophagaceae*, and *Flavisolibacter*, were found to be enriched in rhizospheres of TKM13 (1.44 ± 0.02%) and MTU7029 (1.06 ± 0.11%), respectively, whereas uncultured *Bacteroidetes* was found to be enriched in the rhizosphere of CO52 (0.67 ± 0.04%). These bacterial genera are the potential source of a group of enzymes for carbohydrate metabolism. Taxa belonging to *Bacillus* was found to be highest in MTU1001 (1.76 ± 0.06%) rhizospheres. This indicates that the rice cultivars can tailor their rhizosphere microbiome according to their requirement.

**Figure 6:**
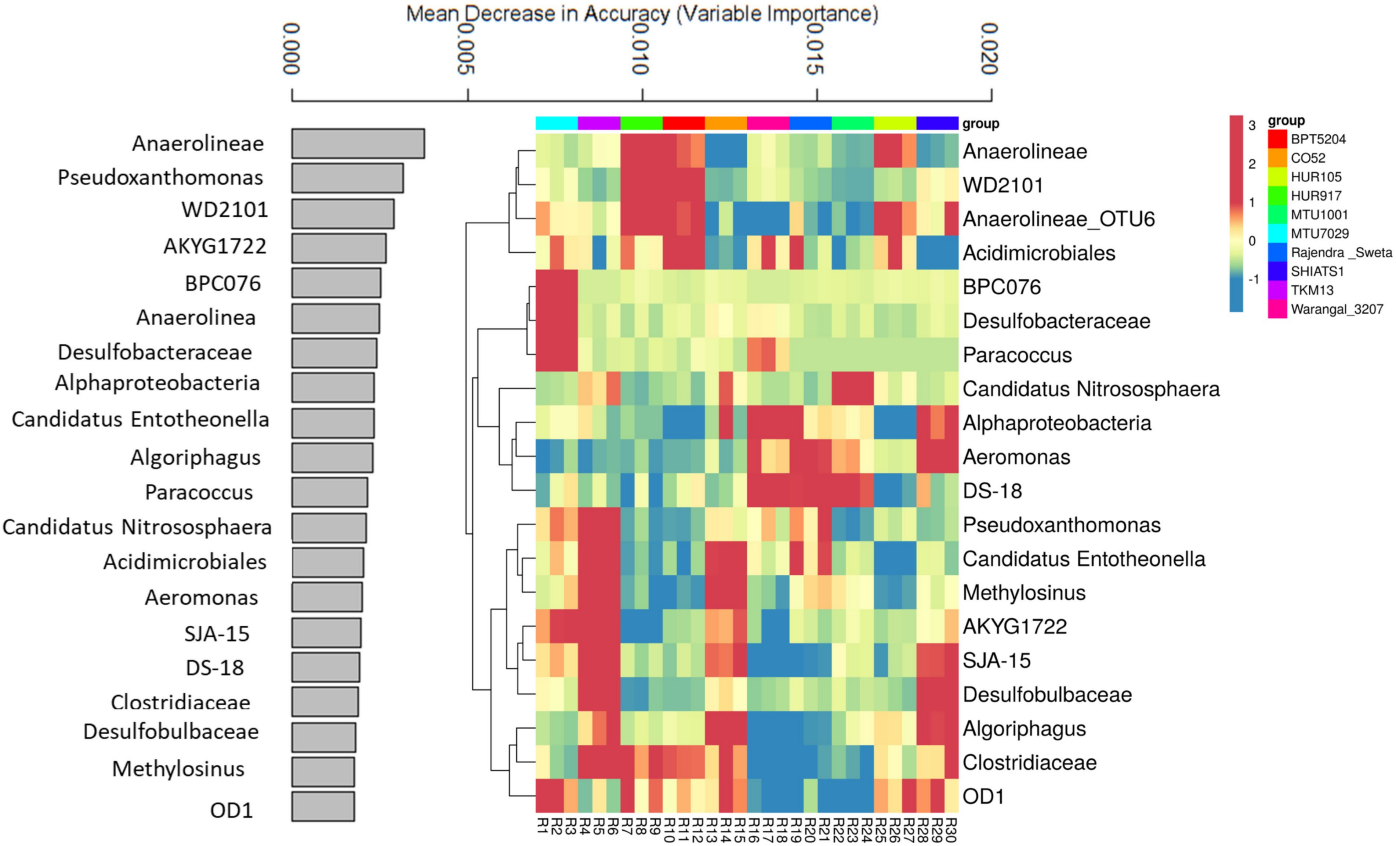
Random forest machine learning approach to identify important bioindicator bacterial taxa associated with rhizo-microbiome of rice varieties. The bar plot was drawn for the top 20 important bacterial taxa identified by the random forest machine learning approach, the values indicate mean decrease accuracy, along with heatmap was drawn to show the relative distribution of these bacterial taxa in the rice variety rhizo-microbiome, color gradient toward brown indicates high abundance whereas lighter shades represent low abundance, clustering was done based on Euclidean distances.

A random forest machine learning approach was used to predict key bioindicator taxa among rice rhizospheres. This helped in identifying niche-specific key bacterial genera associated with rhizobiomes of non-aromatic *indica* rice cultivars. The prediction model, utilizing bacterial OTUs predicted with 86.6% accuracy. The data for the top 20 key bioindicators have been discussed (Figure 6). Bacterial taxa *Anaerolineae* was predicted as a key bioindicator of rhizospheres associated with rice varieties HUR917, BPT5204, and HUR105. Uncultured bacterial clone *WD2101* was the key feature of HUR917 and BPT5204 rhizospheres. The bacterial genus *Anaerolineae_OTU6* was strongly linked to rhizosphere conditions of HUR917, BPT5204, HUR105, SHIATS1, and MTU7029. *Acidimicrobiales* was having niche specificity for rhizospheres of MTU7029, HUR917, BPT5204, Warangal_3,207, Rajender Sweta, and HUR105. Uncultured bacterial clones BPC076 and *Desulfobacteraceae* were key features of MTU7029 rhizobiomes, whereas *Paracoccus* was a bioindicator for MTU7029 and CO52. Ammonia oxidizing genus *Candidatus Nitrosphaera* was the key feature of rhizospheres of TKM13, CO52, and MTU1001. *Alphaproteobacteria* and *Aeromonas* emerged as the key feature of rice varieties Warangal_3,207, Rajender Sweta and SHIATS1. The uncultured bacterial clone was specifically associated with rhizospheres of Warangal_3,207, Rajender Sweta, and MTU1001. *Pseudoxanthomonas* was the prominent feature of rice rhizospheres of MTU7029, TKM13, and Warangal 3,207. Niche specificity of *Candidatus Entotheonella* and *Methylosinus* was predicted for rhizospheres of TKM13 and CO52. *Desulfobulbaceae* was predicted as a bioindicator for rhizobiomes of TKM13 and SHIATS1. The abundance of *Algoriphagus* and *Clostridiaceae* was marked as an important feature of rhizobiomes of CO52 and SHIATS1. The presence of *OD1* was identified as a key biological indicator of MTU7029, HUR917, CO52, HUR105, and SHIATS1 rhizospheres.

Due to the anoxic nature of rice rhizosphere, most of the niche-specific bacterial taxa were metabolically fermentative to organic acid utilization capability, showing dissimilatory sulphur and nitrogen reduction, hydrogen evolution, chemoorganotrophic to autotrophic bacteria, etc. The abundance of *Chloroflexi* class *Anearolinea*, which thrives on organic acids might be due to the anoxic degradation of polysaccharides. Earlier, the genus was well reported from anaerobic digesters, but its presence in the rice rhizosphere can be no exception due to low redox potential and high organic matter in the niche (Xia et al., 2016; Edwards et al., 2018). Well-distributed populations of dissimilatory sulphate reducing taxa, such as *Desulfobacteraceae*, and *Desulfobulbaceae* were showing niche specificity to rice rhizosphere. This taxon has been found to switch from sulfate to nitrate respiration as an alternative respiratory metabolism under an abundance of nitrate (Wörner et al., 2016; Zecchin et al., 2021). Dissimilatory nitrate reduction to ammonium (DNRA) is a characteristic of genera *Paracocccus* and *Pseudoxanthomonas*, which are reported from soil and groundwater sediments, and rhizosphere of corn, cotton, soya, and millet (Mohapatra et al., 2018; Pan et al., 2020). The abundance of DNRA activity in the rice rhizosphere is advantageous for transforming loss-prone nitrate ions to soil retainable ammonium ions (Tylova-Munzarova et al., 2005). Apart from DNRA activity, *Pseudoxanthomonas* have been reported to be involved in the reductive transformation of toxic arsenic, thus providing avenues for utilization of the genera for bioremediation of metalloids (Mohapatra et al., 2018). Rice rhizosphere of heavy metal contaminated soil has reported the presence of *Acidimicrobiales* (Huaidong et al., 2017). *Acidimicrobiales*, a photoheterotroph, is generally isolated from the deep photic zone of the marine environment. They assimilate C_2_ compounds using the ethyl-malonyl coenzyme A pathway (Chen et al., 2019). The growth condition of the bacterium highly suits the flooded condition of the rice rhizosphere. *Aeromonas* has been reported as the N-acyl homoserine lactone-mediated biofilm-forming bacterium from rice rhizoplane (Balasundararajan and Dananjeyan, 2019). The presence of a biofilm-forming rhizosphere community is involved in quorum sensing mediated colonization and antagonistic responses against the foreign pathogen (Pieterse et al., 2016). The rice rhizospheres were also harboring potential organic matter degraders *Algoriphagus* and *Clostridiaceae*, which have been reported as major players in carbon cycling (Gavande et al., 2021). Type II methanotrophic bacteria *Methylosinus* has been isolated from the rhizosphere of *Oryza sativa Nipponbare*, which not only oxidizes methane but also possesses a nitrogenase complex suggesting its role in maintaining carbon and nitrogen cycling in the root zone of rice (Bao et al., 2014). Environmental bacterial taxon with a large and distinct metabolic repertoire, *Candidatus Entotheonella* was reported from marine sponge *Theonella swinhoe* (Wilson et al., 2014). This is the first report of *Candidatus entotheonella* from the rhizosphere of rice. Metabolically they utilize oxalate and methanol as carbon sources, which are found abundantly in the rice rhizosphere. The taxa is a treasure trove for many important metabolites (Wilson et al., 2014).

### Predominant archaebacteria associated with *indica* rice rhizosphere

**Figure 7:**
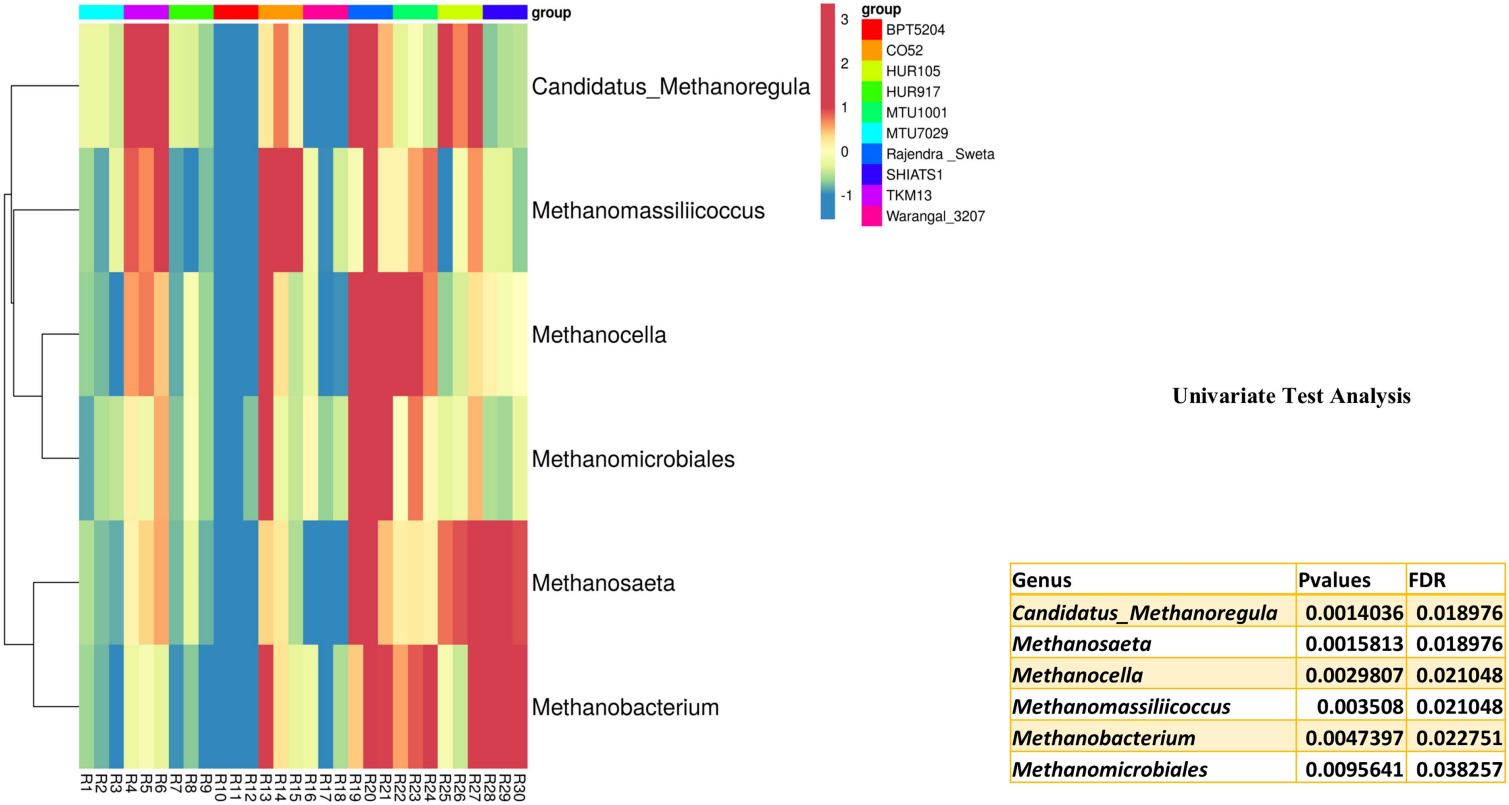
Univariate test analysis to identify important archaebacterial taxa associated with rhizo-microbiome of rice varieties, the heat map is drawn taking their relative abundance and clustering was done based on Euclidean distances, color gradient toward brown indicates high abundance, whereas lighter shades represent low abundance.

*Archaebacterial* OTUs were also widespread among the rice rhizospheres. These OTUs were classified under phylum *Euryarchaeota*, *Parvarchaeota*, and *Crenarchaeota*. Univariate analysis was performed to predict differentially abundant archaebacterial taxa of the rice rhizospheres. Six archaebacterial genera *viz*., *Candidatus Methanoregula* (*p* = 0.001), *Methanosaeta* (*p* = 0.002), *Methanocella* (*p* = 0.003), *Methanomassiliicoccus* (*p* = 0.004), *Methanobacterium* (*p* = 0.005) and *Methanomicrobiales* (*p* = 0.01) were found to be differentially abundant among the rice rhizospheres under study (Figure 7). All the differentially abundant taxa were found to be belonging to *Euryarchaeota*, capable of hydrogenotrophic methanogenesis (Berghuis et al., 2019).

Among the anthropogenic sources of methane emission, paddy cultivation accounts for 25 to 100 teragrams of CH_4_ per year (Conrad, 2020). Liechty et al. (2020) have characterized rice cultivars based on the rate of methane emission. Through deep sequencing approaches, they found that syntropic methanogenesis was more prevalent in high emission rice cultivars as compared to low emission cultivars. A high abundance of hydrogenotrophic methanogen throughout the growth period of rice indicates its niche specificity for rice roots (Imchen et al., 2019). Ammonia oxidizing *Crenarchaeota* genera *Candidatus Nitrosphaera* has also been reported as the predominant ammonia-oxidizing archaea from the rice roots (Imchen et al., 2019).

### Unveiling the microbiome networks of rice rhizosphere

Root microbiome assemblages play a key role in the sustenance of plant health and productivity. Community metagenomics is the only tool that can be able to decipher how the individual taxa within the microbiome are distributed in the host plant. However, the underlying mechanism for the interaction of microbe-microbe at the trophic level is not revealed. Hence, co-abundance network analysis was performed by taking into account a 100 bioindicator bacterial OTUs predicted by a random forest machine learning algorithm with 24 archaebacterial OTUs. We have generated 10 different co-occurrence microbiome network scenarios specific to microbiomes of rice varieties under study (Supplementary Figure S1; Supplementary Table S2). Global network properties such, as the average number of edges per node (average degree) were calculated to reveal network topology. To define the functionality/ecological role of the microbiome networks we have also predicted the keystone species based on the betweenness centrality values of the nodes.

### Scenario 1: Syntropic nitrogen and carbon cycling

Co-abundance network analysis for rhizobiome of HUR917 yielded 1,065 significant correlations (Supplementary Figures S1A, S2; Supplementary Table S3). The network’s keystone species was *Crenarchaeota* (ArcOTU_2), a chemoautotrophic ammonia oxidizer (Martin-Cuadrado et al., 2008). It was found that keystone species have shown a positive significant correlation with complex organic matter degrading bacterial taxa *Clostridium* (OTU_60) and *Saprospiraceae* (OTU_10), along with nitrogen-fixing taxa *Syntrophobacteraceae* (OTU_55), *Rhodospirillales* (OTU_49) (McIlroy and Nielsen, 2014; Liu and Conrad, 2017). A strong positive correlation of ammonia oxidizers with nitrogen-fixing taxa has been reported in ecosystems of pastures and crops in Amazonia, Brazil (Lammel et al., 2015).

### Scenario 2: Multispecies synergistic biofilms conferring biotic stress amelioration

The Microbiome network scenario of MTU1001 had 979 significant correlations (Supplementary Figures S1B, S3; Supplementary Table S4). Rhizobiome of MTU1001 has *Aeromonas* (OTU_87) as the keystone species. Strains of *Aeromonas* have been reported to form multispecies synergistic biofilms through the type 1 autoinducer quorum sensing mechanism (Li et al., 2008). There is a strong possibility that positive interactions with *Bacteroidetes* (OTU_50) and *Rhizobiales* (OTU_37) might be involved in forming biofilms and regulation of chitinase production (Ochiai et al., 2013). Thus, it can be concluded that the bio-filmed microbiome network of MTU1001 might be playing a role in host defense against biotic stresses.

### Scenario 3: Diel cycling to synchronize with host circadian rhythm

There were 941 significant correlations in the microbiome network of BPT5204 (Supplementary Figures S1C, S4; Supplementary Table S5). Keystone species was predicted as *Gaiellaceae* (OTU_124). Genomic studies revealed that members of the *Gaiellaceae* family have Calvin–Benson–Bassham (CBB) cycle with a type I RuBisCO and show diel cycling (Severino et al., 2019). Keystone OTU was establishing positive interactions with other anoxic chemoorganotrophic bacterial taxa *Rhodospirillales* (OTU_49) and *Anaerolineae* (OTU_6). These interactions indicate that carbon metabolism in this microbiome was regulated preferably by chemoautotrophs showing diel cycling (Alcamán-Arias et al., 2018). Further, diel cycling also has an added advantage for the rhizospheric community to synchronize their abundances as per the circadian rhythm of the host plant (Staley et al., 2017).

### Scenario 4: Syntrophic methanogenesis

The Microbiome network of rice variety Warangal 3,207 had 928 significant correlations (Supplementary Figures S1D, S5; Supplementary Table S6). *Sinobacteraceae*, (OTU_121) was discovered as keystone species of the network. A significant positive correlation was found with *Candidatus* genera JG30- KF-CM45 (OTU_25) of *Chloroflexi* and *Luteolibacter* (OTU_84). Metabolically, the consortia might be involved in the cycling of nitrogen in the rhizosphere as *Sinobacteraceae* carry out ammonia oxidation (Bäumgen et al., 2021), whereas *Candidatus* genera JG30- KF-CM45 possess DNRA activity and the simple sugars are being provided through polysaccharide degradation by *Luteolibacter*. The consortia were showing competitive interaction with *Methanosaeta* (ArcOTU_12). Energetically, the requisite amount of hydrogen and organic acid gets consumed in supporting DNRA activity and ammonia oxidation, thus slowing down the process of methanogenesis.

### Scenario 5: Syntrophic methanogenesis

Rhizobiome network analysis of TKM13 yielded 912 significant correlations (Supplementary Figures S1E, S6; Supplementary Table S7). Polysaccharide degrader *Luteolibacter* (OTU_86) was found as keystone species (Cardman et al., 2014). Syntrophic association was observed with fermentative polysaccharide degrader *Clostridiaceae* (OTU_21), biofilm-forming genera *Aeromonas* (OTU_87), forming an organic matter degrading guild. The organic matter degrading guild was also positively correlated with hydrogenotrophic methanogens like *Methanosaeta* (ArcOTU_12), *Methanobacterium* (ArcOTU_6), etc. Anoxic degradation of organic matter results in the production of organic acids such as acetates, lactates, and formic acids. These organic acids are further utilized by hydrogenotrophic methanogens as substrates for methane formation (Shiratori et al., 2006).

### Scenario 6: Syntrophic methanogenesis

The microbial network of HUR105 yielded 898 significant correlations (Supplementary Figures S1F, S7; Supplementary Table S8). Cellulose degrading taxa *Algoripahagus* (OTU_95) was identified as the keystone species (Deng and Wang, 2017). Propionate oxidizing bacterial family *Syntrophobacteraceae* (OTU_55) and *Methanocella* (ArcOTU_7) have shown a syntrophic association with *Algoripahagus*. During this interaction, organic acids like propionic acid produced during the degradation of organic matter is utilized by *Syntrophobacteraceae* resulting in the generation of acetate and hydrogen (Liu and Conrad, 2017). These compounds are further utilized by *Methanocella* symbiotically to produce methane (Sakai et al., 2014).

### Scenario 7: Syntrophic methanogenesis

Co-abundance microbial network of CO52 yielded 764 significant correlations (Supplementary Figures S1G, S8; Supplementary Table S9). Keystone species was predicted as *Gaiellaceae* (OTU_124). As described above members of the *Gaiellaceae* family have Calvin–Benson–Bassham (CBB) cycle with a type I RuBisCO and show diel cycling. Although the ecological significance of the taxa is unclear (M. et al., 2021). Strong positive correlation with chemoorganotrophic, *Anerolinea* (OUT_6, OTU_9), chemolithotrophic *Geobacter* (OTU_57), facultatively autotrophic hydrogen-oxidizing bacterium *Hydrogenophaga* (OTU_28) and acetogenic methanogen *Methanocella* (ArcOTU_7) was suggestive of carbon cycling guilds (Li et al., 2015; Liechty et al., 2020). The direct positive correlations of the bacterial OTUs with methanogen are suggestive of up-regulation of methanogenesis (Liechty et al., 2020).

### Scenario 8: Syntrophic methanogenesis

Microbial network analysis of rhizobiome of Rajender Sweta had 734 significant correlations (Supplementary Figures S1H, S9; Supplementary Table S10). The keystone species of the network was found as *Aeromonas* (OTU_87). Syntrophic association was seen with *Acidobacterial candidatus* family *Ellin 6*,*075* (OTU_58), *Anaerolinea* (OTU_6), *Candidatus Entotheonella* (OTU_185), *Bacteroidetes* (OTU_50), and *Methanosaeta* (ArcOTU_12). This particular network was syntrophically forming complex organic matter degradation guilds for the production of acetate and hydrogen and thus supplementing the substrate demand for methane formation (Wrighton et al., 2014; Eichorst et al., 2018). Further, the carbon cycling also favors ammonia oxidation due to the prevalence of *Candidatus Nitrososphaera* (ArcOTU_3) positively correlated node (Sauder et al., 2017).

### Scenario 9: Case of anaerobic methane oxidation

The microbial network architecture of MTU7029 revealed 724 significant correlations (Supplementary Figures S1I, S10; Supplementary Table S11). Hydrogenotrophic methanogen, *Methanomicrobiales* (ArcOTU_8) was found as the keystone species. Two Anaerobic polysaccharides degrading fermentative *Chloroflexi* OTU’s (OTU_154 and OUT_101), denitrifying α-proteobacteria (OTU_37) and propionate degrading β-proteobacteria (OTU_16) were forming a syntropic association with keystone species. Degradation of polysaccharides and propionic acids yielded substrate and electrons for sustaining methanogenesis. On the contrary, we found that the guild was performing the denitrification process which was earlier known to suppress methane formation, but it was indicated that in the presence of nitrate, the process of methanogenesis process gets reversed to anaerobic methane oxidation (Bulseco et al., 2019). Hence, instead of methanogen abundance, methane flux is getting reduced in this microbiome interaction scenario.

### Scenario 10: Syntrophic methanogenesis

The Microbiome network of SHIATS1 had 692 network correlations (Supplementary Figures S1J, S11; Supplementary Table S12). *Candidatus* taxa OPB54 (OTU_168) which co-produces ethanol-hydrogen was the keystone species (Liu et al., 2016). Significant positive correlations were seen with other fermentative bacterial-archeal taxa such as *Geobacteraceae* (OTU_57), *Anaerolinea* (OTU_6), *Parvarchaea* (ArcOTU_23) along with hydrogenotrophic methanogen *Methanobacetrium* (ArcOTU_6) (Hug et al., 2013; Castelle and Banfield, 2018; Zhang et al., 2020). These archaeal bacterial consortia were acting as organic carbon cycling guilds, and organic acids and direct interspecies electron transfer were also being mediated to facilitate methanogenesis.

**Figure 8:**
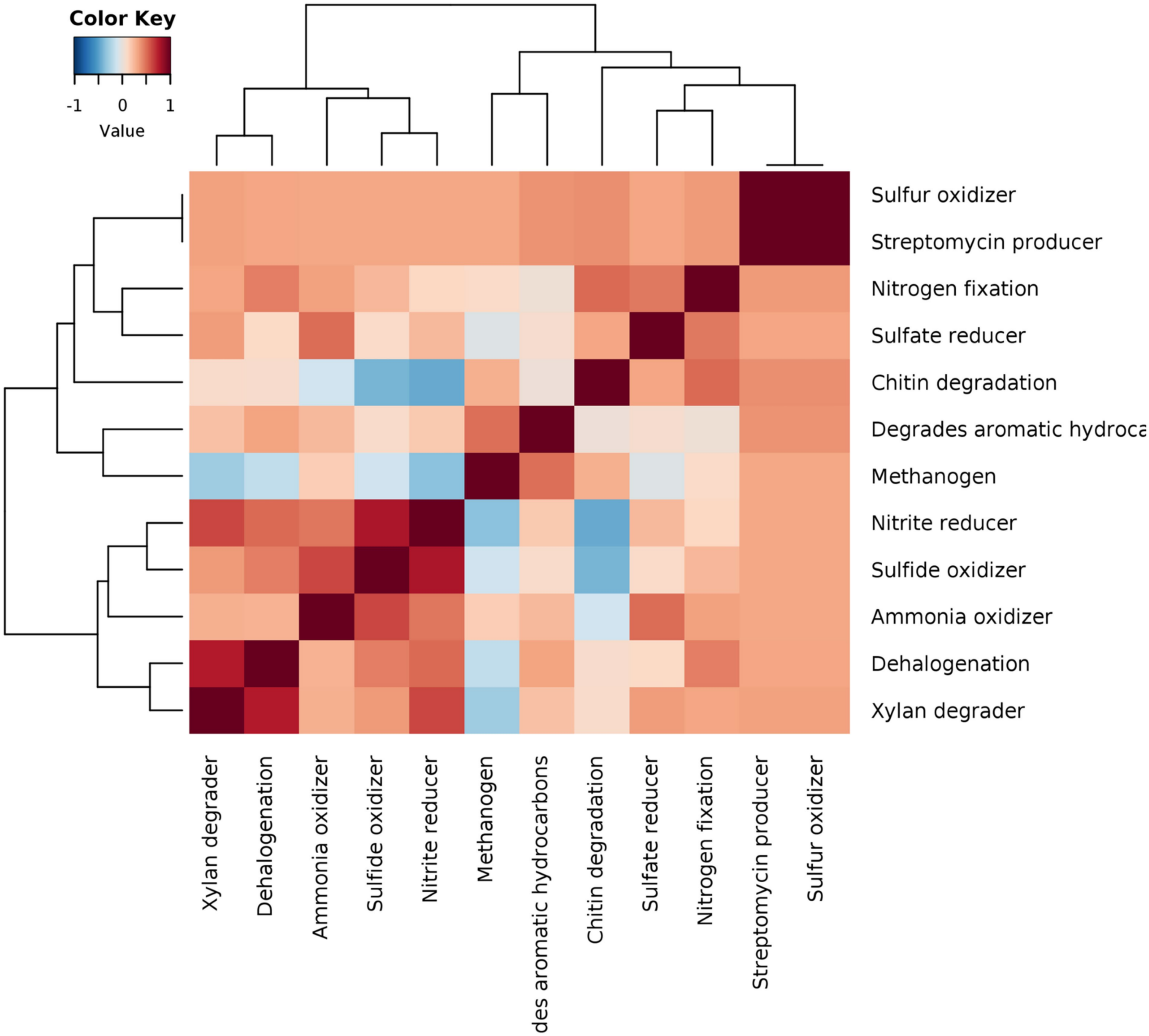
Pearson correlation based heatmap showing the distribution of important soil ecological functions associated with rhizo-microbiome of rice varieties, color gradient toward brown represents high positive correlation and toward blue represents negative correlation.

To conclusively represent various microbiome network scenarios and their ecological significance in the rice rhizosphere, taxonomic to phenotype mapping was performed to infer ecological functions. The mapping revealed 12 ecological functions *viz*., streptomycin production, xylan degradation, degradation of aromatic hydrocarbon, sulfur oxidation, sulphite oxidizer, sulphate reduction, ammonia oxidation, dehalogenation, nitrogen fixation, nitrate reducer, methanogenesis, and chitin degradation. The correlations were drawn between the functions and it was observed that strong positive correlations can be observed in two clusters *viz*., xylan degrader vs. dehalogenation vs. ammonia oxidizer vs. sulphide oxidizer vs. nitrite reducer (Cluster 1) and chitin degrader vs. sulphate reducer and nitrogen fixation (cluster 2) (Figure 8). These functional interactions were well supported by our results on microbiome networks that play an important role in carbon, sulfur, and nitrogen cycling. Methanogens were found to show weak positive correlations with sulfate and nitrate reducers, which was in line with the fact that the addition of nitrate and sulfate to the environment reverses the process of methanogenesis and supports anaerobic methane oxidation.

### Prediction of important gene families associated with rice rhizobiome conferring plant growth promotional traits

**Figure 9:**
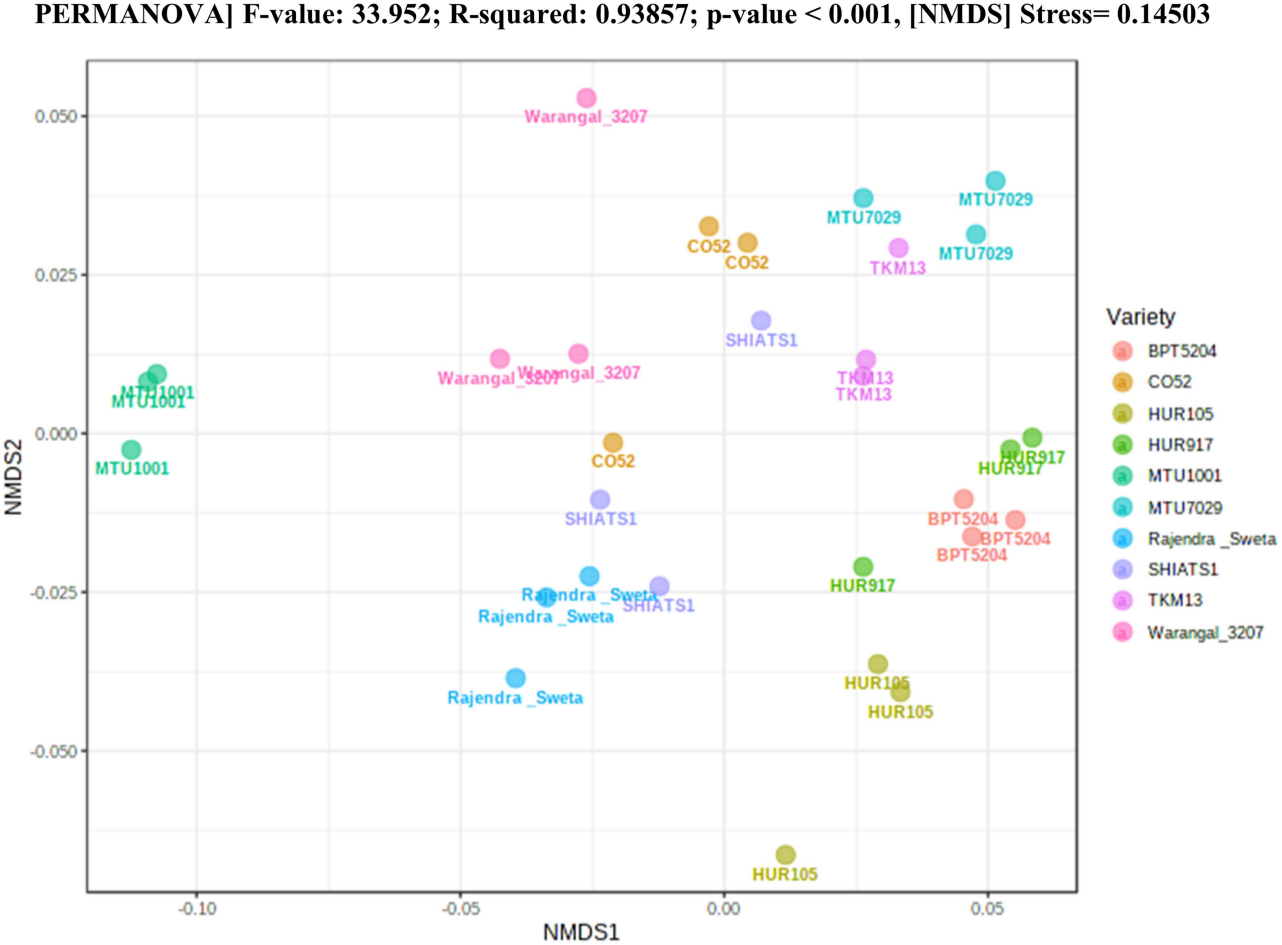
Principal coordinate analysis to reveal statistically significant differences in the association of various KEGG orthologues-based functions associated with rhizo-microbiome of rice varieties. To perform the clustering PERMANOVA based on NMDS was used.

PICRUSt2 was used to infer gene families associated with important metabolic pathways associated with rice rhizobiomes (Douglas et al., 2020). The tool enables accurate prediction of genomes based on the ASV’s data generated during bacterial profiling of the samples. PICRUSt2 works by placing the 16S rRNA gene sequences of the samples in a reference tree of 20,000 full length 16S rRNA gene sequences. Based on the phylogenetic placements of the ASV’s genome predictions are performed and metagenome is reconstructed.The pipeline predicted more than 7,000 KEGG orthologous (KO) associated with the rhizobiomes. As the rice rhizobiomes were harboring their bacterial community structure, it was also observed that significant differences in metabolic functions were observed among the rhizobiomes (Figure 9). The differential abundance of various gene families was inferred by performing Lefse analysis, and 50 differentially abundant KOs enriched in rhizobiomes were predicted (Supplementary Figures S12A,B).

The rhizosphere has been designated as the hub for many metabolite exchange networks and signaling channels with soil bacterial communities (Hermans et al., 2017; Sasse et al., 2018). Compositionally root exudates contain many metabolites like flavonoids, strigolactone, organic acids, ions, sugars, phenols, etc., many of them serve as an energy source and many as chemotactic signals to establish rhizobiome. Under the influence of these metabolite, selective overexpression of prokaryotic genes conferring nutrient acquisition, biological nitrogen fixation, environmental stress tolerance, colonization, rhizosphere competence and biotransformation of organic matter has been observed during the cross-talks (Matilla et al., 2007; Dong et al., 2019). The inferred functional predictions of rice rhizobiome suggest the abundance of many KO’s involved in rhizosphere competence of bacterial community. Differential abundance of ABC-transporter (K02004, K02003) suggests an increase in activity of uptake of organic acids, amino acids, sugars, phosphorus mobilization, and exchange of sulfur-containing molecules in root zones (Zerbs et al., 2017; Lidbury et al., 2019). Thus, there is a preferential increase in the colonization of bacterial communities having a preference for the root exudate metabolites. Since the rhizodeposits of paddy are carbonaceous and about 1.61–5.24 t ha^−1^ gets released in paddy fields (Wang et al., 2017). Correlating with the nature of rhizodeposits, the majority of the functional orthologous were annotated to carbon cycling and sequestration groups. Li et al. (2018) have found that compositional variations of the dissolved organic carbon in the paddy fields are the major drivers of carbon cycling pathways. As per our results and a previous study performed by Li et al. (2018) on the paddy field regarding methane metabolic pathway (K05299) it was found that they were non- acetoclastic and were *via* CO_2._ Further, it was also observed that an alternate source of electron donor other than H_2_ for methanogenesis was observed for instance presence of aldehyde: ferredoxin oxidoreductases (Aor, K03738) which catalyze the conversion of aldehyde to carboxylic acid and thereby supplying reduced ferredoxin (Beulig et al., 2019). Paddy ecosystem was also acting as an active carbon sequestering zone as many of the autotrophic carbon fixation pathways KOs was found abundant, such as 2-oxoglutarate/2-oxoacid ferredoxin oxidoreductase subunit alpha (K00174), 2-oxoglutarate ferredoxin oxidoreductase subunit gamma (reductive citric acid cycle, K00177) and transketolase (Calvin Benson Cycle, K00615). The abundance of these KO’s has been reported in *Gaiella* and *Desulfobacter* (Hügler et al., 2010; Severino et al., 2019), these genera were reported as keystone species and bioindicators in the present study. Methanotrophy has been found as one of the features of the paddy ecosystem (Van Bodegom et al., 2001). Particulate methane monooxygenase is the key enzyme involved in methane oxidation, the electron transfer in the whole process is mediated by NADH: Quinone Oxidoreductase, the abundance of KO’s NADH-Quinone oxidoreductase subunit N (K00343), NADH-Quinone oxidoreductase subunit M (K00342) and NADPH: quinone reductase (K00344) suggest their role in the regulation of methane monooxygenase (Cook and Shiemke, 2002). A KO involved in alkane biosynthesis was also discovered, the KO (K00257) was annotated to acyl-ACP dehydrogenase. This enzyme in combination with aldehyde decarboxylase converts intermediates of fatty acid to alkanes and alkenes, the pathway is prevalent in *Synechococcus* (Schirmer et al., 2010). The discovery of the alkane biosynthesis pathway in the rice rhizosphere presents an interesting avenue for researchable steps in the direction of biofuel production.

Looking for the distribution of nitrogen regulators, it was found that *NtrC* a phospho-relay sensor kinase (K02428), was prevalent in the rice rhizosphere. Biochemically it catalyzes the phosphorylation of histidine residue in response to changes in the environment. It has been reported that in *Rhodospirillum rubrum*, phosphorylated NtrC regulated the posttranslational regulation of nitrogenase activity (Zhang et al., 2005). Dissimilatory sulphate reduction has been the most important process of microbial respiration in reducing the environment. The membrane complex QrcABCD has been widespread in the sulphate-reducing bacteria *Desulfovibrio* and is involved in energy conservation during sulfate respiration (Venceslau et al., 2010; Zecchin et al., 2018). The QrcABCD complex is involved with the electron transfer subunit consisting of the DMSO reduction module consisting of dmsB (K0184) and dmsC (K00185) for sulphate reduction, which are found in the present study (Duarte et al., 2018). Sulphate permease (SulP, K03321), acts as a sulphate transporter during the dissimilatory sulphate reduction (Aguilar-Barajas et al., 2011). The overexpression of SulP was observed when there was a drop in sulphate concentration (Kharwar et al., 2021).

Biogeochemical cycling of metalloids, especially arsenic (As) has been gaining importance in the context of paddy cultivation. ArsR family transcriptional regulator (K03892), is a part of the arsenic sensing module. The ArsR represses arsenical resistance operon in the absence of As (III) (Slyemi and Bonnefoy, 2012). Complete phosphate uptake regulon comprising of two-component system response regulator of phosphate assimilation, PhoP (K07658) and phosphate transport system substrate-binding protein, PstS (K02040) was detected in the rhizosphere. Under phosphate limitation, PhoP binds with the promotor region and expresses the synthesis of PhoA Alkaline phosphatase which mineralizes the phosphate, and the inorganic phosphorous binds with PstS which is transported in the cell (Apel et al., 2007; Chhabra et al., 2013). Complete phosphate uptake regulon suggests its role in maintaining adequate phosphate levels in the rice rhizosphere.

Much environmental stress tolerance KO’s responsible for imparting tolerance against osmotic and heat stress were also predicted in the paddy rhizosphere. For instance, a two-component system atoC regulator for c Poly-Hydroxybutyrate biosynthesis (cPHB) (K07714) was detected. PHB biosynthesis imparts protection of non-halophilic bacteria under a hyper saline environment (Obruca et al., 2017). The presence of chaperone protein families such as HSP20 family protein (K13993), *DnaK* (K04043), and chaperones and folding catalysts (K03769) confers the role of rhizobiome in imparting heat tolerance to the host plant (Xue et al., 2019).

Regarding biotic stress, KO involved in biofilm formation was predicted. The Phenyl acetate CoA ligases (K01912) are involved in polysaccharide synthesis as a part of the biofilm formation pathway in the rice rhizosphere. The formation of biofilm in the rice rhizosphere is suggestive of its role in inducing antagonism against invasive microbiota thus conferring induction of resistance to the host plant. This KO has also been detected as part of the *nfs* cluster of *Pseudomonas* sp. P482 which confers biocontrol activity against plant pathogens (Krzyżanowska et al., 2021).

Chemotaxis is an important rhizobacterial competence characteristic, which helps the bacterial community to recognize the host and establish itself. Among the chemotactic responsive KO’s OmpR family protein (K02483) and flagellin protein (K01426) were detected. The nitrogen-fixing plant symbiont *Sinorhizobium meliloti* chemotaxis and motility are regulated by OmpR-like product Rem which exerts control over flagellin and chemotaxis genes (Rotter et al., 2006). Lrp/AsnC family transcriptional regulator (K03719) is a putative leucine-responsive regulator of the ACC deaminase gene (*acdS*) (Checcucci et al., 2017). The *acdS* gene has been an important feature of the rhizosphere bacterial genome, as the gene product 1-aminocyclopropane-1-carboxylate (ACC) deaminase cleaves plant-derived ACC, which is the precursor of the plant stress-hormone ethylene, thus delaying the stress-induced senescence in the host plant (Glick et al., 2007).

## Conclusion

The outcome of the work presented thereupon revealed that the rice root microbiome is greatly influenced by the cultivar types. Results obtained through high throughput *in silico* approaches based on machine learning, microbiome network predictions and metagenomic reconstruction has helped in predicting many new important bacterial taxa co-existing with each other in the rhizosphere zone of the rice plants. Their interplay was also regulating the C-N-P-S cycling, methane emission, and amelioration of abiotic and biotic stresses on the host genotype. The studies also revealed several keystone genera regulating the functional guilds of root microbiome but were still non-culturable, and to utilize them, novel approaches should be devised for *in vitro* culturing. The new technique of culturomics has been widely used for the discovery of new bacterial taxa present in the human microbiome (Diakite et al., 2020), still, its application has not been widely explored in plant research. The amalgamation of the present rice root microbiome results with culturomics protocol will open up the avenue for *in vivo* studies leading to the devising of a novel strategy for the development of SynCom-based microbiome technologies for sustainable rice production.

## Data availability statement

The datasets presented in this study can be found in online repositories. The names of the repository/repositories and accession number(s) can be found in the article/supplementary material.

## Ethics statement

This article does not contain any studies with human participants or animals performed by any of the authors.

## Author contributions

ASa and ASr conceptualized and designed the experiments. SK, MZ, and BS conducted the lab work under the supervision of MK and HC. AS, MK, and HC analyzed the data and wrote the manuscript. KP, KR, and AM improved the manuscript draft. All authors contributed to the article and approved the submitted version.

## Funding

Indian Soil Microbiome Project funded by ICAR-NBAIM, Mau, India.

## Conflict of interest

The authors declare that the research was conducted in the absence of any commercial or financial relationships that could be construed as a potential conflict of interest.

The reviewer JC declared a shared affiliation with the authors AS, MK, HC, KP, SK, MZ, BS, KR, ASr, and ASa at the time of review.

## Publisher’s note

All claims expressed in this article are solely those of the authors and do not necessarily represent those of their affiliated organizations, or those of the publisher, the editors and the reviewers. Any product that may be evaluated in this article, or claim that may be made by its manufacturer, is not guaranteed or endorsed by the publisher.

## Footnotes

^1^ https://www.fao.org/statistics/en/

## Supplementary material

SUPPLEMENTARY FIGURE 1 Random forest machine learning coupled rhizo-microbiome co-occurrence network analysis of ten rice varieties, the size of the nodes is arranged based on their betweenness centrality values, nodes having the highest radius are depicted as keystone taxa; HUR 917 **(A)**, MTU1001 **(B)**, BPT5204 **(C)**, Warangal 3207 **(D)**, TKM13 **(E)**, HUR 105 **(F)**, CO52 **(G)**, Rajender Sweta **(H)**, MTU 7029 **(I)** and SHIATS 1 **(H)**.

SUPPLEMENTARY FIGURE 2 Correlogram depicting significant microbiome associations (p < 0.05) of bacterial taxa representing rhizo-microbiome of rice variety HUR 917, p <0.05.

SUPPLEMENTARY FIGURE 3 Correlogram depicting significant microbiome associations of bacterial taxa representing rhizo-microbiome of rice variety MTU1001.

SUPPLEMENTARY FIGURE 4 Correlogram depicting significant microbiome associations of bacterial taxa representing rhizo-microbiome of rice variety BPT5204.

SUPPLEMENTARY FIGURE 5 Correlogram depicting significant microbiome associations of bacterial taxa representing rhizo-microbiome of rice variety Warangal 3207.

SUPPLEMENTARY FIGURE 6 Correlogram depicting significant microbiome associations of bacterial taxa representing rhizo-microbiome of rice variety TKM13.

SUPPLEMENTARY FIGURE 7 Correlogram depicting significant microbiome associations of bacterial taxa representing rhizo-microbiome of rice variety HUR105.

SUPPLEMENTARY FIGURE 8 Correlogram depicting significant microbiome associations of bacterial taxa representing rhizo-microbiome of rice variety CO52.

SUPPLEMENTARY FIGURE 9 Correlogram depicting significant microbiome associations of bacterial taxa representing rhizo-microbiome of rice variety Rajender Sweta.

SUPPLEMENTARY FIGURE 10 Correlogram depicting significant microbiome associations of bacterial taxa representing rhizo-microbiome of rice variety MTU 7029.

SUPPLEMENTARY FIGURE 11 Correlogram depicting significant microbiome associations of bacterial taxa representing rhizo-microbiome of rice variety SHIATS 1.

SUPPLEMENTARY FIGURE 12 Differential abundance of top fifty gene families associated with rhizo-microbiome of rice variety. As revealed by Lefse analysis **(A)** and Heatmap showing the distribution of the top fifty gene families associated with the rhizo-microbiome of rice, the color gradient towards orange and green represents high and low abundance as indicated in the scale bar.

SUPPLEMENTARY DATA FILE 1 XLSX table containing a tab with the full list of the taxa with taxonomy, prevalence, and relative abundance.

SUPPLEMENTARY DATA FILE 2 XLSX table containing a tab with the full list of the genus and mean relative abundance.
