# Supplementary figures and images for: Influence of host genotype in establishing root associated microbiome of indica rice cultivars for plant growth promotion
Source: Front Microbiol. 2022 Nov 14;13:1033158. doi: 10.3389/fmicb.2022.1033158 (PMC9702084; doi:10.3389/fmicb.2022.1033158)

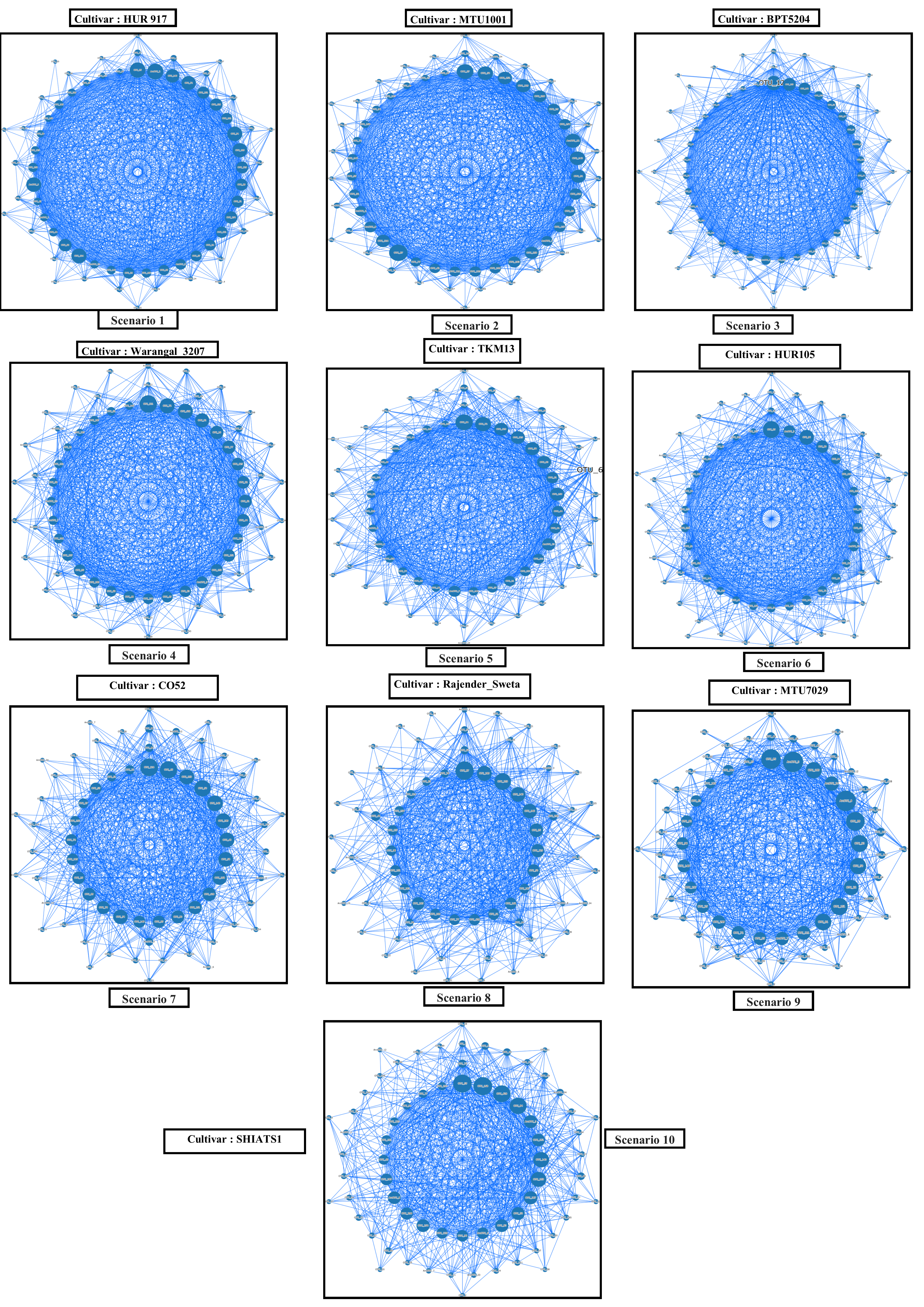

Supplement: SUPPLEMENTARY FIGURE 1 — Random forest machine learning coupled rhizo-microbiome co-occurrence network analysis of ten rice varieties, the size of the nodes is arranged based on their betweenness centrality values, nodes having the highest radius are depicted as keystone taxa; HUR 917 (A), MTU1001 (B), BPT5204 (C), Warangal 3207 (D), TKM13 (E), HUR 105 (F), CO52 (G), Rajender Sweta (H), MTU 7029 (I) and SHIATS 1 (H). [file Image_1.TIF]

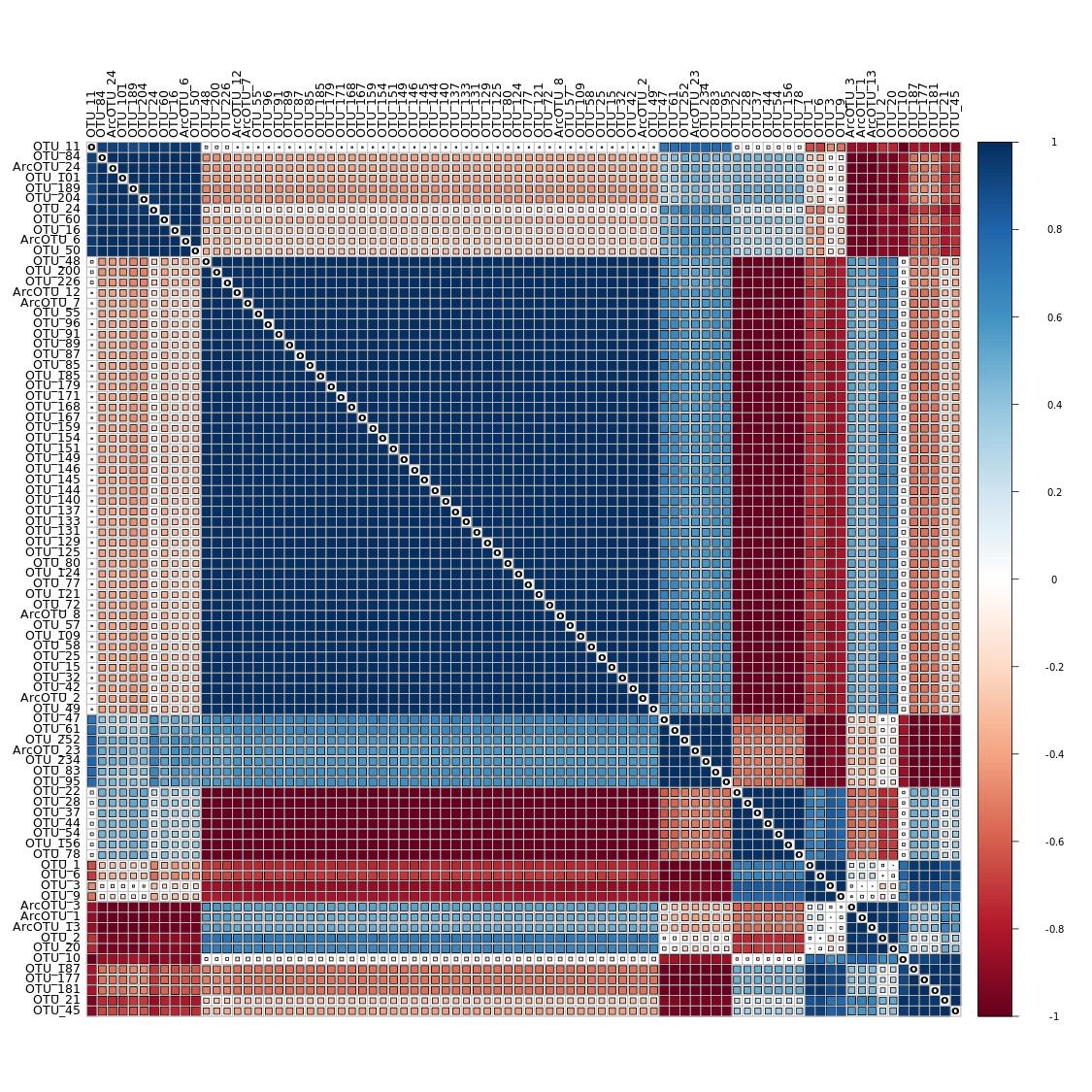

Supplement: SUPPLEMENTARY FIGURE 2 — Correlogram depicting significant microbiome associations (p < 0.05) of bacterial taxa representing rhizo-microbiome of rice variety HUR 917, p <0.05. [file Image_2.JPEG]

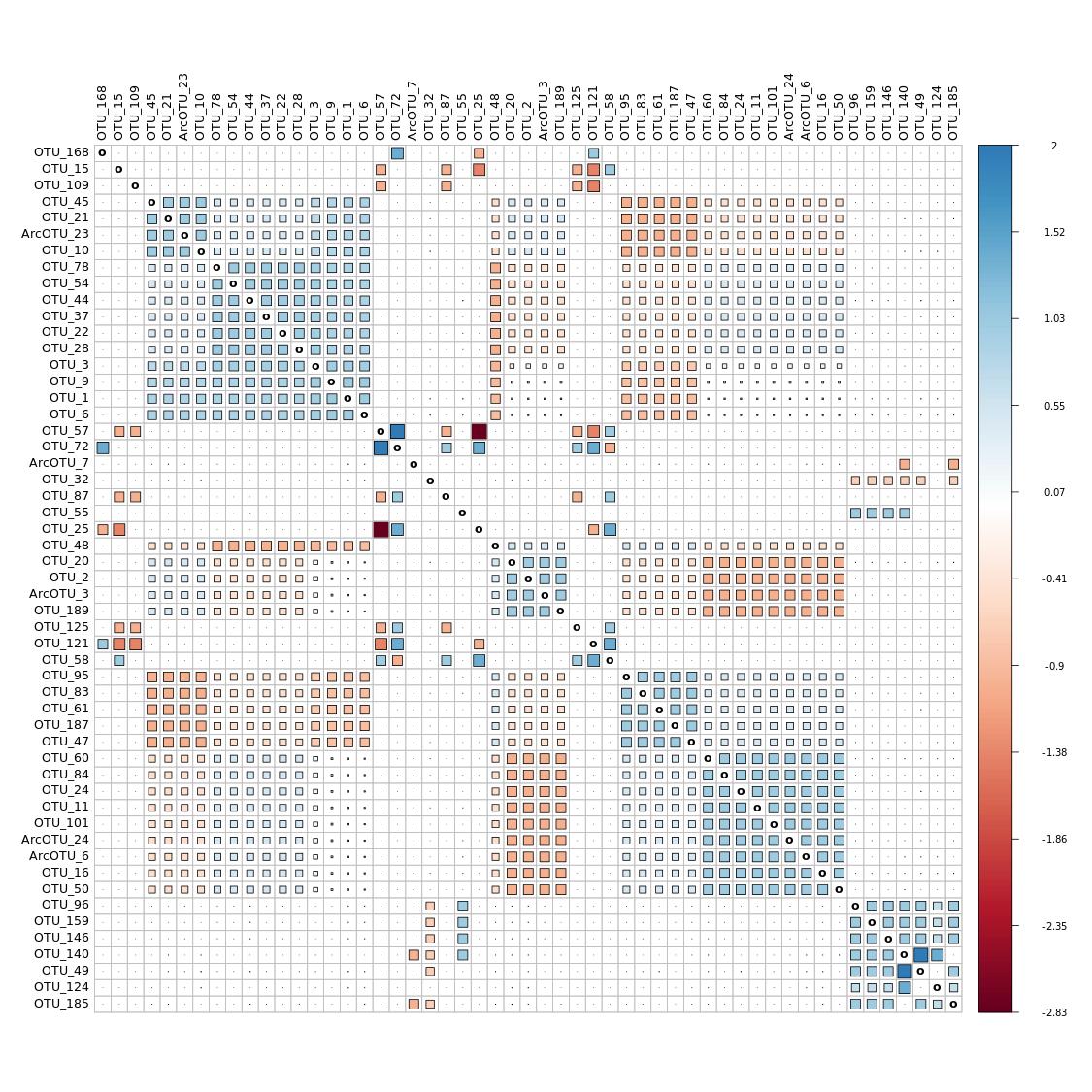

Supplement: SUPPLEMENTARY FIGURE 3 — Correlogram depicting significant microbiome associations of bacterial taxa representing rhizo-microbiome of rice variety MTU1001. [file Image_3.JPEG]

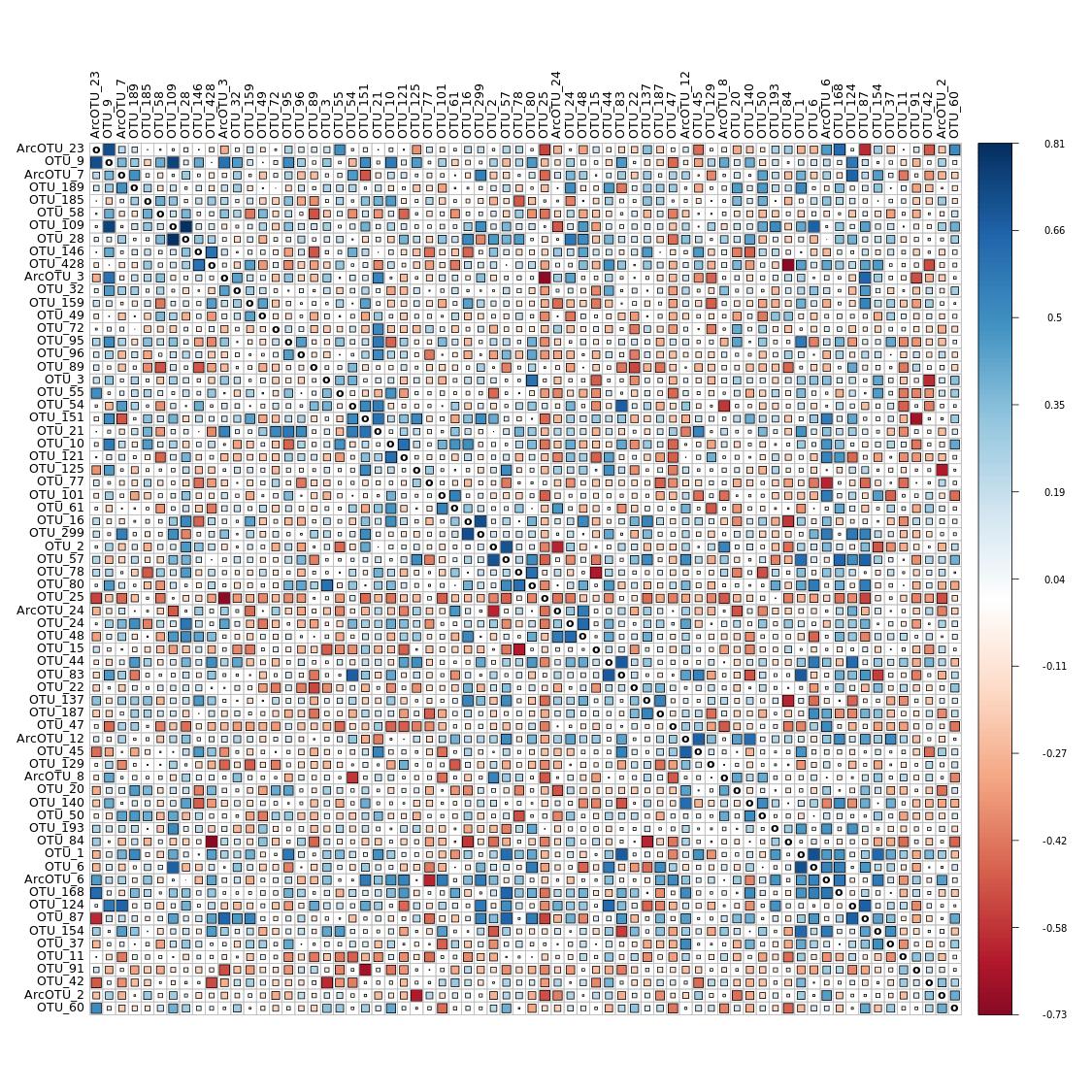

Supplement: SUPPLEMENTARY FIGURE 11 — Correlogram depicting significant microbiome associations of bacterial taxa representing rhizo-microbiome of rice variety SHIATS 1. [file Image_11.JPEG]

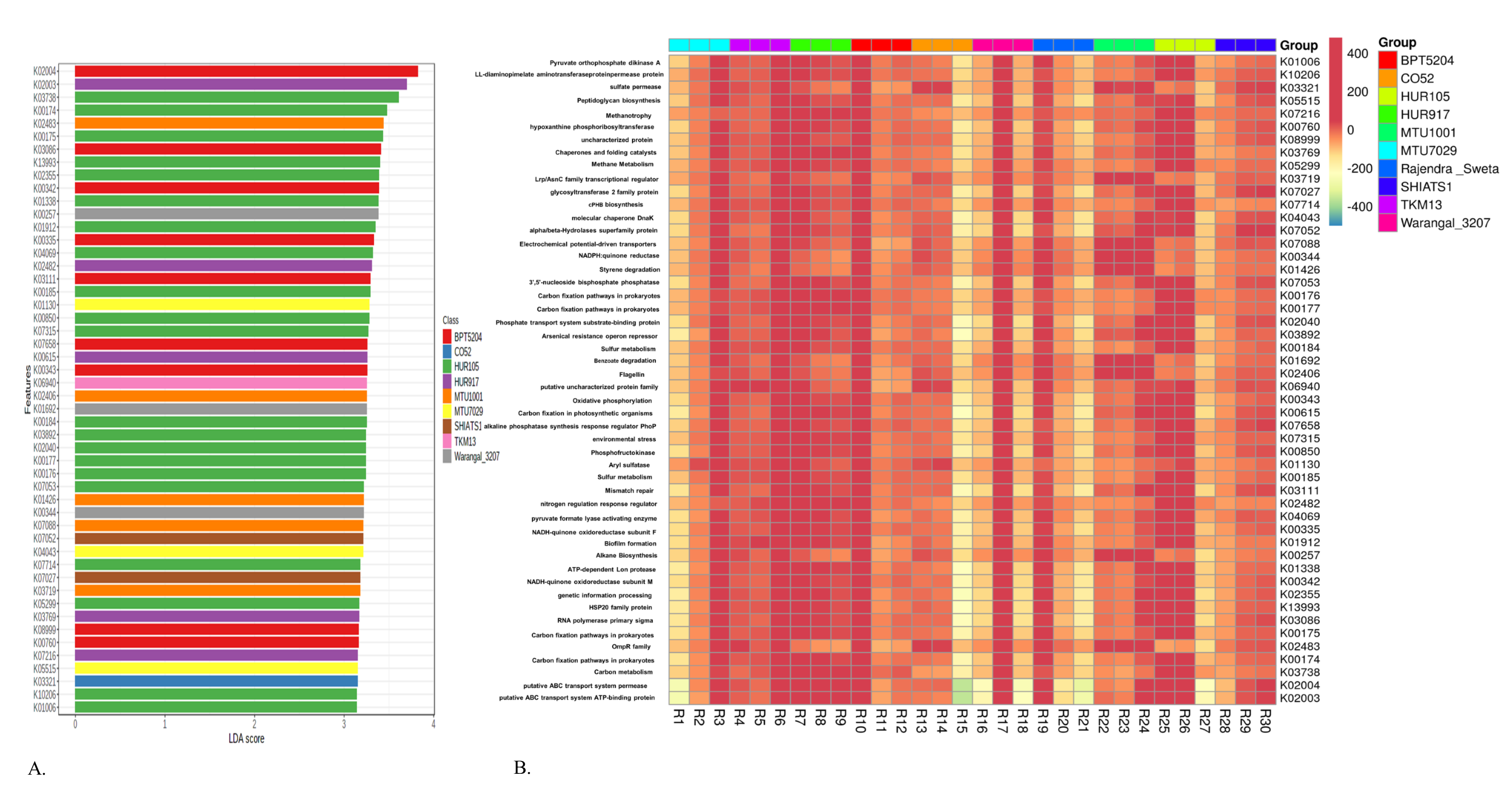

Supplement: SUPPLEMENTARY FIGURE 12 — Differential abundance of top fifty gene families associated with rhizo-microbiome of rice variety. As revealed by Lefse analysis (A) and Heatmap showing the distribution of the top fifty gene families associated with the rhizo-microbiome of rice, the color gradient towards orange and green represents high and low abundance as indicated in the scale bar. [file Image_12.TIF]
