# Supplementary material for: Influence of host genotype in establishing root associated microbiome of indica rice cultivars for plant growth promotion
Source: Front Microbiol. 2022 Nov 14;13:1033158. doi: 10.3389/fmicb.2022.1033158 (PMC9702084; doi:10.3389/fmicb.2022.1033158)
Supplement: Supplementary file 15 [file Data_Sheet_3.DOCX]

Table S1. Distribution of bacterial features in the rhizosphere of the rice cultivars

| **Cultivars** | **Average Bacterial Features** |
| --- | --- |
| MTU7029 | 57335.33 |
| TKM13 | 67487.33 |
| HUR917 | 62359.66 |
| BPT5204 | 64048.66 |
| CO52 | 55823.66 |
| Warangal_3207 | 58664 |
| Rajendra _Sweta | 53101.33 |
| MTU1001 | 63248.66 |
| HUR105 | 57360 |
| SHIATS1 | 62143.66 |

Table S2. Estimation of alpha diversity index of rhizo-microbiome associated with rice varieties, based on Faith phylogenetic distances. Values indicates, mean ± standard deviation

| **Varieties** | **Faith phylogenetic diversity Index** |
| --- | --- |
| BPT5204 | 73.848±2.34 |
| CO52 | 72.344±2.49 |
| HUR105 | 73.197±1.35 |
| HUR917 | 73.789±0.85 |
| MTU1001 | 71.859±1.73 |
| MTU7029 | 74.945±2.08 |
| Rajendra _Sweta | 69.854±0.343 |
| SHIATS1 | 73.928±1.14 |
| TKM13 | 70.722±0.5 |
| Warangal_3207 | 72.076±1.18 |

Table S3. Estimation of network property of co-occurrence microbial network of rice cultivar HUR917

| Node_Index | Label | Taxa | Degree | Cluster_Coeff | Closeness | Betweenness | Eigen_Vector | Eccentricity | Coreness |
| --- | --- | --- | --- | --- | --- | --- | --- | --- | --- |
| 1 | OTU_10 | *Saprospiraceae* | 14 | 0.571429 | 0.553719 | 0.137933 | 0.041546 | 3 | 12 |
| 2 | ArcOTU_12 | *Methanosaeta* | 9 | 0.694444 | 0.523438 | 0.017921 | 0.030897 | 3 | 9 |
| 3 | OTU_101 | *Chloroflexi* | 48 | 0.707447 | 0.77907 | 0.592023 | 0.16197 | 2 | 36 |
| 4 | OTU_121 | *Sinobacteraceae* | 46 | 0.723671 | 0.761364 | 0.613579 | 0.156999 | 2 | 36 |
| 5 | OTU_159 | *Spirochaetales* | 43 | 0.782946 | 0.736264 | 0.437319 | 0.152533 | 2 | 36 |
| 6 | OTU_28 | *Hydrogenophaga* | 45 | 0.732323 | 0.752809 | 0.587413 | 0.154621 | 2 | 36 |
| 7 | OTU_54 | Ellin6529 | 47 | 0.694727 | 0.770115 | 0.899686 | 0.157554 | 2 | 36 |
| 8 | OTU_61 | *Bacteroidales* | 47 | 0.710453 | 0.770115 | 0.652529 | 0.159264 | 2 | 36 |
| 9 | OTU_77 | *Bacillales* | 24 | 0.608696 | 0.609091 | 0.211825 | 0.074451 | 2 | 18 |
| 10 | OTU_89 | S035 | 47 | 0.705828 | 0.770115 | 0.663026 | 0.158623 | 2 | 36 |
| 11 | ArcOTU_23 | *Parvarchaea* | 45 | 0.764646 | 0.752809 | 0.351718 | 0.15787 | 2 | 36 |
| 12 | ArcOTU_2 | *Crenarchaeota* | 50 | 0.647347 | 0.797619 | 1 | 0.161582 | 2 | 36 |
| 13 | ArcOTU_3 | *Candidatus Nitrososphaera* | 46 | 0.730435 | 0.761364 | 0.550637 | 0.158057 | 2 | 36 |
| 14 | ArcOTU_6 | *Methanobacterium* | 43 | 0.777409 | 0.736264 | 0.897302 | 0.152042 | 2 | 36 |
| 15 | ArcOTU_7 | *Methanocella* | 44 | 0.787526 | 0.744444 | 0.308392 | 0.156561 | 2 | 36 |
| 16 | OTU_109 | *Desulfobulbaceae* | 47 | 0.689177 | 0.770115 | 0.87341 | 0.156966 | 2 | 36 |
| 17 | OTU_124 | *Gaiellaceae* | 44 | 0.760042 | 0.744444 | 0.916188 | 0.154102 | 2 | 36 |
| 18 | OTU_125 | *Myxococcales* | 43 | 0.792913 | 0.736264 | 0.352593 | 0.153432 | 2 | 36 |
| 19 | OTU_129 | *Desulfobacteraceae* | 47 | 0.721554 | 0.770115 | 0.665488 | 0.160328 | 2 | 36 |
| 20 | OTU_137 | *Lachnospiraceae* | 41 | 0.859756 | 0.72043 | 0.147259 | 0.152065 | 2 | 36 |
| 21 | OTU_140 | *Koribacteraceae* | 45 | 0.763636 | 0.752809 | 0.381652 | 0.157918 | 2 | 36 |
| 22 | OTU_146 | *Rhodobacteraceae* | 50 | 0.669388 | 0.797619 | 0.804519 | 0.164472 | 2 | 36 |
| 23 | OTU_151 | 44159 | 49 | 0.684524 | 0.788235 | 0.68401 | 0.163157 | 2 | 36 |
| 24 | OTU_154 | C0119 | 46 | 0.722705 | 0.761364 | 0.70563 | 0.157122 | 2 | 36 |
| 25 | OTU_168 | OPB54 | 43 | 0.811739 | 0.736264 | 0.242276 | 0.155223 | 2 | 36 |
| 26 | OTU_185 | *Candidatus Entotheonella* | 42 | 0.804878 | 0.728261 | 0.397097 | 0.151141 | 2 | 36 |
| 27 | OTU_187 | EB1017 | 46 | 0.742029 | 0.761364 | 0.441412 | 0.159232 | 2 | 36 |
| 28 | OTU_189 | *Acidobacteria-6* | 19 | 0.619883 | 0.577586 | 0.164217 | 0.059908 | 3 | 15 |
| 29 | OTU_193 | WD2101 | 17 | 0.639706 | 0.563025 | 0.132688 | 0.05467 | 3 | 14 |
| 30 | OTU_22 | *Chitinophagaceae* | 14 | 0.736264 | 0.553719 | 0.037651 | 0.048649 | 3 | 13 |
| 31 | OTU_24 | *Rhodocyclaceae* | 46 | 0.733333 | 0.761364 | 0.581131 | 0.158113 | 2 | 36 |
| 32 | OTU_299 | *Pseudoxanthomonas* | 42 | 0.813008 | 0.728261 | 0.32623 | 0.151855 | 2 | 36 |
| 33 | OTU_3 | *Anaerolineae* | 15 | 0.619048 | 0.553719 | 0.08106 | 0.046809 | 3 | 13 |
| 34 | OTU_32 | Gemm-5 | 10 | 1 | 0.540323 | 0 | 0.040042 | 2 | 10 |
| Node_Index | Label | Taxa | Degree | Cluster_Coeff | Closeness | Betweenness | Eigen_Vector | Eccentricity | Coreness |
| 35 | OTU_37 | *Rhizobiales* | 42 | 0.829268 | 0.728261 | 0.206725 | 0.153342 | 2 | 36 |
| 36 | OTU_428 | BPC076 | 48 | 0.694149 | 0.77907 | 0.701614 | 0.160827 | 2 | 36 |
| 37 | OTU_44 | *Aeromonadaceae* | 44 | 0.781184 | 0.744444 | 0.377699 | 0.155931 | 2 | 36 |
| 38 | OTU_45 | *Methylosinus* | 44 | 0.788584 | 0.744444 | 0.289474 | 0.156643 | 2 | 36 |
| 39 | OTU_47 | *Acidimicrobiales* | 10 | 0.755556 | 0.531746 | 0.023701 | 0.032398 | 3 | 10 |
| 40 | OTU_48 | DS-18 | 12 | 0.742424 | 0.540323 | 0.037262 | 0.040077 | 3 | 11 |
| 41 | OTU_49 | *Rhodospirillales* | 11 | 0.763636 | 0.540323 | 0.022249 | 0.038322 | 3 | 11 |
| 42 | OTU_50 | *Bacteroidales* | 16 | 0.791667 | 0.567797 | 0.084023 | 0.057269 | 2 | 14 |
| 43 | OTU_55 | *Syntrophobacteraceae* | 22 | 0.662338 | 0.59292 | 0.174159 | 0.072586 | 3 | 18 |
| 44 | OTU_57 | *Geobacteraceae* | 46 | 0.725604 | 0.761364 | 0.569806 | 0.157357 | 2 | 36 |
| 45 | OTU_60 | *Clostridium* | 44 | 0.736786 | 0.744444 | 0.719191 | 0.151659 | 2 | 36 |
| 46 | OTU_72 | *Cyanobacteria* | 49 | 0.677721 | 0.788235 | 0.852728 | 0.162372 | 2 | 36 |
| 47 | OTU_78 | pGrfC26 | 19 | 0.836257 | 0.582609 | 0.0315 | 0.070071 | 2 | 18 |
| 48 | OTU_80 | AKYG1722 | 40 | 0.869231 | 0.712766 | 0.152563 | 0.149238 | 2 | 36 |
| 49 | OTU_83 | *Actinobacteria* | 46 | 0.731401 | 0.761364 | 0.540049 | 0.157894 | 2 | 36 |
| 50 | OTU_87 | *Aeromonas* | 20 | 0.742105 | 0.587719 | 0.081625 | 0.068325 | 2 | 18 |
| 51 | OTU_9 | SJA-15 | 15 | 0.695238 | 0.563025 | 0.089214 | 0.050765 | 2 | 13 |
| 52 | OTU_95 | *Algoriphagus* | 51 | 0.649412 | 0.807229 | 0.963965 | 0.164958 | 2 | 36 |
| 53 | OTU_96 | LCP-6 | 43 | 0.805094 | 0.736264 | 0.273051 | 0.154664 | 2 | 36 |
| 54 | OTU_1 | *Sulfuricurvum* | 21 | 0.804762 | 0.587719 | 0.078816 | 0.075725 | 3 | 18 |
| 55 | OTU_16 | *Geobacter* | 16 | 0.783333 | 0.563025 | 0.03348 | 0.057407 | 3 | 15 |
| 56 | OTU_6 | *Anaerolinea* | 23 | 0.72332 | 0.603604 | 0.181444 | 0.079008 | 2 | 18 |
| 57 | ArcOTU_24 | *Parvarchaea* | 19 | 0.766082 | 0.577586 | 0.052448 | 0.066908 | 3 | 18 |
| 58 | OTU_25 | JG30-KF-CM45 | 8 | 0.714286 | 0.523438 | 0.012743 | 0.027063 | 3 | 8 |
| 59 | OTU_42 | *Kouleothrixaceae* | 22 | 0.792208 | 0.598214 | 0.146828 | 0.077704 | 2 | 18 |
| 60 | OTU_91 | *Peredibacter* | 19 | 0.760234 | 0.577586 | 0.058492 | 0.066573 | 3 | 17 |
| 61 | ArcOTU_8 | *Methanomicrobiales* | 9 | 0.861111 | 0.531746 | 0.006705 | 0.033452 | 3 | 9 |
| 62 | OTU_20 | Ellin515 | 2 | 1 | 0.446667 | 0 | 0.007769 | 3 | 2 |
| 63 | OTU_2 | OD1 | 12 | 0.530303 | 0.540323 | 0.056225 | 0.035103 | 3 | 12 |
| 64 | OTU_58 | Ellin6075 | 12 | 0.878788 | 0.536 | 0.008032 | 0.043955 | 3 | 12 |
| 65 | OTU_84 | *Luteolibacter* | 20 | 0.784211 | 0.582609 | 0.073832 | 0.070487 | 3 | 18 |
| 66 | OTU_15 | *Anaerolineae* | 8 | 0.892857 | 0.527559 | 0.003005 | 0.029705 | 3 | 8 |
| 67 | OTU_21 | *Clostridiaceae* | 7 | 0.571429 | 0.51145 | 0.013399 | 0.022843 | 3 | 7 |
| 68 | OTU_11 | envOPS12 | 6 | 0.733333 | 0.507576 | 0.004889 | 0.019931 | 3 | 6 |

Table S4. Estimation of network property of co-occurrence microbial network of rice cultivar MTU1001.

| Node_Index | Label | Taxa | Degree | Cluster_Coeff | Closeness | Betweenness | Eigen_Vector | Eccentricity | Coreness |
| --- | --- | --- | --- | --- | --- | --- | --- | --- | --- |
| 1 | ArcOTU_2 | *Crenarchaeota* | 43 | 0.718715 | 0.736264 | 0.492267 | 0.160184 | 2 | 33 |
| 2 | ArcOTU_12 | *Methanosaeta* | 45 | 0.671717 | 0.752809 | 0.79823 | 0.162209 | 2 | 33 |
| 3 | ArcOTU_3 | *Candidatus Nitrososphaera* | 17 | 0.786765 | 0.567797 | 0.031867 | 0.066785 | 3 | 15 |
| 4 | ArcOTU_7 | *Methanocella* | 45 | 0.693939 | 0.752809 | 0.46729 | 0.164762 | 2 | 33 |
| 5 | ArcOTU_8 | *Methanomicrobiales* | 43 | 0.728682 | 0.736264 | 0.533223 | 0.160987 | 2 | 33 |
| 6 | OTU_109 | *Desulfobulbaceae* | 44 | 0.707188 | 0.744444 | 0.489057 | 0.162203 | 2 | 33 |
| 7 | OTU_11 | envOPS12 | 12 | 1 | 0.536 | 0 | 0.052463 | 3 | 12 |
| 8 | OTU_121 | *Sinobacteraceae* | 44 | 0.710359 | 0.744444 | 0.451084 | 0.162717 | 2 | 33 |
| 9 | OTU_124 | *Gaiellaceae* | 43 | 0.704319 | 0.736264 | 0.667531 | 0.158668 | 2 | 33 |
| 10 | OTU_125 | *Myxococcales* | 44 | 0.707188 | 0.744444 | 0.486575 | 0.162524 | 2 | 33 |
| 11 | OTU_129 | *Desulfobacteraceae* | 46 | 0.66087 | 0.761364 | 0.715323 | 0.164376 | 2 | 33 |
| 12 | OTU_137 | *Lachnospiraceae* | 45 | 0.691919 | 0.752809 | 0.499133 | 0.164333 | 2 | 33 |
| 13 | OTU_140 | *Koribacteraceae* | 45 | 0.650505 | 0.752809 | 0.769986 | 0.159809 | 2 | 33 |
| 14 | OTU_146 | *Rhodobacteraceae* | 42 | 0.75029 | 0.728261 | 0.346965 | 0.159625 | 2 | 33 |
| 15 | OTU_15 | *Anaerolineae* | 39 | 0.82996 | 0.705263 | 0.164705 | 0.155511 | 2 | 33 |
| 16 | OTU_151 | 44159 | 47 | 0.649399 | 0.770115 | 0.677955 | 0.166739 | 2 | 33 |
| 17 | OTU_154 | C0119 | 46 | 0.677295 | 0.761364 | 0.519593 | 0.166559 | 2 | 33 |
| 18 | OTU_159 | *Spirochaetales* | 44 | 0.682875 | 0.744444 | 0.661747 | 0.159891 | 2 | 33 |
| 19 | OTU_16 | *Geobacter* | 11 | 0.709091 | 0.536 | 0.020009 | 0.041961 | 3 | 11 |
| 20 | OTU_168 | OPB54 | 41 | 0.760976 | 0.72043 | 0.265798 | 0.15708 | 2 | 33 |
| 21 | OTU_185 | *Candidatus Entotheonella* | 40 | 0.81282 | 0.712766 | 0.184286 | 0.157812 | 2 | 33 |
| 22 | OTU_187 | EB1017 | 14 | 0.879121 | 0.553719 | 0.017721 | 0.058208 | 3 | 13 |
| 23 | OTU_193 | WD2101 | 44 | 0.70296 | 0.744444 | 0.550419 | 0.161859 | 2 | 33 |
| 24 | OTU_20 | Ellin515 | 8 | 0.428571 | 0.531746 | 0.034602 | 0.025013 | 2 | 8 |
| 25 | OTU_24 | *Rhodocyclaceae* | 13 | 0.538462 | 0.553719 | 0.166029 | 0.042963 | 2 | 11 |
| 26 | OTU_25 | JG30-KF-CM45 | 46 | 0.675362 | 0.761364 | 0.555475 | 0.166241 | 2 | 33 |
| 27 | OTU_299 | *Pseudoxanthomonas* | 46 | 0.666667 | 0.761364 | 0.617162 | 0.165046 | 2 | 33 |
| 28 | OTU_32 | Gemm-5 | 43 | 0.728682 | 0.736264 | 0.407578 | 0.160862 | 2 | 33 |
| 29 | OTU_37 | *Rhizobiales* | 16 | 0.841667 | 0.567797 | 0.020586 | 0.064447 | 2 | 15 |
| 30 | OTU_42 | *Kouleothrixaceae* | 44 | 0.714588 | 0.744444 | 0.430923 | 0.162881 | 2 | 33 |
| 31 | OTU_428 | BPC076 | 45 | 0.679798 | 0.752809 | 0.571372 | 0.163044 | 2 | 33 |
| 32 | OTU_44 | *Aeromonadaceae* | 16 | 0.658333 | 0.567797 | 0.082398 | 0.057282 | 2 | 13 |
| 33 | OTU_49 | *Rhodospirillales* | 39 | 0.804318 | 0.705263 | 0.186167 | 0.153326 | 2 | 33 |
| Node_Index | Label | Taxa | Degree | Cluster_Coeff | Closeness | Betweenness | Eigen_Vector | Eccentricity | Coreness |
| 34 | OTU_54 | Ellin6529 | 16 | 0.7 | 0.567797 | 0.060463 | 0.058627 | 2 | 14 |
| 35 | OTU_55 | *Syntrophobacteraceae* | 42 | 0.749129 | 0.728261 | 0.368825 | 0.159341 | 2 | 33 |
| 36 | OTU_57 | *Geobacteraceae* | 48 | 0.631206 | 0.77907 | 0.869444 | 0.16754 | 2 | 33 |
| 37 | OTU_58 | Ellin6075 | 47 | 0.641998 | 0.770115 | 0.769762 | 0.165717 | 2 | 33 |
| 38 | OTU_6 | *Anaerolinea* | 4 | 0.5 | 0.468531 | 0.002531 | 0.014903 | 3 | 4 |
| 39 | OTU_61 | *Bacteroidales* | 16 | 0.75 | 0.567797 | 0.049243 | 0.060687 | 2 | 14 |
| 40 | OTU_72 | *Cyanobacteria* | 41 | 0.77561 | 0.72043 | 0.273513 | 0.157988 | 2 | 33 |
| 41 | OTU_77 | *Bacillales* | 44 | 0.718816 | 0.744444 | 0.400419 | 0.163364 | 2 | 33 |
| 42 | OTU_80 | AKYG1722 | 38 | 0.857753 | 0.697917 | 0.129124 | 0.153953 | 2 | 33 |
| 43 | OTU_87 | *Aeromonas* | 43 | 0.683278 | 0.736264 | 1 | 0.15634 | 2 | 33 |
| 44 | OTU_89 | S035 | 45 | 0.69495 | 0.752809 | 0.506376 | 0.164105 | 2 | 33 |
| 45 | OTU_91 | *Peredibacter* | 45 | 0.651515 | 0.752809 | 0.718558 | 0.159967 | 2 | 33 |
| 46 | OTU_96 | LCP-6 | 41 | 0.784146 | 0.72043 | 0.240672 | 0.159004 | 2 | 33 |
| 47 | ArcOTU_24 | *Parvarchaea* | 21 | 0.671429 | 0.59292 | 0.129211 | 0.076006 | 2 | 16 |
| 48 | OTU_28 | *Hydrogenophaga* | 13 | 0.628205 | 0.544715 | 0.055324 | 0.047331 | 3 | 11 |
| 49 | OTU_47 | *Acidimicrobiales* | 5 | 1 | 0.5 | 0 | 0.021813 | 3 | 5 |
| 50 | OTU_78 | pGrfC26 | 10 | 0.822222 | 0.527559 | 0.017666 | 0.04016 | 3 | 9 |
| 51 | OTU_95 | *Algoriphagus* | 11 | 0.672727 | 0.531746 | 0.022203 | 0.039864 | 3 | 11 |
| 52 | ArcOTU_23 | *Parvarchaea* | 16 | 0.683333 | 0.563025 | 0.046044 | 0.058754 | 3 | 14 |
| 53 | OTU_189 | *Acidobacteria-6* | 14 | 0.725275 | 0.553719 | 0.028504 | 0.052896 | 3 | 14 |
| 54 | OTU_21 | *Clostridiaceae* | 14 | 0.89011 | 0.54918 | 0.010194 | 0.058117 | 3 | 14 |
| 55 | OTU_50 | *Bacteroidales* | 19 | 0.730994 | 0.582609 | 0.095458 | 0.07068 | 2 | 15 |
| 56 | OTU_1 | *Sulfuricurvum* | 12 | 0.575758 | 0.540323 | 0.143179 | 0.037855 | 3 | 11 |
| 57 | OTU_2 | OD1 | 11 | 0.727273 | 0.536 | 0.01662 | 0.042553 | 3 | 11 |
| 58 | OTU_22 | *Chitinophagaceae* | 9 | 0.555556 | 0.523438 | 0.01686 | 0.031649 | 3 | 9 |
| 59 | OTU_45 | *Methylosinus* | 17 | 0.757353 | 0.567797 | 0.043762 | 0.065388 | 3 | 15 |
| 60 | OTU_101 | *Chloroflexi* | 21 | 0.566667 | 0.587719 | 0.219086 | 0.070702 | 3 | 15 |
| 61 | ArcOTU_6 | *Methanobacterium* | 12 | 0.712121 | 0.544715 | 0.12081 | 0.045694 | 3 | 11 |
| 62 | OTU_3 | *Anaerolineae* | 4 | 0.166667 | 0.455782 | 0.019034 | 0.007612 | 3 | 4 |
| 63 | OTU_48 | DS-18 | 15 | 0.828571 | 0.563025 | 0.025381 | 0.059721 | 2 | 14 |
| 64 | OTU_60 | *Clostridium* | 12 | 0.984848 | 0.54918 | 0.000373 | 0.052126 | 2 | 12 |
| 65 | OTU_83 | *Actinobacteria* | 17 | 0.727941 | 0.567797 | 0.06366 | 0.062618 | 3 | 15 |
| 66 | OTU_84 | *Luteolibacter* | 17 | 0.764706 | 0.567797 | 0.042891 | 0.065446 | 3 | 15 |
| 67 | OTU_10 | *Saprospiraceae* | 10 | 0.711111 | 0.536 | 0.013706 | 0.038229 | 3 | 10 |
| 68 | OTU_9 | SJA-15 | 8 | 0.821429 | 0.51938 | 0.005446 | 0.032289 | 3 | 8 |

Table S5. Estimation of network property of co-occurrence microbial network of rice cultivar BPT5204.

| Node Index | Label | Taxa | Degree | Cluster_Coeff | Closeness | Betweenness | Eigen_Vector | Eccentricity | Coreness |
| --- | --- | --- | --- | --- | --- | --- | --- | --- | --- |
| 1 | ArcOTU_2 | *Crenarchaeota* | 11 | 0.818182 | 0.540323 | 0.008986 | 0.042268 | 3 | 11 |
| 2 | ArcOTU_12 | *Methanosaeta* | 45 | 0.685859 | 0.752809 | 0.431305 | 0.163842 | 2 | 34 |
| 3 | ArcOTU_23 | *Parvarchaea* | 41 | 0.806098 | 0.72043 | 0.140693 | 0.16122 | 2 | 34 |
| 4 | ArcOTU_3 | *Candidatus Nitrososphaera* | 12 | 0.363636 | 0.531746 | 0.114671 | 0.032901 | 3 | 10 |
| 5 | ArcOTU_6 | *Methanobacterium* | 42 | 0.751452 | 0.728261 | 0.335029 | 0.159852 | 2 | 34 |
| 6 | ArcOTU_7 | *Methanocella* | 42 | 0.716609 | 0.728261 | 0.533623 | 0.156058 | 2 | 34 |
| 7 | ArcOTU_8 | *Methanomicrobiales* | 40 | 0.821795 | 0.712766 | 0.151854 | 0.15881 | 2 | 34 |
| 8 | OTU_10 | *Saprospiraceae* | 11 | 0.654545 | 0.531746 | 0.030986 | 0.039206 | 3 | 10 |
| 9 | OTU_101 | *Chloroflexi* | 41 | 0.790244 | 0.72043 | 0.25726 | 0.159562 | 2 | 34 |
| 10 | OTU_109 | *Desulfobulbaceae* | 44 | 0.730444 | 0.744444 | 0.269567 | 0.164901 | 2 | 34 |
| 11 | OTU_121 | *Sinobacteraceae* | 10 | 0.577778 | 0.527559 | 0.028761 | 0.033945 | 3 | 9 |
| 12 | OTU_124 | *Gaiellaceae* | 49 | 0.619048 | 0.788235 | 1 | 0.169844 | 2 | 34 |
| 13 | OTU_125 | *Myxococcales* | 47 | 0.663275 | 0.770115 | 0.458848 | 0.168306 | 2 | 34 |
| 14 | OTU_129 | *Desulfobacteraceae* | 41 | 0.791463 | 0.72043 | 0.202963 | 0.160008 | 2 | 34 |
| 15 | OTU_137 | *Lachnospiraceae* | 15 | 0.8 | 0.553719 | 0.019185 | 0.05951 | 3 | 14 |
| 16 | OTU_140 | *Koribacteraceae* | 40 | 0.823077 | 0.712766 | 0.180466 | 0.158809 | 2 | 34 |
| 17 | OTU_151 | Unclassified taxa 44159 | 43 | 0.745293 | 0.736264 | 0.26781 | 0.163042 | 2 | 34 |
| 18 | OTU_154 | C0119 | 44 | 0.716702 | 0.744444 | 0.403349 | 0.163627 | 2 | 34 |
| 19 | OTU_159 | *Spirochaetales* | 49 | 0.587585 | 0.788235 | 0.856684 | 0.165672 | 2 | 34 |
| 20 | OTU_168 | OPB54 | 47 | 0.650324 | 0.770115 | 0.771543 | 0.166878 | 2 | 34 |
| 21 | OTU_185 | *Candidatus Entotheonella* | 14 | 0.714286 | 0.54918 | 0.043049 | 0.05201 | 3 | 12 |
| 22 | OTU_193 | WD2101 | 38 | 0.886202 | 0.697917 | 0.084737 | 0.156341 | 2 | 34 |
| 23 | OTU_20 | Ellin515 | 40 | 0.785897 | 0.712766 | 0.233396 | 0.155507 | 2 | 34 |
| 24 | OTU_21 | *Clostridiaceae* | 38 | 0.876245 | 0.697917 | 0.138266 | 0.155616 | 2 | 34 |
| 25 | OTU_22 | *Chitinophagaceae* | 11 | 0.509091 | 0.531746 | 0.059207 | 0.034901 | 3 | 10 |
| 26 | OTU_24 | *Rhodocyclaceae* | 12 | 0.772727 | 0.536 | 0.018655 | 0.046204 | 3 | 10 |
| 27 | OTU_299 | *Pseudoxanthomonas* | 45 | 0.707071 | 0.752809 | 0.324039 | 0.16641 | 2 | 34 |
| 28 | OTU_3 | *Anaerolineae* | 16 | 0.575 | 0.54918 | 0.059405 | 0.055112 | 3 | 14 |
| 29 | OTU_32 | Gemm-5 | 46 | 0.671498 | 0.761364 | 0.485914 | 0.165488 | 2 | 34 |
| 30 | OTU_428 | BPC076 | 41 | 0.80122 | 0.72043 | 0.150012 | 0.160825 | 2 | 34 |
| 31 | OTU_44 | *Aeromonadaceae* | 44 | 0.688161 | 0.744444 | 0.469957 | 0.160497 | 2 | 34 |
| 32 | OTU_45 | *Methylosinus* | 39 | 0.850202 | 0.705263 | 0.141623 | 0.157167 | 2 | 34 |
| 33 | OTU_50 | *Bacteroidales* | 43 | 0.735327 | 0.736264 | 0.346071 | 0.161844 | 2 | 34 |
| 34 | OTU_54 | Ellin6529 | 40 | 0.838462 | 0.712766 | 0.100214 | 0.16039 | 2 | 34 |
| Node Index | Label | Taxa | Degree | Cluster_Coeff | Closeness | Betweenness | Eigen_Vector | Eccentricity | Coreness |
| 35 | OTU_57 | *Geobacteraceae* | 43 | 0.737542 | 0.736264 | 0.307928 | 0.162176 | 2 | 34 |
| 36 | OTU_60 | *Clostridium* | 39 | 0.851552 | 0.705263 | 0.160186 | 0.157558 | 2 | 34 |
| 37 | OTU_61 | *Bacteroidales* | 45 | 0.682828 | 0.752809 | 0.514478 | 0.163367 | 2 | 34 |
| 38 | OTU_72 | *Cyanobacteria* | 43 | 0.734219 | 0.736264 | 0.354956 | 0.161817 | 2 | 34 |
| 39 | OTU_77 | *Bacillales* | 43 | 0.746401 | 0.736264 | 0.317568 | 0.163026 | 2 | 34 |
| 40 | OTU_80 | AKYG1722 | 42 | 0.778165 | 0.728261 | 0.179062 | 0.162323 | 2 | 34 |
| 41 | OTU_83 | *Actinobacteria* | 40 | 0.816667 | 0.712766 | 0.255085 | 0.158372 | 2 | 34 |
| 42 | OTU_84 | *Luteolibacter* | 43 | 0.743079 | 0.736264 | 0.285829 | 0.162677 | 2 | 34 |
| 43 | OTU_87 | *Aeromonas* | 46 | 0.672464 | 0.761364 | 0.474495 | 0.165762 | 2 | 34 |
| 44 | OTU_89 | S035 | 36 | 0.961905 | 0.683673 | 0.012982 | 0.154214 | 2 | 34 |
| 45 | OTU_91 | *Peredibacter* | 11 | 0.781818 | 0.527559 | 0.020413 | 0.04163 | 3 | 10 |
| 46 | OTU_96 | LCP-6 | 40 | 0.820513 | 0.712766 | 0.158798 | 0.158606 | 2 | 34 |
| 47 | OTU_37 | *Rhizobiales* | 14 | 0.604396 | 0.553719 | 0.083646 | 0.047269 | 3 | 11 |
| 48 | OTU_42 | *Kouleothrixaceae* | 15 | 0.72381 | 0.558333 | 0.029505 | 0.056764 | 3 | 14 |
| 49 | OTU_58 | Ellin6075 | 18 | 0.830065 | 0.57265 | 0.023502 | 0.073158 | 3 | 16 |
| 50 | OTU_9 | SJA-15 | 15 | 0.780952 | 0.558333 | 0.026658 | 0.05938 | 3 | 14 |
| 51 | OTU_1 | *Sulfuricurvum* | 20 | 0.626316 | 0.582609 | 0.126624 | 0.070546 | 3 | 16 |
| 52 | ArcOTU_24 | *Parvarchaea* | 20 | 0.621053 | 0.587719 | 0.080772 | 0.070543 | 2 | 16 |
| 53 | OTU_187 | EB1017 | 14 | 0.626374 | 0.54918 | 0.076782 | 0.048316 | 3 | 11 |
| 54 | OTU_11 | envOPS12 | 2 | 0 | 0.452703 | 0.000824 | 0.00546 | 3 | 2 |
| 55 | OTU_48 | DS-18 | 8 | 0.607143 | 0.503759 | 0.007867 | 0.029522 | 3 | 8 |
| 56 | OTU_95 | *Algoriphagus* | 5 | 1 | 0.489051 | 0 | 0.022026 | 3 | 5 |
| 57 | OTU_15 | *Anaerolineae* | 5 | 1 | 0.492647 | 0 | 0.02156 | 3 | 5 |
| 58 | OTU_25 | JG30-KF-CM45 | 4 | 0.5 | 0.462069 | 0.0026 | 0.01428 | 3 | 4 |
| 59 | OTU_49 | *Rhodospirillales* | 6 | 0.4 | 0.496296 | 0.010082 | 0.017284 | 3 | 6 |
| 60 | OTU_47 | *Acidimicrobiales* | 9 | 1 | 0.523438 | 0 | 0.039533 | 3 | 9 |
| 61 | OTU_146 | *Rhodobacteraceae* | 13 | 0.705128 | 0.544715 | 0.032253 | 0.047176 | 3 | 11 |
| 62 | OTU_189 | *Acidobacteria-6* | 12 | 0.636364 | 0.531746 | 0.022864 | 0.043792 | 3 | 11 |
| 63 | OTU_28 | *Hydrogenophaga* | 9 | 0.5 | 0.507576 | 0.023286 | 0.029664 | 3 | 9 |
| 64 | OTU_55 | *Syntrophobacteraceae* | 5 | 0.7 | 0.475177 | 0.002334 | 0.018738 | 3 | 5 |
| 65 | OTU_78 | pGrfC26 | 12 | 0.833333 | 0.536 | 0.039284 | 0.048963 | 3 | 11 |
| 66 | OTU_6 | *Anaerolinea* | 12 | 0.893939 | 0.54918 | 0.005872 | 0.050289 | 2 | 12 |
| 67 | OTU_16 | *Geobacter* | 7 | 0.666667 | 0.503759 | 0.010104 | 0.023806 | 3 | 7 |
| 68 | OTU_2 | OD1 | 5 | 0.7 | 0.478571 | 0.002648 | 0.018753 | 3 | 5 |

Table S6. Estimation of network property of co-occurrence microbial network of rice cultivar Warangal_3207

| Node_Index | Label | Taxa | Degree | Cluster_Coeff | Closeness | Betweenness | Eigen_Vector | Eccentricity | Coreness |
| --- | --- | --- | --- | --- | --- | --- | --- | --- | --- |
| 1 | ArcOTU_23 | *Parvarchaea* | 40 | 0.737179 | 0.712766 | 0.345942 | 0.163661 | 2 | 30 |
| 2 | ArcOTU_12 | *Methanosaeta* | 19 | 0.783626 | 0.582609 | 0.063894 | 0.079957 | 2 | 16 |
| 3 | OTU_10 | *Saprospiraceae* | 12 | 0.378788 | 0.540323 | 0.090663 | 0.037631 | 3 | 10 |
| 4 | OTU_109 | *Desulfobulbaceae* | 41 | 0.704878 | 0.72043 | 0.400797 | 0.164446 | 2 | 30 |
| 5 | OTU_121 | *Sinobacteraceae* | 48 | 0.575355 | 0.77907 | 1 | 0.174686 | 2 | 30 |
| 6 | OTU_125 | *Myxococcales* | 45 | 0.634343 | 0.752809 | 0.624168 | 0.171124 | 2 | 30 |
| 7 | OTU_137 | *Lachnospiraceae* | 41 | 0.7 | 0.72043 | 0.494011 | 0.163808 | 2 | 30 |
| 8 | OTU_16 | *Geobacter* | 45 | 0.626263 | 0.752809 | 0.680392 | 0.170326 | 2 | 30 |
| 9 | OTU_168 | OPB54 | 14 | 0.846154 | 0.558333 | 0.019178 | 0.061003 | 2 | 13 |
| 10 | OTU_185 | *Candidatus Entotheonella* | 39 | 0.765182 | 0.705263 | 0.221662 | 0.162456 | 2 | 30 |
| 11 | OTU_189 | *Acidobacteria-6* | 42 | 0.688734 | 0.728261 | 0.441478 | 0.166272 | 2 | 30 |
| 12 | OTU_21 | *Clostridiaceae* | 39 | 0.747638 | 0.705263 | 0.264954 | 0.16061 | 2 | 30 |
| 13 | OTU_42 | *Kouleothrixaceae* | 10 | 0.555556 | 0.540323 | 0.045148 | 0.03788 | 2 | 9 |
| 14 | OTU_428 | BPC076 | 46 | 0.615459 | 0.761364 | 0.817523 | 0.172458 | 2 | 30 |
| 15 | OTU_44 | *Aeromonadaceae* | 44 | 0.637421 | 0.744444 | 0.629198 | 0.168071 | 2 | 30 |
| 16 | OTU_48 | DS-18 | 42 | 0.665505 | 0.728261 | 0.547046 | 0.163864 | 2 | 30 |
| 17 | OTU_58 | Ellin6075 | 40 | 0.732051 | 0.712766 | 0.329036 | 0.16326 | 2 | 30 |
| 18 | OTU_72 | *Cyanobacteria* | 47 | 0.588344 | 0.770115 | 0.813579 | 0.173112 | 2 | 30 |
| 19 | OTU_77 | *Bacillales* | 45 | 0.617172 | 0.752809 | 0.677884 | 0.169296 | 2 | 30 |
| 20 | OTU_91 | *Peredibacter* | 45 | 0.59697 | 0.752809 | 0.748114 | 0.166178 | 2 | 30 |
| 21 | ArcOTU_2 | *Crenarchaeota* | 14 | 0.43956 | 0.558333 | 0.109717 | 0.043664 | 2 | 12 |
| 22 | ArcOTU_6 | *Methanobacterium* | 10 | 0.688889 | 0.531746 | 0.028407 | 0.039774 | 3 | 9 |
| 23 | OTU_151 | 44159 | 43 | 0.634551 | 0.736264 | 0.609635 | 0.164023 | 2 | 30 |
| 24 | OTU_2 | OD1 | 15 | 0.580952 | 0.558333 | 0.076449 | 0.053172 | 3 | 13 |
| 25 | OTU_22 | *Chitinophagaceae* | 45 | 0.626263 | 0.752809 | 0.624092 | 0.170161 | 2 | 30 |
| 26 | OTU_24 | *Rhodocyclaceae* | 13 | 0.653846 | 0.553719 | 0.042132 | 0.049283 | 2 | 12 |
| 27 | OTU_299 | *Pseudoxanthomonas* | 13 | 0.782051 | 0.544715 | 0.023143 | 0.055187 | 3 | 12 |
| 28 | OTU_32 | Gemm-5 | 19 | 0.526316 | 0.582609 | 0.173537 | 0.064926 | 2 | 14 |
| 29 | OTU_49 | *Rhodospirillales* | 21 | 0.57619 | 0.59292 | 0.15951 | 0.075803 | 2 | 14 |
| 30 | OTU_50 | *Bacteroidales* | 38 | 0.761024 | 0.697917 | 0.322446 | 0.158063 | 2 | 30 |
| 31 | OTU_84 | *Luteolibacter* | 10 | 0.333333 | 0.531746 | 0.108911 | 0.027144 | 3 | 9 |
| 32 | ArcOTU_3 | *Candidatus Nitrososphaera* | 43 | 0.66113 | 0.736264 | 0.566721 | 0.166908 | 2 | 30 |
| 33 | ArcOTU_7 | *Methanocella* | 40 | 0.723077 | 0.712766 | 0.35313 | 0.161839 | 2 | 30 |
| 34 | ArcOTU_8 | *Methanomicrobiales* | 17 | 0.875 | 0.567797 | 0.019114 | 0.075708 | 3 | 16 |
| Node_Index | Label | Taxa | Degree | Cluster_Coeff | Closeness | Betweenness | Eigen_Vector | Eccentricity | Coreness |
| 35 | OTU_101 | *Chloroflexi* | 40 | 0.733333 | 0.712766 | 0.312899 | 0.163068 | 2 | 30 |
| 36 | OTU_124 | *Gaiellaceae* | 44 | 0.664905 | 0.744444 | 0.445504 | 0.171404 | 2 | 30 |
| 37 | OTU_129 | *Desulfobacteraceae* | 19 | 0.824561 | 0.582609 | 0.032892 | 0.082298 | 2 | 17 |
| 38 | OTU_140 | *Koribacteraceae* | 38 | 0.762447 | 0.697917 | 0.262051 | 0.158028 | 2 | 30 |
| 39 | OTU_154 | C0119 | 43 | 0.675526 | 0.736264 | 0.469582 | 0.168531 | 2 | 30 |
| 40 | OTU_159 | *Spirochaetales* | 43 | 0.66113 | 0.736264 | 0.592683 | 0.167024 | 2 | 30 |
| 41 | OTU_187 | EB1017 | 38 | 0.755334 | 0.697917 | 0.34626 | 0.157425 | 2 | 30 |
| 42 | OTU_25 | JG30-KF-CM45 | 8 | 0.75 | 0.51938 | 0.010018 | 0.034429 | 3 | 8 |
| 43 | OTU_45 | *Methylosinus* | 43 | 0.663344 | 0.736264 | 0.515862 | 0.167302 | 2 | 30 |
| 44 | OTU_57 | *Geobacteraceae* | 26 | 0.827692 | 0.62037 | 0.053397 | 0.113057 | 2 | 22 |
| 45 | OTU_6 | *Anaerolinea* | 15 | 0.885714 | 0.563025 | 0.028238 | 0.066563 | 2 | 14 |
| 46 | OTU_60 | *Clostridium* | 42 | 0.674797 | 0.728261 | 0.469514 | 0.165191 | 2 | 30 |
| 47 | OTU_61 | *Bacteroidales* | 44 | 0.636364 | 0.744444 | 0.671684 | 0.167852 | 2 | 30 |
| 48 | OTU_83 | *Actinobacteria* | 43 | 0.668882 | 0.736264 | 0.527969 | 0.167877 | 2 | 30 |
| 49 | OTU_89 | *Sphingomonadales* | 10 | 0.4 | 0.527559 | 0.074097 | 0.033544 | 3 | 9 |
| 50 | OTU_95 | *Algoriphagus* | 41 | 0.687805 | 0.72043 | 0.549671 | 0.1623 | 2 | 30 |
| 51 | ArcOTU_24 | *Parvarchaea* | 15 | 0.685714 | 0.558333 | 0.063006 | 0.05818 | 3 | 13 |
| 52 | OTU_146 | *Rhodobacteraceae* | 17 | 0.742647 | 0.57265 | 0.054962 | 0.07114 | 2 | 14 |
| 53 | OTU_54 | Ellin6529 | 11 | 0.490909 | 0.531746 | 0.058218 | 0.03876 | 3 | 10 |
| 54 | OTU_87 | *Aeromonas* | 17 | 0.720588 | 0.567797 | 0.051422 | 0.069632 | 3 | 14 |
| 55 | OTU_15 | *Anaerolineae* | 17 | 0.654412 | 0.57265 | 0.061921 | 0.065495 | 2 | 14 |
| 56 | OTU_20 | Ellin515 | 9 | 0.527778 | 0.515385 | 0.028264 | 0.032581 | 3 | 9 |
| 57 | OTU_28 | *Hydrogenophaga* | 14 | 0.813187 | 0.553719 | 0.030003 | 0.059664 | 3 | 12 |
| 58 | OTU_55 | *Syntrophobacteraceae* | 12 | 1 | 0.54918 | 0 | 0.056916 | 2 | 12 |
| 59 | OTU_78 | pGrfC26 | 10 | 0.533333 | 0.540323 | 0.064024 | 0.036 | 2 | 9 |
| 60 | OTU_80 | AKYG1722 | 11 | 0.745455 | 0.531746 | 0.025929 | 0.045416 | 3 | 10 |
| 61 | OTU_96 | LCP-6 | 17 | 0.669118 | 0.57265 | 0.080334 | 0.067057 | 2 | 14 |
| 62 | OTU_1 | *Sulfuricurvum* | 6 | 0.4 | 0.489051 | 0.027789 | 0.020697 | 3 | 6 |
| 63 | OTU_11 | envOPS12 | 10 | 0.666667 | 0.531746 | 0.031694 | 0.040018 | 3 | 10 |
| 64 | OTU_193 | WD2101 | 11 | 0.636364 | 0.536 | 0.042132 | 0.042917 | 3 | 10 |
| 65 | OTU_47 | *Acidimicrobiales* | 13 | 0.602564 | 0.553719 | 0.048438 | 0.047685 | 2 | 12 |
| 66 | OTU_3 | *Anaerolineae* | 14 | 0.538462 | 0.54918 | 0.072633 | 0.04843 | 3 | 12 |
| 67 | OTU_37 | *Rhizobiales* | 11 | 0.836364 | 0.540323 | 0.017602 | 0.04863 | 3 | 10 |
| 68 | OTU_9 | SJA-15 | 19 | 0.573099 | 0.582609 | 0.093563 | 0.070168 | 2 | 14 |

Table S7. Estimation of network property of co-occurrence microbial network of rice cultivar TKM13

| Node_Index | Label | Taxa | Degree | Cluster_Coeff | Closeness | Betweenness | Eigen_Vector | Eccentricity | Coreness |
| --- | --- | --- | --- | --- | --- | --- | --- | --- | --- |
| 1 | ArcOTU_23 | *Parvarchaea* | 43 | 0.632337 | 0.736264 | 0.699564 | 0.166922 | 2 | 30 |
| 2 | ArcOTU_12 | *Methanosaeta* | 4 | 1 | 0.485507 | 0 | 0.019541 | 3 | 4 |
| 3 | OTU_159 | *Spirochaetales* | 44 | 0.617336 | 0.744444 | 0.76794 | 0.168748 | 2 | 30 |
| 4 | OTU_21 | *Clostridiaceae* | 42 | 0.666667 | 0.728261 | 0.527992 | 0.167679 | 2 | 30 |
| 5 | OTU_84 | *Luteolibacter* | 47 | 0.568918 | 0.770115 | 1 | 0.173706 | 2 | 30 |
| 6 | ArcOTU_2 | *Crenarchaeota* | 38 | 0.766714 | 0.697917 | 0.247372 | 0.161894 | 2 | 30 |
| 7 | ArcOTU_6 | *Methanobacterium* | 40 | 0.726923 | 0.712766 | 0.284801 | 0.166139 | 2 | 30 |
| 8 | ArcOTU_7 | *Methanocella* | 41 | 0.668293 | 0.72043 | 0.595055 | 0.163656 | 2 | 30 |
| 9 | ArcOTU_8 | *Methanomicrobiales* | 40 | 0.721795 | 0.712766 | 0.289115 | 0.165369 | 2 | 30 |
| 10 | OTU_1 | *Sulfuricurvum* | 14 | 0.791209 | 0.54918 | 0.028487 | 0.060829 | 3 | 13 |
| 11 | OTU_101 | *Chloroflexi* | 37 | 0.783784 | 0.690722 | 0.20532 | 0.159337 | 2 | 30 |
| 12 | OTU_121 | *Sinobacteraceae* | 43 | 0.655592 | 0.736264 | 0.51691 | 0.169401 | 2 | 30 |
| 13 | OTU_125 | *Myxococcales* | 46 | 0.593237 | 0.761364 | 0.747742 | 0.173283 | 2 | 30 |
| 14 | OTU_129 | *Desulfobacteraceae* | 47 | 0.588344 | 0.770115 | 0.737741 | 0.175663 | 2 | 30 |
| 15 | OTU_137 | *Lachnospiraceae* | 43 | 0.65227 | 0.736264 | 0.476804 | 0.169583 | 2 | 30 |
| 16 | OTU_146 | *Rhodobacteraceae* | 42 | 0.67712 | 0.728261 | 0.37811 | 0.168355 | 2 | 30 |
| 17 | OTU_151 | 44159 | 39 | 0.719298 | 0.705263 | 0.28164 | 0.161341 | 2 | 30 |
| 18 | OTU_189 | *Acidobacteria-6* | 40 | 0.728205 | 0.712766 | 0.264637 | 0.166165 | 2 | 30 |
| 19 | OTU_193 | WD2101 | 37 | 0.723724 | 0.690722 | 0.276225 | 0.153416 | 2 | 29 |
| 20 | OTU_2 | OD1 | 7 | 0.761905 | 0.515385 | 0.006286 | 0.028087 | 3 | 7 |
| 21 | OTU_22 | *Chitinophagaceae* | 39 | 0.726046 | 0.705263 | 0.315274 | 0.161843 | 2 | 30 |
| 22 | OTU_24 | *Rhodocyclaceae* | 14 | 0.802198 | 0.558333 | 0.032123 | 0.061081 | 2 | 13 |
| 23 | OTU_25 | JG30-KF-CM45 | 22 | 0.701299 | 0.598214 | 0.094345 | 0.090208 | 2 | 17 |
| 24 | OTU_28 | *Hydrogenophaga* | 38 | 0.758179 | 0.697917 | 0.240749 | 0.160617 | 2 | 30 |
| 25 | OTU_37 | *Rhizobiales* | 42 | 0.666667 | 0.728261 | 0.464392 | 0.167582 | 2 | 30 |
| 26 | OTU_428 | BPC076 | 40 | 0.723077 | 0.712766 | 0.292312 | 0.165298 | 2 | 30 |
| 27 | OTU_44 | *Aeromonadaceae* | 44 | 0.615222 | 0.744444 | 0.585371 | 0.169304 | 2 | 30 |
| 28 | OTU_48 | DS-18 | 39 | 0.751687 | 0.705263 | 0.209497 | 0.164602 | 2 | 30 |
| 29 | OTU_50 | *Bacteroidales* | 15 | 0.504762 | 0.563025 | 0.094601 | 0.053564 | 2 | 13 |
| 30 | OTU_55 | *Syntrophobacteraceae* | 22 | 0.536797 | 0.598214 | 0.243065 | 0.07858 | 2 | 14 |
| 31 | OTU_58 | Ellin6075 | 45 | 0.60202 | 0.752809 | 0.767701 | 0.170808 | 2 | 30 |
| 32 | OTU_61 | *Bacteroidales* | 45 | 0.618182 | 0.752809 | 0.613052 | 0.172847 | 2 | 30 |
| Node_Index | Label | Taxa | Degree | Cluster_Coeff | Closeness | Betweenness | Eigen_Vector | Eccentricity | Coreness |
| 33 | OTU_72 | *Cyanobacteria* | 47 | 0.577243 | 0.770115 | 0.884121 | 0.174616 | 2 | 30 |
| 34 | OTU_77 | *Bacillales* | 40 | 0.716667 | 0.712766 | 0.339472 | 0.164523 | 2 | 30 |
| 35 | OTU_78 | pGrfC26 | 35 | 0.833613 | 0.676768 | 0.163793 | 0.155231 | 2 | 30 |
| 36 | OTU_83 | *Actinobacteria* | 45 | 0.59899 | 0.752809 | 0.741085 | 0.170532 | 2 | 30 |
| 37 | OTU_9 | SJA-15 | 15 | 0.809524 | 0.563025 | 0.018162 | 0.066348 | 2 | 14 |
| 38 | OTU_91 | *Peredibacter* | 44 | 0.639535 | 0.744444 | 0.537817 | 0.172032 | 2 | 30 |
| 39 | OTU_95 | *Algoriphagus* | 40 | 0.705128 | 0.712766 | 0.360013 | 0.163982 | 2 | 30 |
| 40 | OTU_96 | LCP-6 | 41 | 0.708537 | 0.72043 | 0.305763 | 0.168131 | 2 | 30 |
| 41 | ArcOTU_3 | *Candidatus Nitrososphaera* | 11 | 0.490909 | 0.540323 | 0.037413 | 0.0387 | 3 | 11 |
| 42 | OTU_140 | *Koribacteraceae* | 11 | 0.563636 | 0.540323 | 0.040172 | 0.041186 | 3 | 10 |
| 43 | OTU_154 | C0119 | 16 | 0.716667 | 0.567797 | 0.068533 | 0.067187 | 2 | 13 |
| 44 | OTU_168 | OPB54 | 16 | 0.558333 | 0.567797 | 0.112428 | 0.059064 | 2 | 14 |
| 45 | OTU_187 | EB1017 | 11 | 0.581818 | 0.536 | 0.05367 | 0.039462 | 3 | 10 |
| 46 | OTU_20 | Ellin515 | 14 | 0.67033 | 0.553719 | 0.039012 | 0.056208 | 3 | 13 |
| 47 | OTU_60 | *Clostridium* | 11 | 0.690909 | 0.540323 | 0.019962 | 0.047039 | 3 | 11 |
| 48 | OTU_87 | *Aeromonas* | 21 | 0.452381 | 0.59292 | 0.277473 | 0.068716 | 2 | 14 |
| 49 | OTU_89 | S035 | 11 | 0.6 | 0.544715 | 0.034762 | 0.04119 | 2 | 10 |
| 50 | ArcOTU_24 | *Parvarchaea* | 8 | 0.678571 | 0.523438 | 0.009211 | 0.032226 | 3 | 8 |
| 51 | OTU_109 | *Desulfobulbaceae* | 16 | 0.65 | 0.563025 | 0.084402 | 0.062896 | 3 | 13 |
| 52 | OTU_10 | *Saprospiraceae* | 13 | 0.564103 | 0.553719 | 0.050917 | 0.048052 | 2 | 11 |
| 53 | OTU_124 | *Gaiellaceae* | 16 | 0.633333 | 0.567797 | 0.096336 | 0.063762 | 2 | 13 |
| 54 | OTU_80 | AKYG1722 | 16 | 0.566667 | 0.563025 | 0.071191 | 0.061869 | 3 | 13 |
| 55 | OTU_15 | *Anaerolineae* | 10 | 0.533333 | 0.51938 | 0.038027 | 0.034779 | 3 | 10 |
| 56 | OTU_45 | *Methylosinus* | 17 | 0.691176 | 0.567797 | 0.06283 | 0.069261 | 3 | 14 |
| 57 | OTU_54 | Ellin6529 | 22 | 0.515152 | 0.598214 | 0.255216 | 0.077017 | 2 | 14 |
| 58 | OTU_57 | *Geobacteraceae* | 15 | 0.542857 | 0.558333 | 0.077812 | 0.0539 | 3 | 13 |
| 59 | OTU_11 | envOPS12 | 14 | 0.747253 | 0.558333 | 0.029367 | 0.058552 | 2 | 13 |
| 60 | OTU_32 | Gemm-5 | 16 | 0.791667 | 0.567797 | 0.028995 | 0.068091 | 2 | 14 |
| 61 | OTU_42 | *Kouleothrixaceae* | 12 | 0.893939 | 0.54918 | 0.006208 | 0.05503 | 2 | 12 |
| 62 | OTU_47 | *Acidimicrobiales* | 8 | 0.25 | 0.515385 | 0.036716 | 0.021367 | 3 | 8 |
| 63 | OTU_6 | *Anaerolinea* | 16 | 0.633333 | 0.567797 | 0.103088 | 0.060264 | 2 | 13 |
| 64 | OTU_16 | *Geobacter* | 11 | 0.581818 | 0.536 | 0.042007 | 0.042533 | 3 | 10 |
| 65 | OTU_49 | *Rhodospirillales* | 13 | 0.653846 | 0.544715 | 0.034474 | 0.051597 | 3 | 13 |
| 66 | OTU_299 | *Pseudoxanthomonas* | 13 | 0.794872 | 0.540323 | 0.019678 | 0.055978 | 3 | 13 |
| 67 | OTU_185 | *Candidatus Entotheonella* | 9 | 0.527778 | 0.527559 | 0.041832 | 0.030434 | 3 | 9 |
| 68 | OTU_3 | *Anaerolineae* | 11 | 0.636364 | 0.540323 | 0.02315 | 0.044728 | 3 | 11 |

Table S8. Estimation of network property of co-occurrence microbial network of rice cultivar HUR105.

| Node_Index | Label | Taxa | Degree | Cluster_Coeff | Closeness | Betweenness | Eigen_Vector | Eccentricity | Coreness |
| --- | --- | --- | --- | --- | --- | --- | --- | --- | --- |
| 1 | ArcOTU_23 | *Parvarchaea* | 43 | 0.668882 | 0.736264 | 0.441021 | 0.167826 | 2 | 31 |
| 2 | ArcOTU_12 | *Methanosaeta* | 10 | 0.666667 | 0.51938 | 0.014644 | 0.038163 | 3 | 10 |
| 3 | ArcOTU_3 | *Candidatus Nitrososphaera* | 46 | 0.606763 | 0.761364 | 0.631264 | 0.171761 | 2 | 31 |
| 4 | OTU_124 | *Gaiellaceae* | 44 | 0.655391 | 0.744444 | 0.376557 | 0.170223 | 2 | 31 |
| 5 | OTU_140 | *Koribacteraceae* | 42 | 0.680604 | 0.728261 | 0.425659 | 0.165404 | 2 | 31 |
| 6 | OTU_187 | EB1017 | 19 | 0.643275 | 0.57265 | 0.087137 | 0.073328 | 3 | 14 |
| 7 | OTU_189 | *Acidobacteria-6* | 39 | 0.738192 | 0.705263 | 0.207347 | 0.159941 | 2 | 31 |
| 8 | OTU_299 | *Pseudoxanthomonas* | 40 | 0.724359 | 0.712766 | 0.292542 | 0.162319 | 2 | 31 |
| 9 | OTU_42 | *Kouleothrixaceae* | 43 | 0.6567 | 0.736264 | 0.494159 | 0.166534 | 2 | 31 |
| 10 | OTU_60 | *Clostridium* | 17 | 0.544118 | 0.553719 | 0.068977 | 0.062336 | 3 | 14 |
| 11 | OTU_77 | *Bacillales* | 10 | 0.688889 | 0.531746 | 0.013551 | 0.040735 | 3 | 10 |
| 12 | ArcOTU_24 | *Parvarchaea* | 13 | 0.602564 | 0.54918 | 0.03446 | 0.049344 | 3 | 11 |
| 13 | ArcOTU_2 | *Crenarchaeota* | 12 | 0.424242 | 0.527559 | 0.077702 | 0.039433 | 3 | 10 |
| 14 | ArcOTU_6 | *Methanobacterium* | 13 | 0.820513 | 0.54918 | 0.016753 | 0.055507 | 3 | 12 |
| 15 | OTU_1 | *Sulfuricurvum* | 4 | 0.666667 | 0.482014 | 0.002297 | 0.015223 | 3 | 4 |
| 16 | OTU_109 | *Desulfobulbaceae* | 15 | 0.685714 | 0.563025 | 0.040463 | 0.061183 | 2 | 13 |
| 17 | OTU_154 | C0119 | 42 | 0.691057 | 0.728261 | 0.331255 | 0.166367 | 2 | 31 |
| 18 | OTU_168 | OPB54 | 42 | 0.69338 | 0.728261 | 0.298268 | 0.167109 | 2 | 31 |
| 19 | OTU_193 | WD2101 | 44 | 0.660677 | 0.744444 | 0.355179 | 0.170845 | 2 | 31 |
| 20 | OTU_45 | *Methylosinus* | 42 | 0.671312 | 0.728261 | 0.467026 | 0.164597 | 2 | 31 |
| 21 | OTU_54 | Ellin6529 | 42 | 0.689895 | 0.728261 | 0.330986 | 0.166721 | 2 | 31 |
| 22 | OTU_57 | *Geobacteraceae* | 10 | 0.533333 | 0.5 | 0.029623 | 0.03403 | 3 | 9 |
| 23 | ArcOTU_8 | *Methanomicrobiales* | 11 | 0.836364 | 0.523438 | 0.007191 | 0.049354 | 3 | 11 |
| 24 | OTU_101 | *Chloroflexi* | 39 | 0.775978 | 0.705263 | 0.144642 | 0.163462 | 2 | 31 |
| 25 | OTU_11 | envOPS12 | 19 | 0.77193 | 0.582609 | 0.036888 | 0.079823 | 2 | 16 |
| 26 | OTU_121 | *Sinobacteraceae* | 43 | 0.675526 | 0.736264 | 0.296496 | 0.169385 | 2 | 31 |
| 27 | OTU_125 | *Myxococcales* | 44 | 0.663848 | 0.744444 | 0.354049 | 0.171471 | 2 | 31 |
| 28 | OTU_129 | *Desulfobacteraceae* | 41 | 0.713415 | 0.72043 | 0.284713 | 0.165085 | 2 | 31 |
| 29 | OTU_137 | *Lachnospiraceae* | 39 | 0.761134 | 0.705263 | 0.202266 | 0.162146 | 2 | 31 |
| 30 | OTU_146 | *Rhodobacteraceae* | 41 | 0.717073 | 0.72043 | 0.272452 | 0.165547 | 2 | 31 |
| 31 | OTU_15 | *Anaerolineae* | 5 | 0.5 | 0.482014 | 0.005096 | 0.017182 | 3 | 5 |
| 32 | OTU_151 | 44159 | 39 | 0.761134 | 0.705263 | 0.218238 | 0.16207 | 2 | 31 |
| 33 | OTU_16 | *Geobacter* | 12 | 0.909091 | 0.544715 | 0.003651 | 0.054301 | 3 | 12 |
| 34 | OTU_185 | *Candidatus Entotheonella* | 39 | 0.77193 | 0.705263 | 0.162762 | 0.163278 | 2 | 31 |
| Node_Index | Label | Taxa | Degree | Cluster_Coeff | Closeness | Betweenness | Eigen_Vector | Eccentricity | Coreness |
| 35 | OTU_24 | *Rhodocyclaceae* | 38 | 0.759602 | 0.697917 | 0.18811 | 0.158156 | 2 | 31 |
| 36 | OTU_428 | BPC076 | 41 | 0.692683 | 0.72043 | 0.260058 | 0.163228 | 2 | 31 |
| 37 | OTU_44 | *Aeromonadaceae* | 42 | 0.700348 | 0.728261 | 0.27027 | 0.167796 | 2 | 31 |
| 38 | OTU_49 | *Rhodospirillales* | 15 | 0.761905 | 0.563025 | 0.072622 | 0.063421 | 2 | 14 |
| 39 | OTU_50 | *Bacteroidales* | 37 | 0.81982 | 0.690722 | 0.129983 | 0.159469 | 2 | 31 |
| 40 | OTU_72 | *Cyanobacteria* | 42 | 0.678281 | 0.728261 | 0.438803 | 0.165242 | 2 | 31 |
| 41 | OTU_78 | pGrfC26 | 39 | 0.753036 | 0.705263 | 0.210313 | 0.161619 | 2 | 31 |
| 42 | OTU_80 | AKYG1722 | 45 | 0.636364 | 0.752809 | 0.646423 | 0.171683 | 2 | 31 |
| 43 | OTU_84 | *Luteolibacter* | 44 | 0.653277 | 0.744444 | 0.445194 | 0.169868 | 2 | 31 |
| 44 | OTU_87 | *Aeromonas* | 39 | 0.762483 | 0.705263 | 0.175172 | 0.162512 | 2 | 31 |
| 45 | OTU_89 | S035 | 38 | 0.799431 | 0.697917 | 0.124111 | 0.161693 | 2 | 31 |
| 46 | OTU_91 | *Peredibacter* | 41 | 0.713415 | 0.72043 | 0.257364 | 0.165719 | 2 | 31 |
| 47 | OTU_95 | *Algoriphagus* | 51 | 0.532549 | 0.807229 | 1 | 0.178732 | 2 | 31 |
| 48 | OTU_96 | LCP-6 | 45 | 0.584848 | 0.752809 | 0.825624 | 0.165405 | 2 | 31 |
| 49 | OTU_2 | OD1 | 11 | 0.545455 | 0.536 | 0.050011 | 0.038628 | 3 | 10 |
| 50 | OTU_32 | Gemm-5 | 18 | 0.705882 | 0.567797 | 0.05559 | 0.072528 | 3 | 14 |
| 51 | ArcOTU_7 | *Methanocella* | 18 | 0.581699 | 0.577586 | 0.077867 | 0.06371 | 2 | 14 |
| 52 | OTU_21 | *Clostridiaceae* | 19 | 0.614035 | 0.582609 | 0.146822 | 0.0718 | 2 | 14 |
| 53 | OTU_3 | *Anaerolineae* | 9 | 0.666667 | 0.531746 | 0.009296 | 0.034409 | 3 | 9 |
| 54 | OTU_48 | DS-18 | 15 | 0.857143 | 0.563025 | 0.012036 | 0.065833 | 2 | 14 |
| 55 | OTU_58 | Ellin6075 | 8 | 1 | 0.51938 | 0 | 0.037832 | 3 | 8 |
| 56 | OTU_61 | *Bacteroidales* | 14 | 0.934066 | 0.54918 | 0.004367 | 0.063382 | 3 | 13 |
| 57 | OTU_9 | SJA-15 | 15 | 0.704762 | 0.553719 | 0.054898 | 0.058744 | 3 | 14 |
| 58 | OTU_159 | *Spirochaetales* | 13 | 0.705128 | 0.54918 | 0.021355 | 0.052817 | 3 | 12 |
| 59 | OTU_28 | *Hydrogenophaga* | 16 | 0.65 | 0.563025 | 0.097685 | 0.061917 | 3 | 14 |
| 60 | OTU_37 | *Rhizobiales* | 12 | 0.69697 | 0.527559 | 0.016211 | 0.048146 | 3 | 11 |
| 61 | OTU_10 | *Saprospiraceae* | 8 | 0.571429 | 0.523438 | 0.042406 | 0.029846 | 3 | 7 |
| 62 | OTU_6 | *Anaerolinea* | 5 | 0.1 | 0.468531 | 0.016413 | 0.011351 | 3 | 5 |
| 63 | OTU_20 | Ellin515 | 12 | 0.757576 | 0.540323 | 0.018433 | 0.050504 | 3 | 11 |
| 64 | OTU_22 | *Chitinophagaceae* | 11 | 0.745455 | 0.540323 | 0.011387 | 0.046132 | 3 | 11 |
| 65 | OTU_83 | *Actinobacteria* | 11 | 1 | 0.536 | 0 | 0.052839 | 3 | 11 |
| 66 | OTU_47 | *Acidimicrobiales* | 11 | 0.854545 | 0.527559 | 0.007013 | 0.048046 | 3 | 11 |
| 67 | OTU_55 | *Syntrophobacteraceae* | 3 | 1 | 0.485507 | 0 | 0.014507 | 3 | 3 |
| 68 | OTU_25 | JG30-KF-CM45 | 6 | 0.8 | 0.492647 | 0.001888 | 0.025616 | 3 | 6 |

Table S9. Estimation of network property of co-occurrence microbial network of rice cultivar CO52.

| Node Index | Label | Taxa | Degree | Cluster_Coeff | Closeness | Betweenness | Eigen_Vector | Eccentricity | Coreness |
| --- | --- | --- | --- | --- | --- | --- | --- | --- | --- |
| 1 | ArcOTU_2 | *Crenarchaeota* | 6 | 0.733333 | 0.492647 | 0.00589 | 0.028477 | 3 | 6 |
| 2 | ArcOTU_12 | *Methanosaeta* | 19 | 0.614035 | 0.577586 | 0.202087 | 0.087995 | 3 | 14 |
| 3 | OTU_10 | *Saprospiraceae* | 6 | 0.266667 | 0.485507 | 0.016052 | 0.021326 | 3 | 6 |
| 4 | OTU_124 | *Gaiellaceae* | 44 | 0.51797 | 0.744444 | 1 | 0.190128 | 2 | 26 |
| 5 | OTU_140 | *Koribacteraceae* | 40 | 0.558974 | 0.712766 | 0.835332 | 0.18015 | 2 | 26 |
| 6 | OTU_146 | *Rhodobacteraceae* | 39 | 0.603239 | 0.705263 | 0.554886 | 0.181489 | 2 | 26 |
| 7 | OTU_159 | *Spirochaetales* | 42 | 0.555168 | 0.728261 | 0.780791 | 0.187494 | 2 | 26 |
| 8 | OTU_168 | OPB54 | 39 | 0.589744 | 0.705263 | 0.723823 | 0.179306 | 2 | 26 |
| 9 | OTU_189 | *Acidobacteria-6* | 40 | 0.561538 | 0.712766 | 0.796941 | 0.179406 | 2 | 26 |
| 10 | OTU_193 | WD2101 | 37 | 0.647147 | 0.690722 | 0.394283 | 0.177493 | 2 | 26 |
| 11 | OTU_2 | OD1 | 7 | 0.952381 | 0.507576 | 0.000988 | 0.038354 | 3 | 7 |
| 12 | OTU_22 | *Chitinophagaceae* | 34 | 0.7041 | 0.67 | 0.34795 | 0.169987 | 2 | 26 |
| 13 | OTU_37 | *Rhizobiales* | 17 | 0.529412 | 0.57265 | 0.107676 | 0.074426 | 2 | 12 |
| 14 | OTU_44 | *Aeromonadaceae* | 15 | 0.4 | 0.563025 | 0.118677 | 0.059366 | 2 | 12 |
| 15 | OTU_45 | *Methylosinus* | 43 | 0.531561 | 0.736264 | 0.955858 | 0.187886 | 2 | 26 |
| 16 | OTU_58 | Ellin6075 | 40 | 0.565385 | 0.712766 | 0.761777 | 0.180061 | 2 | 26 |
| 17 | OTU_80 | AKYG1722 | 39 | 0.595142 | 0.705263 | 0.62707 | 0.17956 | 2 | 26 |
| 18 | OTU_89 | S035 | 38 | 0.613087 | 0.697917 | 0.624557 | 0.178184 | 2 | 26 |
| 19 | OTU_95 | *Algoriphagus* | 39 | 0.596491 | 0.705263 | 0.582347 | 0.179911 | 2 | 26 |
| 20 | OTU_96 | LCP-6 | 21 | 0.52381 | 0.59292 | 0.270864 | 0.091133 | 2 | 14 |
| 21 | OTU_47 | *Acidimicrobiales* | 13 | 0.448718 | 0.54918 | 0.086272 | 0.057228 | 3 | 12 |
| 22 | OTU_109 | *Desulfobulbaceae* | 12 | 0.454545 | 0.536 | 0.060573 | 0.051963 | 3 | 11 |
| 23 | ArcOTU_23 | *Parvarchaea* | 13 | 0.871795 | 0.553719 | 0.0144 | 0.072759 | 2 | 12 |
| 24 | OTU_151 | 44159 | 39 | 0.588394 | 0.705263 | 0.658574 | 0.178782 | 2 | 26 |
| 25 | OTU_185 | *Candidatus Entotheonella* | 38 | 0.604552 | 0.697917 | 0.53964 | 0.177146 | 2 | 26 |
| 26 | OTU_48 | DS-18 | 40 | 0.566667 | 0.712766 | 0.764968 | 0.180799 | 2 | 26 |
| 27 | OTU_50 | *Bacteroidales* | 40 | 0.553846 | 0.712766 | 0.887453 | 0.179035 | 2 | 26 |
| 28 | OTU_54 | Ellin6529 | 38 | 0.590327 | 0.697917 | 0.743211 | 0.17474 | 2 | 26 |
| 29 | OTU_61 | *Bacteroidales* | 36 | 0.663492 | 0.683673 | 0.33047 | 0.175372 | 2 | 26 |
| 30 | OTU_72 | *Cyanobacteria* | 38 | 0.608819 | 0.697917 | 0.535122 | 0.17742 | 2 | 26 |
| 31 | ArcOTU_24 | *Parvarchaea* | 4 | 1 | 0.478571 | 0 | 0.02389 | 3 | 4 |
| 32 | OTU_78 | pGrfC26 | 38 | 0.600285 | 0.697917 | 0.63165 | 0.177056 | 2 | 26 |
| 33 | ArcOTU_3 | *Candidatus Nitrososphaera* | 33 | 0.723485 | 0.663366 | 0.255587 | 0.167039 | 2 | 26 |
| 34 | OTU_129 | *Desulfobacteraceae* | 12 | 0.530303 | 0.527559 | 0.048525 | 0.053917 | 3 | 11 |
| Node Index | Label | Taxa | Degree | Cluster_Coeff | Closeness | Betweenness | Eigen_Vector | Eccentricity | Coreness |
| 35 | OTU_15 | *Anaerolineae* | 38 | 0.59744 | 0.697917 | 0.598545 | 0.176297 | 2 | 26 |
| 36 | OTU_187 | EB1017 | 15 | 0.619048 | 0.553719 | 0.077978 | 0.070796 | 3 | 11 |
| 37 | OTU_28 | *Hydrogenophaga* | 11 | 0.781818 | 0.544715 | 0.019204 | 0.056835 | 2 | 10 |
| 38 | OTU_32 | Gemm-5 | 9 | 0.861111 | 0.527559 | 0.005546 | 0.049108 | 3 | 9 |
| 39 | OTU_42 | *Kouleothrixaceae* | 11 | 0.636364 | 0.540323 | 0.055654 | 0.052064 | 3 | 10 |
| 40 | OTU_428 | BPC076 | 39 | 0.587045 | 0.705263 | 0.664653 | 0.178867 | 2 | 26 |
| 41 | OTU_55 | *Syntrophobacteraceae* | 36 | 0.646032 | 0.683673 | 0.514451 | 0.173033 | 2 | 26 |
| 42 | OTU_84 | *Luteolibacter* | 33 | 0.719697 | 0.663366 | 0.275511 | 0.16719 | 2 | 26 |
| 43 | OTU_87 | *Aeromonas* | 37 | 0.642643 | 0.690722 | 0.391437 | 0.177509 | 2 | 26 |
| 44 | OTU_9 | SJA-15 | 11 | 0.690909 | 0.536 | 0.034079 | 0.055856 | 3 | 10 |
| 45 | ArcOTU_6 | *Methanobacterium* | 8 | 0.857143 | 0.51145 | 0.004763 | 0.042801 | 3 | 8 |
| 46 | ArcOTU_7 | *Methanocella* | 4 | 1 | 0.478571 | 0 | 0.023729 | 3 | 4 |
| 47 | OTU_1 | *Sulfuricurvum* | 16 | 0.466667 | 0.558333 | 0.128861 | 0.066947 | 3 | 12 |
| 48 | ArcOTU_8 | *Methanomicrobiales* | 16 | 0.558333 | 0.558333 | 0.088483 | 0.072815 | 3 | 12 |
| 49 | OTU_137 | *Lachnospiraceae* | 17 | 0.551471 | 0.567797 | 0.111556 | 0.074055 | 3 | 12 |
| 50 | OTU_25 | JG30-KF-CM45 | 13 | 0.75641 | 0.54918 | 0.022444 | 0.066496 | 3 | 12 |
| 51 | OTU_299 | *Pseudoxanthomonas* | 12 | 0.545455 | 0.536 | 0.050608 | 0.049733 | 3 | 11 |
| 52 | OTU_57 | *Geobacteraceae* | 17 | 0.580882 | 0.57265 | 0.105056 | 0.076912 | 2 | 12 |
| 53 | OTU_60 | *Clostridium* | 14 | 0.516484 | 0.558333 | 0.070407 | 0.063806 | 2 | 12 |
| 54 | OTU_121 | *Sinobacteraceae* | 7 | 0.714286 | 0.5 | 0.007325 | 0.034362 | 3 | 7 |
| 55 | OTU_125 | *Myxococcales* | 15 | 0.638095 | 0.563025 | 0.052747 | 0.068482 | 2 | 12 |
| 56 | OTU_21 | *Clostridiaceae* | 15 | 0.657143 | 0.553719 | 0.063015 | 0.072852 | 3 | 12 |
| 57 | OTU_101 | *Chloroflexi* | 16 | 0.475 | 0.567797 | 0.17384 | 0.0667 | 2 | 12 |
| 58 | OTU_83 | *Actinobacteria* | 10 | 0.6 | 0.536 | 0.042037 | 0.047127 | 3 | 9 |
| 59 | OTU_11 | envOPS12 | 6 | 1 | 0.496296 | 0 | 0.035246 | 3 | 6 |
| 60 | OTU_20 | Ellin515 | 13 | 0.74359 | 0.54918 | 0.025622 | 0.066223 | 3 | 12 |
| 61 | OTU_3 | *Anaerolineae* | 10 | 0.6 | 0.51938 | 0.022461 | 0.048371 | 3 | 10 |
| 62 | OTU_6 | *Anaerolinea* | 9 | 0.75 | 0.531746 | 0.011833 | 0.04706 | 3 | 9 |
| 63 | OTU_91 | *Peredibacter* | 17 | 0.588235 | 0.57265 | 0.104774 | 0.079977 | 2 | 12 |
| 64 | OTU_77 | *Bacillales* | 12 | 0.651515 | 0.544715 | 0.03315 | 0.060193 | 3 | 11 |
| 65 | OTU_49 | *Rhodospirillales* | 10 | 0.777778 | 0.523438 | 0.012874 | 0.052055 | 3 | 10 |
| 66 | OTU_154 | C0119 | 8 | 0.321429 | 0.51145 | 0.031728 | 0.03237 | 3 | 8 |
| 67 | OTU_16 | *Geobacter* | 11 | 0.654545 | 0.527559 | 0.024235 | 0.051664 | 3 | 11 |
| 68 | OTU_24 | *Rhodocyclaceae* | 13 | 0.589744 | 0.553719 | 0.042238 | 0.061684 | 2 | 12 |

Table S10. Estimation of network property of co-occurrence microbial network of rice cultivar Rajendra Sweta

| Node_Index | Label | Taxa | Degree | Cluster_Coeff | Closeness | Betweenness | Eigen_Vector | Eccentricity | Coreness |
| --- | --- | --- | --- | --- | --- | --- | --- | --- | --- |
| 1 | ArcOTU_2 | *Crenarchaeota* | 11 | 0.363636 | 0.515385 | 0.092756 | 0.039587 | 3 | 9 |
| 2 | ArcOTU_12 | *Methanosaeta* | 12 | 0.666667 | 0.536 | 0.049147 | 0.058257 | 3 | 10 |
| 3 | OTU_109 | *Desulfobulbaceae* | 39 | 0.603239 | 0.705263 | 0.417953 | 0.18336 | 2 | 26 |
| 4 | OTU_154 | C0119 | 36 | 0.647619 | 0.683673 | 0.369542 | 0.175314 | 2 | 26 |
| 5 | OTU_16 | *Geobacter* | 5 | 0.6 | 0.482014 | 0.004253 | 0.025544 | 3 | 5 |
| 6 | OTU_168 | OPB54 | 39 | 0.581646 | 0.705263 | 0.506466 | 0.180291 | 2 | 26 |
| 7 | OTU_193 | WD2101 | 38 | 0.624467 | 0.697917 | 0.363849 | 0.181053 | 2 | 26 |
| 8 | OTU_22 | *Chitinophagaceae* | 13 | 0.589744 | 0.553719 | 0.053485 | 0.061126 | 2 | 10 |
| 9 | OTU_428 | BPC076 | 39 | 0.595142 | 0.705263 | 0.4568 | 0.182084 | 2 | 26 |
| 10 | OTU_50 | *Bacteroidales* | 40 | 0.569231 | 0.712766 | 0.564511 | 0.182634 | 2 | 26 |
| 11 | OTU_78 | pGrfC26 | 34 | 0.705882 | 0.67 | 0.239689 | 0.172538 | 2 | 26 |
| 12 | OTU_87 | *Aeromonas* | 44 | 0.506342 | 0.744444 | 1 | 0.189957 | 2 | 26 |
| 13 | OTU_96 | LCP-6 | 37 | 0.644144 | 0.690722 | 0.409456 | 0.178757 | 2 | 26 |
| 14 | OTU_187 | EB1017 | 40 | 0.610256 | 0.712766 | 0.369162 | 0.188639 | 2 | 26 |
| 15 | OTU_21 | *Clostridiaceae* | 11 | 0.654545 | 0.527559 | 0.033704 | 0.052844 | 3 | 9 |
| 16 | OTU_28 | *Hydrogenophaga* | 9 | 0.361111 | 0.492647 | 0.041039 | 0.032699 | 3 | 8 |
| 17 | OTU_3 | *Anaerolineae* | 6 | 0.4 | 0.503759 | 0.038943 | 0.023516 | 3 | 5 |
| 18 | OTU_54 | Ellin6529 | 14 | 0.483516 | 0.553719 | 0.097284 | 0.055935 | 3 | 10 |
| 19 | OTU_61 | *Bacteroidales* | 39 | 0.597841 | 0.705263 | 0.474336 | 0.182233 | 2 | 26 |
| 20 | OTU_140 | *Koribacteraceae* | 41 | 0.541463 | 0.72043 | 0.726431 | 0.182121 | 2 | 26 |
| 21 | ArcOTU_23 | *Parvarchaea* | 7 | 0.761905 | 0.51145 | 0.005481 | 0.037372 | 3 | 7 |
| 22 | OTU_146 | *Rhodobacteraceae* | 41 | 0.562195 | 0.72043 | 0.706016 | 0.185818 | 2 | 26 |
| 23 | OTU_151 | 44159 | 37 | 0.623123 | 0.690722 | 0.431956 | 0.176252 | 2 | 26 |
| 24 | OTU_159 | *Spirochaetales* | 41 | 0.540244 | 0.72043 | 0.867937 | 0.182321 | 2 | 26 |
| 25 | OTU_25 | JG30-KF-CM45 | 11 | 0.763636 | 0.527559 | 0.01816 | 0.057349 | 3 | 10 |
| 26 | OTU_44 | *Aeromonadaceae* | 32 | 0.737903 | 0.656863 | 0.245455 | 0.164917 | 2 | 26 |
| 27 | ArcOTU_24 | *Parvarchaea* | 10 | 0.555556 | 0.515385 | 0.03547 | 0.044411 | 3 | 9 |
| 28 | OTU_189 | *Acidobacteria-6* | 42 | 0.550523 | 0.728261 | 0.740678 | 0.18874 | 2 | 26 |
| 29 | OTU_49 | *Rhodospirillales* | 11 | 0.654545 | 0.540323 | 0.05543 | 0.052434 | 3 | 9 |
| 30 | OTU_55 | *Syntrophobacteraceae* | 9 | 0.611111 | 0.51145 | 0.022732 | 0.040385 | 3 | 8 |
| 31 | OTU_57 | *Geobacteraceae* | 22 | 0.545455 | 0.59292 | 0.181075 | 0.097944 | 3 | 14 |
| 32 | OTU_77 | *Bacillales* | 39 | 0.592443 | 0.705263 | 0.427959 | 0.182038 | 2 | 26 |
| 33 | ArcOTU_3 | *Candidatus Nitrososphaera* | 17 | 0.691176 | 0.567797 | 0.082778 | 0.083027 | 3 | 14 |
| 34 | OTU_121 | *Sinobacteraceae* | 15 | 0.790476 | 0.563025 | 0.020472 | 0.080148 | 2 | 14 |
| Node_Index | Label | Taxa | Degree | Cluster_Coeff | Closeness | Betweenness | Eigen_Vector | Eccentricity | Coreness |
| 35 | OTU_2 | OD1 | 5 | 0.6 | 0.462069 | 0.006102 | 0.019201 | 3 | 5 |
| 36 | OTU_20 | Ellin515 | 12 | 0.666667 | 0.54918 | 0.023856 | 0.060472 | 2 | 11 |
| 37 | OTU_299 | *Pseudoxanthomonas* | 32 | 0.758065 | 0.656863 | 0.262817 | 0.167361 | 2 | 26 |
| 38 | OTU_48 | DS-18 | 23 | 0.458498 | 0.598214 | 0.34326 | 0.092387 | 3 | 14 |
| 39 | OTU_83 | *Actinobacteria* | 35 | 0.682353 | 0.676768 | 0.263536 | 0.174077 | 2 | 26 |
| 40 | ArcOTU_8 | *Methanomicrobiales* | 15 | 0.657143 | 0.558333 | 0.036914 | 0.072131 | 3 | 13 |
| 41 | ArcOTU_6 | *Methanobacterium* | 9 | 0.75 | 0.527559 | 0.00605 | 0.047814 | 3 | 9 |
| 42 | OTU_129 | *Desulfobacteraceae* | 40 | 0.571795 | 0.712766 | 0.611741 | 0.182979 | 2 | 26 |
| 43 | OTU_95 | *Algoriphagus* | 40 | 0.575641 | 0.712766 | 0.645763 | 0.182933 | 2 | 26 |
| 44 | ArcOTU_7 | *Methanocella* | 11 | 0.927273 | 0.540323 | 0.002489 | 0.063719 | 3 | 11 |
| 45 | OTU_60 | *Clostridium* | 33 | 0.715909 | 0.663366 | 0.240157 | 0.168156 | 2 | 26 |
| 46 | OTU_11 | OTU_11 | 12 | 0.530303 | 0.536 | 0.032539 | 0.052318 | 3 | 11 |
| 47 | OTU_137 | *Lachnospiraceae* | 37 | 0.648649 | 0.690722 | 0.39555 | 0.179123 | 2 | 26 |
| 48 | OTU_42 | *Kouleothrixaceae* | 16 | 0.691667 | 0.563025 | 0.048617 | 0.081323 | 3 | 13 |
| 49 | OTU_45 | *Methylosinus* | 18 | 0.653595 | 0.57265 | 0.06896 | 0.0859 | 3 | 14 |
| 50 | OTU_1 | *Sulfuricurvum* | 5 | 0.7 | 0.485507 | 0.005876 | 0.022837 | 3 | 5 |
| 51 | OTU_89 | S035 | 10 | 0.555556 | 0.515385 | 0.058231 | 0.044311 | 3 | 8 |
| 52 | OTU_10 | *Saprospiraceae* | 4 | 0.5 | 0.475177 | 0.007744 | 0.018646 | 3 | 4 |
| 53 | OTU_84 | *Luteolibacter* | 31 | 0.780645 | 0.650485 | 0.219809 | 0.1644 | 2 | 26 |
| 54 | OTU_101 | *Chloroflexi* | 16 | 0.933333 | 0.563025 | 0.005253 | 0.092907 | 3 | 15 |
| 55 | OTU_124 | *Gaiellaceae* | 36 | 0.668254 | 0.683673 | 0.279465 | 0.177293 | 2 | 26 |
| 56 | OTU_185 | *Candidatus Entotheonella* | 38 | 0.590327 | 0.697917 | 0.582565 | 0.176663 | 2 | 26 |
| 57 | OTU_72 | *Cyanobacteria* | 11 | 0.909091 | 0.540323 | 0.005804 | 0.062376 | 3 | 10 |
| 58 | OTU_47 | *Acidimicrobiales* | 13 | 0.717949 | 0.540323 | 0.033501 | 0.066265 | 3 | 11 |
| 59 | OTU_125 | *Myxococcales* | 11 | 0.890909 | 0.536 | 0.003949 | 0.06275 | 3 | 11 |
| 60 | OTU_58 | Ellin6075 | 14 | 0.868132 | 0.553719 | 0.026935 | 0.078651 | 3 | 13 |
| 61 | OTU_80 | AKYG1722 | 7 | 0.619048 | 0.492647 | 0.006337 | 0.034614 | 3 | 7 |
| 62 | OTU_32 | Gemm-5 | 8 | 0.857143 | 0.523438 | 0.002297 | 0.044953 | 3 | 8 |
| 63 | OTU_37 | *Rhizobiales* | 8 | 0.678571 | 0.5 | 0.009723 | 0.039482 | 3 | 8 |
| 64 | OTU_9 | SJA-15 | 12 | 0.69697 | 0.54918 | 0.021095 | 0.060811 | 2 | 10 |
| 65 | OTU_24 | *Thermoleophilia* | 3 | 1 | 0.462069 | 0 | 0.018436 | 3 | 3 |
| 66 | OTU_6 | *Anaerolinea* | 8 | 0.535714 | 0.51938 | 0.022008 | 0.036847 | 3 | 8 |
| 67 | OTU_15 | *Anaerolineae* | 6 | 0.8 | 0.496296 | 0.004274 | 0.031228 | 3 | 6 |
| 68 | OTU_91 | *Peredibacter* | 8 | 0.607143 | 0.496296 | 0.016419 | 0.036039 | 3 | 8 |

Table S11. Estimation of network property of co-occurrence microbial network of rice cultivar MTU7029

| Node_Index | Label | Taxa | Degree | Cluster_Coeff | Closeness | Betweenness | Eigen_Vector | Eccentricity | Coreness |
| --- | --- | --- | --- | --- | --- | --- | --- | --- | --- |
| 1 | ArcOTU_24 | *Parvarchaea* | 40 | 0.551282 | 0.712766 | 0.592326 | 0.193534 | 2 | 24 |
| 2 | ArcOTU_12 | *Methanosaeta* | 17 | 0.610294 | 0.57265 | 0.097075 | 0.088212 | 2 | 12 |
| 3 | ArcOTU_3 | *Candidatus Nitrososphaera* | 41 | 0.489024 | 0.72043 | 0.996191 | 0.188815 | 2 | 24 |
| 4 | ArcOTU_7 | *Methanocella* | 37 | 0.578078 | 0.690722 | 0.519719 | 0.184271 | 2 | 24 |
| 5 | OTU_121 | *Sinobacteraceae* | 37 | 0.57958 | 0.690722 | 0.561768 | 0.184735 | 2 | 24 |
| 6 | OTU_151 | 44159 | 37 | 0.558559 | 0.690722 | 0.77387 | 0.181404 | 2 | 24 |
| 7 | OTU_168 | OPB54 | 16 | 0.491667 | 0.567797 | 0.122209 | 0.075756 | 2 | 12 |
| 8 | OTU_185 | *Candidatus Entotheonella* | 34 | 0.643494 | 0.67 | 0.315783 | 0.178376 | 2 | 24 |
| 9 | OTU_189 | *Acidobacteria-6* | 40 | 0.526923 | 0.712766 | 0.722592 | 0.191482 | 2 | 24 |
| 10 | OTU_193 | WD2101 | 12 | 0.575758 | 0.540323 | 0.039246 | 0.058324 | 3 | 11 |
| 11 | OTU_24 | *Thermoleophilia* | 9 | 0.361111 | 0.51145 | 0.042693 | 0.038768 | 3 | 9 |
| 12 | OTU_49 | *Rhodospirillales* | 11 | 0.563636 | 0.540323 | 0.039163 | 0.055553 | 3 | 10 |
| 13 | OTU_50 | *Bacteroidales* | 38 | 0.544808 | 0.697917 | 0.715303 | 0.184208 | 2 | 24 |
| 14 | OTU_54 | Ellin6529 | 15 | 0.580952 | 0.563025 | 0.124772 | 0.073844 | 2 | 12 |
| 15 | OTU_55 | *Syntrophobacteraceae* | 37 | 0.591592 | 0.690722 | 0.494388 | 0.186004 | 2 | 24 |
| 16 | OTU_57 | *Geobacteraceae* | 32 | 0.719758 | 0.656863 | 0.165699 | 0.176839 | 2 | 24 |
| 17 | OTU_89 | S035 | 39 | 0.522267 | 0.705263 | 0.885847 | 0.186534 | 2 | 24 |
| 18 | OTU_96 | LCP-6 | 35 | 0.606723 | 0.676768 | 0.473967 | 0.177858 | 2 | 24 |
| 19 | ArcOTU_2 | *Crenarchaeota* | 12 | 0.439394 | 0.544715 | 0.063526 | 0.053985 | 3 | 11 |
| 20 | OTU_15 | *Anaerolineae* | 14 | 0.549451 | 0.553719 | 0.069938 | 0.068017 | 3 | 11 |
| 21 | OTU_187 | EB1017 | 15 | 0.380952 | 0.544715 | 0.148768 | 0.062455 | 3 | 11 |
| 22 | OTU_22 | *Chitinophagaceae* | 38 | 0.559033 | 0.697917 | 0.640261 | 0.185898 | 2 | 24 |
| 23 | OTU_32 | *Gemm-5* | 14 | 0.604396 | 0.54918 | 0.078049 | 0.068928 | 3 | 11 |
| 24 | OTU_428 | BPC076 | 10 | 0.511111 | 0.536 | 0.055689 | 0.04611 | 3 | 9 |
| 25 | OTU_44 | *Aeromonadaceae* | 21 | 0.533333 | 0.582609 | 0.19859 | 0.100095 | 3 | 13 |
| 26 | OTU_48 | DS-18 | 18 | 0.51634 | 0.577586 | 0.154935 | 0.08571 | 2 | 12 |
| 27 | OTU_72 | *Cyanobacteria* | 36 | 0.577778 | 0.683673 | 0.548301 | 0.179661 | 2 | 24 |
| 28 | OTU_95 | *Algoriphagus* | 42 | 0.490128 | 0.728261 | 0.95294 | 0.193202 | 2 | 24 |
| 29 | ArcOTU_23 | *Parvarchaea* | 14 | 0.571429 | 0.558333 | 0.08819 | 0.068221 | 2 | 11 |
| 30 | OTU_10 | *Saprospiraceae* | 7 | 0.47619 | 0.489051 | 0.018526 | 0.032653 | 3 | 7 |
| 31 | OTU_101 | *Chloroflexi* | 15 | 0.390476 | 0.558333 | 0.208567 | 0.06083 | 3 | 11 |
| 32 | OTU_137 | *Lachnospiraceae* | 36 | 0.588889 | 0.683673 | 0.433668 | 0.181029 | 2 | 24 |
| 33 | OTU_146 | *Rhodobacteraceae* | 35 | 0.606723 | 0.676768 | 0.486527 | 0.178623 | 2 | 24 |
| 34 | OTU_20 | Ellin515 | 13 | 0.551282 | 0.540323 | 0.056902 | 0.063819 | 3 | 11 |
| Node_Index | Label | Taxa | Degree | Cluster_Coeff | Closeness | Betweenness | Eigen_Vector | Eccentricity | Coreness |
| 35 | OTU_25 | OTU_25 | 36 | 0.588889 | 0.683673 | 0.490873 | 0.181141 | 2 | 24 |
| 36 | OTU_42 | *Kouleothrixaceae* | 17 | 0.367647 | 0.57265 | 0.16535 | 0.071264 | 2 | 11 |
| 37 | OTU_6 | *Anaerolinea* | 12 | 0.484848 | 0.540323 | 0.071942 | 0.057264 | 3 | 10 |
| 38 | OTU_83 | *Actinobacteria* | 38 | 0.541963 | 0.697917 | 0.758664 | 0.183458 | 2 | 24 |
| 39 | ArcOTU_6 | *Methanobacterium* | 14 | 0.549451 | 0.544715 | 0.072964 | 0.06855 | 3 | 11 |
| 40 | ArcOTU_8 | *Methanomicrobiales* | 39 | 0.51417 | 0.705263 | 1 | 0.184462 | 2 | 24 |
| 41 | OTU_109 | *Desulfobulbaceae* | 13 | 0.653846 | 0.553719 | 0.047723 | 0.070105 | 2 | 11 |
| 42 | OTU_124 | *Gaiellaceae* | 33 | 0.659091 | 0.663366 | 0.356009 | 0.175226 | 2 | 24 |
| 43 | OTU_154 | C0119 | 16 | 0.666667 | 0.567797 | 0.065628 | 0.085838 | 2 | 12 |
| 44 | OTU_299 | *Pseudoxanthomonas* | 36 | 0.590476 | 0.683673 | 0.538272 | 0.181326 | 2 | 24 |
| 45 | OTU_37 | *Rhizobiales* | 11 | 0.672727 | 0.531746 | 0.029121 | 0.059336 | 3 | 10 |
| 46 | OTU_60 | *Clostridium* | 11 | 0.563636 | 0.536 | 0.03914 | 0.055027 | 3 | 10 |
| 47 | OTU_61 | *Bacteroidales* | 35 | 0.630252 | 0.676768 | 0.392084 | 0.18129 | 2 | 24 |
| 48 | OTU_77 | *Bacillales* | 10 | 0.533333 | 0.515385 | 0.037761 | 0.046025 | 3 | 9 |
| 49 | OTU_80 | AKYG1722 | 35 | 0.610084 | 0.676768 | 0.426753 | 0.177785 | 2 | 24 |
| 50 | OTU_84 | *Luteolibacter* | 12 | 0.606061 | 0.531746 | 0.040595 | 0.06255 | 3 | 11 |
| 51 | OTU_9 | SJA-15 | 17 | 0.566176 | 0.563025 | 0.101347 | 0.084972 | 3 | 12 |
| 52 | OTU_91 | *Peredibacter* | 37 | 0.566066 | 0.690722 | 0.731956 | 0.181774 | 2 | 24 |
| 53 | OTU_125 | *Myxococcales* | 11 | 0.545455 | 0.527559 | 0.038657 | 0.053162 | 3 | 10 |
| 54 | OTU_21 | *Clostridiaceae* | 10 | 0.666667 | 0.540323 | 0.023026 | 0.056254 | 2 | 9 |
| 55 | OTU_28 | *Hydrogenophaga* | 10 | 0.488889 | 0.531746 | 0.079566 | 0.045413 | 3 | 9 |
| 56 | OTU_45 | *Methylosinus* | 12 | 0.393939 | 0.51938 | 0.084639 | 0.04768 | 3 | 10 |
| 57 | OTU_47 | *Acidimicrobiales* | 13 | 0.576923 | 0.54918 | 0.072588 | 0.060391 | 3 | 10 |
| 58 | OTU_129 | *Desulfobacteraceae* | 13 | 0.782051 | 0.54918 | 0.028357 | 0.074604 | 3 | 11 |
| 59 | OTU_140 | *Koribacteraceae* | 13 | 0.461538 | 0.527559 | 0.115997 | 0.056353 | 3 | 10 |
| 60 | OTU_2 | OD1 | 9 | 0.722222 | 0.51145 | 0.022892 | 0.048482 | 3 | 9 |
| 61 | OTU_87 | *Aeromonas* | 9 | 0.694444 | 0.51145 | 0.018965 | 0.048273 | 3 | 9 |
| 62 | OTU_1 | *Sulfuricurvum* | 3 | 0.666667 | 0.458904 | 0.001011 | 0.015138 | 3 | 3 |
| 63 | OTU_16 | *Geobacter* | 11 | 0.563636 | 0.540323 | 0.048312 | 0.058247 | 3 | 10 |
| 64 | OTU_58 | Ellin6075 | 11 | 0.345455 | 0.523438 | 0.099375 | 0.043358 | 3 | 9 |
| 65 | OTU_11 | envOPS12 | 12 | 0.515152 | 0.531746 | 0.057684 | 0.055925 | 3 | 10 |
| 66 | OTU_78 | pGrfC26 | 7 | 0.428571 | 0.492647 | 0.051624 | 0.030509 | 3 | 6 |
| 67 | OTU_159 | *Spirochaetales* | 10 | 0.4 | 0.531746 | 0.062115 | 0.046849 | 3 | 10 |
| 68 | OTU_3 | *Anaerolineae* | 5 | 0.2 | 0.458904 | 0.023452 | 0.017982 | 3 | 5 |

Table S12. Estimation of network property of co-occurrence microbial network of rice cultivar SHIATS1

| Node_Index | Label | Taxa | Degree | Cluster_Coeff | Closeness | Betweenness | Eigen_Vector | Eccentricity | Coreness |
| --- | --- | --- | --- | --- | --- | --- | --- | --- | --- |
| 1 | OTU_140 | *Koribacteraceae* | 37 | 0.534535 | 0.690722 | 0.788583 | 0.184091 | 2 | 23 |
| 2 | ArcOTU_12 | *Methanosaeta* | 4 | 1 | 0.471831 | 0 | 0.026693 | 3 | 4 |
| 3 | OTU_168 | OPB54 | 40 | 0.494872 | 0.712766 | 1 | 0.191568 | 2 | 23 |
| 4 | OTU_45 | *Methylosinus* | 33 | 0.647727 | 0.663366 | 0.377274 | 0.17947 | 2 | 23 |
| 5 | OTU_83 | *Actinobacteria* | 37 | 0.567568 | 0.690722 | 0.596488 | 0.1894 | 2 | 23 |
| 6 | ArcOTU_24 | *Parvarchaea* | 15 | 0.457143 | 0.558333 | 0.183511 | 0.069079 | 3 | 9 |
| 7 | ArcOTU_2 | *Crenarchaeota* | 11 | 0.472727 | 0.523438 | 0.056008 | 0.053767 | 3 | 9 |
| 8 | ArcOTU_6 | *Methanobacterium* | 37 | 0.578078 | 0.690722 | 0.490751 | 0.190543 | 2 | 23 |
| 9 | OTU_125 | *Myxococcales* | 33 | 0.655303 | 0.663366 | 0.285422 | 0.180291 | 2 | 23 |
| 10 | OTU_187 | EB1017 | 37 | 0.552553 | 0.690722 | 0.62207 | 0.18715 | 2 | 23 |
| 11 | OTU_20 | Ellin515 | 6 | 0.533333 | 0.471831 | 0.007483 | 0.030125 | 3 | 6 |
| 12 | OTU_25 | JG30-KF-CM45 | 13 | 0.653846 | 0.54918 | 0.032558 | 0.074114 | 3 | 11 |
| 13 | OTU_60 | *Clostridium* | 9 | 0.555556 | 0.515385 | 0.022045 | 0.047005 | 3 | 9 |
| 14 | OTU_72 | *Cyanobacteria* | 30 | 0.698851 | 0.644231 | 0.250176 | 0.168418 | 2 | 23 |
| 15 | ArcOTU_3 | *Candidatus Nitrososphaera* | 38 | 0.561878 | 0.697917 | 0.614777 | 0.192786 | 2 | 23 |
| 16 | ArcOTU_23 | *Parvarchaea* | 13 | 0.538462 | 0.54918 | 0.053239 | 0.065684 | 3 | 11 |
| 17 | OTU_193 | WD2101 | 31 | 0.698925 | 0.650485 | 0.251161 | 0.175018 | 2 | 23 |
| 18 | OTU_42 | *Kouleothrixaceae* | 12 | 0.545455 | 0.536 | 0.086684 | 0.057835 | 3 | 10 |
| 19 | OTU_55 | *Syntrophobacteraceae* | 41 | 0.5 | 0.72043 | 0.988716 | 0.196514 | 2 | 23 |
| 20 | OTU_84 | *Luteolibacter* | 39 | 0.518219 | 0.705263 | 0.865956 | 0.190481 | 2 | 23 |
| 21 | OTU_87 | *Aeromonas* | 15 | 0.457143 | 0.563025 | 0.113193 | 0.072954 | 2 | 11 |
| 22 | OTU_9 | SJA-15 | 18 | 0.431373 | 0.577586 | 0.203957 | 0.083497 | 2 | 12 |
| 23 | OTU_109 | *Desulfobulbaceae* | 9 | 0.361111 | 0.507576 | 0.050914 | 0.036309 | 3 | 8 |
| 24 | OTU_129 | *Desulfobacteraceae* | 36 | 0.593651 | 0.683673 | 0.459296 | 0.187364 | 2 | 23 |
| 25 | OTU_151 | 44159 | 38 | 0.547653 | 0.697917 | 0.581109 | 0.190691 | 2 | 23 |
| 26 | OTU_159 | *Spirochaetales* | 37 | 0.548048 | 0.690722 | 0.760313 | 0.185842 | 2 | 23 |
| 27 | OTU_2 | OD1 | 6 | 0.533333 | 0.503759 | 0.009453 | 0.031622 | 3 | 6 |
| 28 | OTU_48 | DS-18 | 14 | 0.318681 | 0.544715 | 0.195584 | 0.049656 | 3 | 9 |
| 29 | OTU_50 | *Bacteroidales* | 37 | 0.567568 | 0.690722 | 0.60096 | 0.187962 | 2 | 23 |
| 30 | OTU_61 | *Bacteroidales* | 8 | 0.75 | 0.515385 | 0.008719 | 0.045691 | 3 | 8 |
| 31 | OTU_96 | LCP-6 | 37 | 0.572072 | 0.690722 | 0.489107 | 0.189708 | 2 | 23 |
| 32 | ArcOTU_8 | *Methanomicrobiales* | 34 | 0.597148 | 0.67 | 0.580528 | 0.177568 | 2 | 23 |
| 33 | OTU_1 | *Sulfuricurvum* | 19 | 0.561404 | 0.582609 | 0.144412 | 0.095967 | 2 | 12 |
| 34 | OTU_101 | *Chloroflexi* | 33 | 0.643939 | 0.663366 | 0.355267 | 0.178736 | 2 | 23 |
| Node_Index | Label | Taxa | Degree | Cluster_Coeff | Closeness | Betweenness | Eigen_Vector | Eccentricity | Coreness |
| 35 | OTU_154 | C0119 | 35 | 0.591597 | 0.676768 | 0.598476 | 0.182484 | 2 | 23 |
| 36 | OTU_189 | *Acidobacteria-6* | 34 | 0.590018 | 0.67 | 0.505376 | 0.177379 | 2 | 23 |
| 37 | OTU_21 | *Clostridiaceae* | 15 | 0.609524 | 0.553719 | 0.065346 | 0.074869 | 3 | 12 |
| 38 | OTU_24 | *Thermoleophilia* | 13 | 0.615385 | 0.540323 | 0.063898 | 0.070746 | 3 | 10 |
| 39 | OTU_299 | *Pseudoxanthomonas* | 35 | 0.588235 | 0.676768 | 0.542972 | 0.181 | 2 | 23 |
| 40 | OTU_32 | Gemm-5 | 7 | 0.619048 | 0.492647 | 0.012402 | 0.033626 | 3 | 7 |
| 41 | OTU_428 | BPC076 | 40 | 0.50641 | 0.712766 | 0.901826 | 0.194049 | 2 | 23 |
| 42 | OTU_44 | *Aeromonadaceae* | 15 | 0.542857 | 0.558333 | 0.0925 | 0.076681 | 3 | 11 |
| 43 | OTU_58 | *Ellin6075* | 6 | 0.466667 | 0.471831 | 0.010364 | 0.028061 | 3 | 6 |
| 44 | OTU_77 | *Bacillales* | 31 | 0.696774 | 0.650485 | 0.256894 | 0.174783 | 2 | 23 |
| 45 | OTU_89 | S035 | 16 | 0.541667 | 0.558333 | 0.145224 | 0.07919 | 3 | 12 |
| 46 | OTU_91 | *Peredibacter* | 32 | 0.689516 | 0.656863 | 0.201375 | 0.179374 | 2 | 23 |
| 47 | OTU_95 | *Algoriphagus* | 14 | 0.659341 | 0.558333 | 0.037966 | 0.075001 | 2 | 12 |
| 48 | OTU_10 | *Saprospiraceae* | 17 | 0.426471 | 0.567797 | 0.20615 | 0.07352 | 3 | 12 |
| 49 | OTU_11 | envOPS12 | 13 | 0.589744 | 0.544715 | 0.044746 | 0.066222 | 3 | 12 |
| 50 | OTU_121 | *Sinobacteraceae* | 13 | 0.564103 | 0.544715 | 0.087171 | 0.065076 | 3 | 11 |
| 51 | OTU_185 | *Candidatus Entotheonella* | 14 | 0.615385 | 0.553719 | 0.079717 | 0.071831 | 3 | 12 |
| 52 | OTU_47 | *Acidimicrobiales* | 14 | 0.725275 | 0.54918 | 0.026858 | 0.080684 | 3 | 12 |
| 53 | OTU_6 | *Anaerolinea* | 11 | 0.436364 | 0.515385 | 0.062733 | 0.045624 | 3 | 9 |
| 54 | OTU_78 | pGrfC26 | 10 | 0.466667 | 0.515385 | 0.079589 | 0.043387 | 3 | 9 |
| 55 | ArcOTU_7 | *Methanocella* | 13 | 0.653846 | 0.54918 | 0.037875 | 0.0704 | 3 | 12 |
| 56 | OTU_124 | *Gaiellaceae* | 17 | 0.477941 | 0.558333 | 0.115677 | 0.079259 | 3 | 12 |
| 57 | OTU_54 | Ellin6529 | 11 | 0.363636 | 0.523438 | 0.08124 | 0.045693 | 3 | 9 |
| 58 | OTU_15 | *Anaerolineae* | 11 | 0.418182 | 0.527559 | 0.112694 | 0.047882 | 3 | 9 |
| 59 | OTU_57 | *Geobacteraceae* | 14 | 0.494505 | 0.540323 | 0.100494 | 0.065871 | 3 | 11 |
| 60 | OTU_80 | AKYG1722 | 12 | 0.318182 | 0.51145 | 0.140232 | 0.044694 | 3 | 9 |
| 61 | OTU_16 | *Geobacter* | 7 | 0.380952 | 0.5 | 0.038736 | 0.02724 | 3 | 7 |
| 62 | OTU_22 | *Chitinophagaceae* | 9 | 0.694444 | 0.523438 | 0.01249 | 0.051388 | 3 | 9 |
| 63 | OTU_28 | *Hydrogenophaga* | 8 | 0.428571 | 0.503759 | 0.039079 | 0.03354 | 3 | 8 |
| 64 | OTU_37 | *Rhizobiales* | 6 | 1 | 0.503759 | 0 | 0.039753 | 3 | 6 |
| 65 | OTU_137 | *Lachnospiraceae* | 11 | 0.436364 | 0.527559 | 0.073776 | 0.044199 | 3 | 9 |
| 66 | OTU_146 | *Rhodobacteraceae* | 9 | 0.416667 | 0.507576 | 0.062589 | 0.035077 | 3 | 8 |
| 67 | OTU_3 | *Anaerolineae* | 5 | 0.3 | 0.471831 | 0.017373 | 0.018981 | 3 | 5 |
| 68 | OTU_49 | *Rhodospirillales* | 9 | 0.861111 | 0.531746 | 0.005526 | 0.056169 | 3 | 9 |
